# Defining the Mechanism of Action and Resistance of New *Mycobacterium abscessus* MmpL3 Inhibitors

**DOI:** 10.1021/acschembio.5c00709

**Published:** 2026-01-13

**Authors:** Bassel J. Abdalla, Matthew B. Giletto, Nazli Goksel Carpa, Angela K. Wilson, Edmund Ellsworth, Robert B. Abramovitch

**Affiliations:** † Department of Microbiology, Genetics & Immunology, 3078 Michigan State University, East Lansing, Michigan 48824, United States; ‡ Department of Pharmacology and Toxicology, Michigan State University, East Lansing, Michigan 48824, United States; § Department of Chemistry, Michigan State University, East Lansing, Michigan 48824, United States

## Abstract

*Mycobacterium abscessus* (Mab) is difficult to treat due to intrinsic and acquired resistance to diverse antibiotics. Among the intrinsic resistance factors is the mycomembrane, a complex structure that limits permeability to several classes of antibiotics. Here, we report new inhibitors of MmpL3, an essential transporter required to build the mycomembrane. Several of the MmpL3 inhibitors have comparable activity in vitro to standard-of-care treatments, exhibit both time- and dose-dependent bactericidal activity, have low eukaryotic cytotoxicity, and are efficacious against Mab growing in macrophages or in biofilms. The inhibitors had varying activities against a panel of 30 different multidrug-resistant clinical isolates and are additive or synergistic with standard-of-care antibiotics, suggesting they could be included in combination therapy. The inhibitors also exhibit a low frequency of resistance, with some of the isolated mutants displaying differential patterns of sensitivity and resistance to the different MmpL3 inhibitors and putative fitness defects. Cross-resistance profiles of 15 structurally related inhibitors against 16 different MmpL3 resistant mutants demonstrate structure-driven clustering patterns of the inhibitors, where those carrying a similar scaffold cluster together and different MmpL3 amino-acid substitutions account for these differences. Cross-resistance profiles were also simulated computationally, showing significant correlation between the computationally calculated parameters and the biological patterns of cross-resistance and emphasizing specific structural–functional associations driving resistance or susceptibility. These inhibitors and their analogs hold promise for clinical translation, and the established structural–functional associations provide mechanistic insights into the function of MmpL3, resistance and susceptibility of MmpL3 inhibitors, and fitness costs associated with MmpL3 resistance.

## Graphical abstract

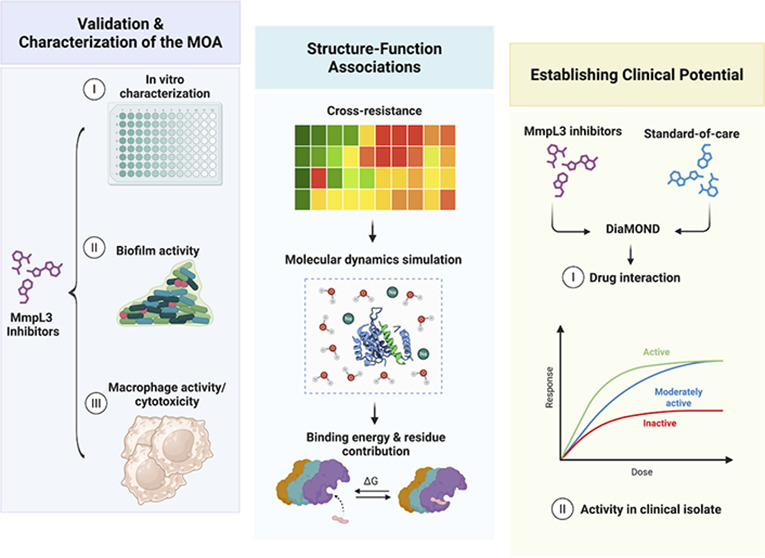

## Significance Statement

Mab is a challenging-to-treat nontuberculous mycobacterial (NTM) infection, with growing prevalence and intrinsic multidrug resistance. MmpL3, an inner membrane transporter of trehalose monomycolate, is an extensively studied drug target because it is essential for mycobacterial survival. In this study, we define the mechanism of action of a panel of novel MmpL3 inhibitors. Cross-resistance studies between MmpL3 resistant mutants and inhibitors, followed by computational simulations of the analogs’ binding and energetics, link structural aspects of the protein–ligand interactions with functional mechanisms of inhibition, resistance, susceptibility, and fitness. This study reveals structural and functional features of MmpL3 inhibition and resistance that will accelerate the development of new MmpL3 inhibitors to treat challenging Mab infections.

## Introduction

The mycobacterial genus harbors several species that cause serious infections, including *Mycobacterium tuberculosis* (**Mtb**) complex bacteria that cause tuberculosis disease, *M. leprae* that causes leprosy, and a collective group of bacteria called nontuberculous mycobacteria that cause diverse and severe pathologies, mainly in the lungs but also extrapulmonary.^1–3^ NTM species, such as the fast-growing *M. abscessus* complex (**Mab**) and the slow-growing *M. avium* complex (**MAC**), target healthy individuals as well as those with preexisting comorbidities such as immunodeficiency, cystic fibrosis, and bronchiectasis.^4,5^ While mycobacteria are known to be insensitive to most known antibiotics, NTMs are infamous for their drug resistance. Resistance mechanisms vary between intrinsic (e.g., drug-modifying enzymes) and extrinsic (e.g., acquired target mutations),^6–8^ and together make Mab a clinically challenging mycobacterium to treat, with cure rates <50% even with several years of multidrug regimens.^9–11^

The mycobacterial cell envelope provides intrinsic resistance to many antibiotics The mycomembrane structure is rich in complex lipids such as mycolic acids (**MAs**), which constitute more than half of the bacteria’s dry mass and exhibit highly hydrophobic waxy features that cause its impermeability to polar substances, including many antibiotics.^12–15^ This has rendered the mycobacterial cell envelope a target for several antimycobacterial drug classes (e.g., imipenem and meropenem for the peptidoglycan component and ethambutol for the arabinogalactan component^16,17^ ). Since mycolic acid is the primary constituent driving impermeability to several antibiotics, its synthesis and transfer pathways are well-studied for potential drug targets.^18^ The mycobacterial membrane protein large 3 (**MmpL3**) has been a focus of drug discovery efforts for its essential role in transferring trehalose monomycolate, a mycolic acid ester and a major constituent of the mycomembrane, from the cytoplasm, where it is synthesized, to the mycomembrane where it functions.^19–21^ MmpL3 is also known to interact with and transfer several other membrane lipids,^22^ adding to its value as a target. However, less is known about the protein structure and how it influences inhibitor binding, interaction dynamics, and mechanisms of resistance. Thus, understanding the genetic and biochemical basis of MmpL3 susceptibility and resistance to inhibition by small molecules could inform a rational drug discovery approach.

Due to the degree of amino-acid similarity between MmpL3 in Mab (MAB_4508) and its ortholog in Mtb (Rv0206c) (56%),^23^ MmpL3 inhibitors have been discovered in both species. Several classes (e.g., indole-2-carboxamides,^24–26^ benzimidazoles,^27,28^ benzothiazoles,^29^ piperidines,^30^ acetamides,^23^ and thiophene-4-carboxamide^31,32^ ) are often active against both species, while some are exclusive to one species or the other (e.g., pyrroles/pyrazoles (Mtb),^33^ benzofuran (Mab),^26^ quinolones (Mtb),^34^ and naphthalenes (Mtb)^35^). Notably, SQ109, an ethane-1,2-diamine orphan drug and the only MmpL3 inhibitor to reach clinical trials for the treatment of Mtb, is completely inactive against Mab. A few SQ109 derivatives were shown to exhibit minimal activity in Mab in a recent study. Therefore, the discovery of MmpL3 inhibitors with similar clinical potential in Mab is important to expand the pipeline of antimycobacterials effective against NTMs.^36,37^

Here, we describe the identification and characterization of the mode of action (MoA) of 3 sets of analogs of MmpL3 inhibitors, analogs of HC2091 and HC2099, two scaffolds previously described by Zheng et al. and Williams et al.,^31,32,38^ and mixed series analogs carrying pharmacophores of both scaffolds, all selected based on their enhanced in vitro activity compared to the other analogs and parent scaffolds. We additionally present a forward genetic selection against the panel of analogs that yielded 16 *mmpL3* mutants, presenting different cross-resistance patterns to the analogs and exhibiting putative fitness defects associated with the mutations. We leverage molecular dynamics simulations to study how a few analogs interact and bind with selected MmpL3 mutants. These simulations focus on how mutations alter protein structure, changing pocket size, solvent accessibility, and residue–ligand interactions, leading to variable cross-resistance patterns and offering new insights into the structure–function relationship of MmpL3 and its inhibitors. We also establish the clinical potential of the inhibitors by examining their activity against 30 multidrug-resistant clinical isolates and their pairwise positive drug interaction profiles (i.e., synergism and additivity) with several standard-of-care treatments. We finally present the transcriptomic signature of MmpL3 inhibition in Mab, examining trends in the relevant biochemical and metabolic pathways regulated by MmpL3 inhibition.

## Results

### Characterization of the Modes of Action of the MmpL3 Inhibitor Panel

HC2091 and HC2099 were initially shown to have activity against Mab,^31,32,38^ with analogs of HC2091 showing higher potency than HC2099. Synthesis of HC2091 analogs identified new compounds with further enhanced activity against Mab, including **MSU-43644** (*N*-[2-(4-chlorophenyl)­ethyl]-1-methylcyclohexane-1-carboxamide), **MSU-44147** (1-methyl-*N*-{2-[4-(trifluoromethyl)­phenyl]­ethyl}­cyclohexane-1-carboxamide), **MSU-45431** (1-ethyl-*N*-{2-[4-(trifluoromethyl)­phenyl]­ethyl}­cyclohexane-1-carboxamide), **MSU-45683** (*N*-{2-fluoro-2-[4-(trifluoromethyl)­phenyl]­ethyl}-1-(trifluoromethyl)­cyclopentane-1-carboxamide), and **MSU-45518** (1-(trifluoromethyl)-*N*-{3-[4-(trifluoromethyl)­phenyl]­cyclobutyl}­cyclopentane-1-carboxamide). By combining elements of HC2091 and the HC2099 analog, **MSU-43085** (3-(5,6-dichloro-1*H*-1,3-benzodiazol-2-yl)-*N*,*N*-bis­(propane-2-yl) propenamide),^38^ we also developed a mixed series of both scaffolds, starting with **MSU-43557** (*N*-(5,6-dichloro-1*H*-1,3-benzimidazol-2-yl)-1-methylcyclohexane-1-carboxamide) (Figure 1a). This panel of inhibitors was prioritized for the study based on their enhanced in vitro activity and characterized for activity against Mab ATCC 19977. The inhibitors had half-maximal effective concentrations (EC_50_s) ranging from 150 nM to 4.76 μM in vitro (Figure 1b, Table 1), which is comparable to the standard-of-care treatments (Figure S1). The panel exhibits time-dependent bactericidal activity with kinetics similar to those of amikacin and bedaquiline (Figure 1c). Additionally, the inhibitors display putative intracellular bactericidal activity in bone marrow-derived macrophages (BMMΦ) infected with Mab (pmV261 hsp60::mEmerald). The intracellular EC_50_ of the panel ranges from 70 nM to 6.5 μM (Table 1, Figure S2), which is several orders of magnitude less than their cytotoxicity (>80–32 μM, Figure S3) and comparable to the intracellular efficacy of clarithromycin and rifabutin (Table 1, Figure S2). Together, these data show the analogs have in vitro activities comparable to or better than standard-of-care drugs.

**Figure 1:**
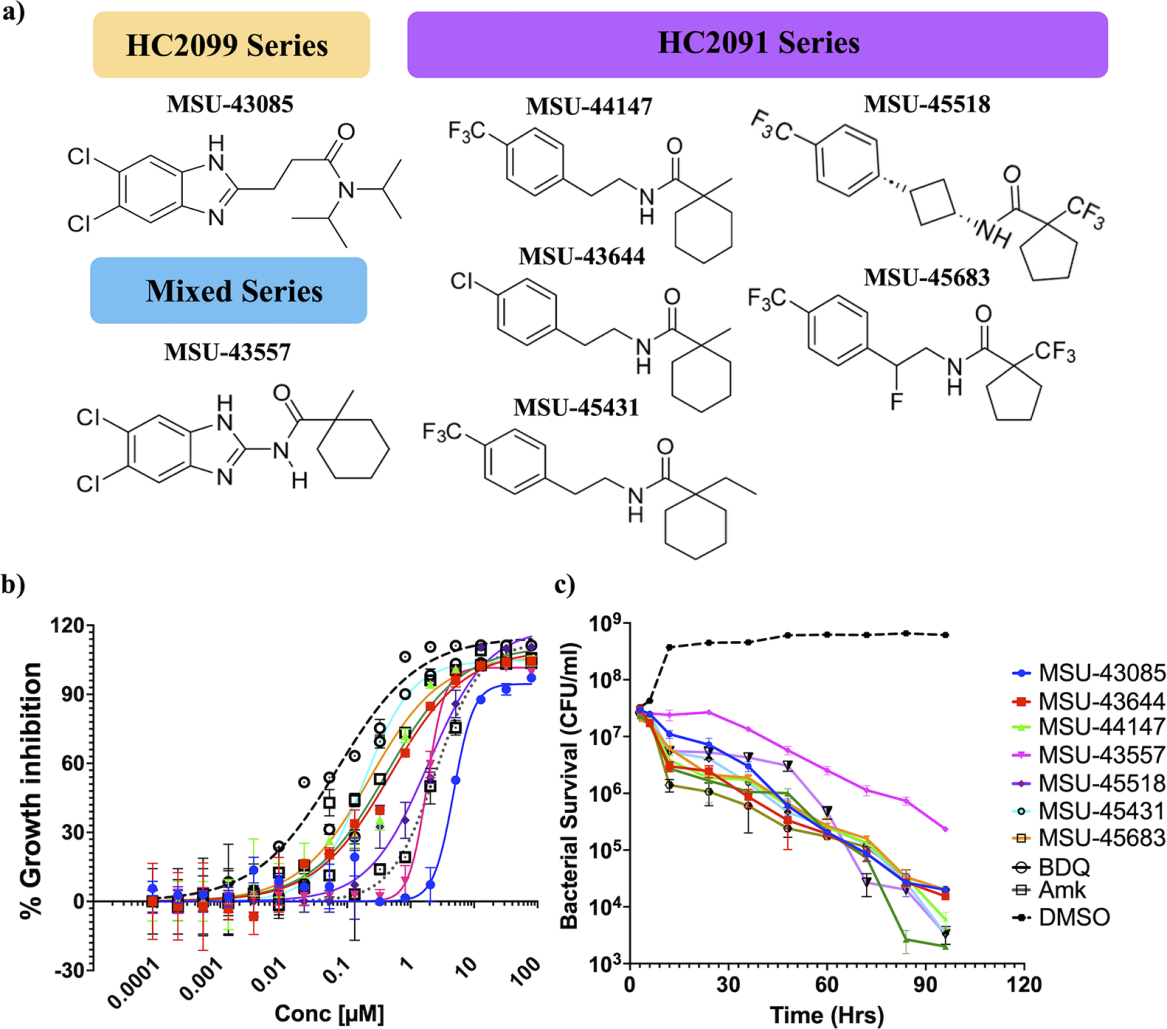
Seven representative MmpL3 inhibitors of three series inhibit *Mab* growth in a dose- and time-dependent manner. (a) Structure of the seven MmpL3 inhibitor analogs categorized by parent scaffold. (b) MmpL3 inhibitor analogs inhibit Mab growth in a dose-dependent manner and have comparable activities to amikacin and bedaquiline. (c) The MmpL3 inhibitor analogs kill Mab in a time-dependent manner when treated at 5× of the minimum inhibitory concentration (MIC). The graphs show *Mab* treated with the seven analogs, bedaquiline (BDQ) and amikacin (AMK), as positive controls and DMSO as a negative control. Data is pooled from two independent experiments (b) or a representative of three independent experiments (c).

**Table 1:** Characterization of MmpL3 Inhibitors’ Mode of Action in *Mab* in Comparison with Standard-of-Care Treatments^a^

	EC_50_ [μM]	MIC [μM]	MIC [μg/mL]	EC_50_ in BMDϕ [μM]	Δψ disruption	NRK	biofilm disruption [μM]	viability in Biofilm [μM]	FoR @ 3× MIC	CC_50_ [μM]
MSU-43085	4.76	10.55	3.61	6.43	no^b^	no	12.89	0.32	2.8 × 10^–6^	>80
MSU-43644	0.44	9.15	2.56	0.42	no	no	3.37	0.58	3.5 × 10^–7^	>80
MSU-44147	0.46	8.15	2.55	0.26	no	no	1.96	0.18	3.7 × 10^–7^	>80
MSU-43557	1.29	3.69	1.20	0.39	no	no	8.66	0.16	3.1 × 10^–7^	>80
MSU-45518	0.15	16.93	6.42	0.23	no	no	ND	1.17	5.3 × 10^–6^	>32
MSU-45431	0.20	1.28	0.42	0.07	no	no	1.01	0.035	1.2 × 10–8	>80
MSU-45683	0.21	3.79	1.41	0.23	no	no	2.00	0.36	1.7 × 10^–8^	>80
amikacin	2.40	10.92	6.39	NT	NT	NT	3.04	0.05	NT	NT
clarithromycin	0.02	0.73	0.55	0.34	NT	NT	3.92	0.09	NT	NT
rifabutin	0.12	2.02	1.71	0.11	NT	NT	NT	NT	NT	NT
tigecycline	0.69	2.03	1.19	NT	NT	NT	1.67	0.26	NT	NT
bedaquiline	0.06	2.2	1.22	NT	NT	NT	0.63	0.04	NT	NT

a Activity (EC_50_ and MIC), putative intracellular killing efficacy in macrophages (Mϕ) (see Figure S2), membrane potential (Δψ) disruption (see Figure S5), nonreplicative killing (NRK) (see Figure S6), EC_50_ for biofilm disruption and reduction of viability inside biofilms (see Figure S7), Frequency of Resistance (FoR), and eukaryotic cytotoxicity (CC_50_) in murine bone marrow-derived macrophages (BMMΦ) (see Figure S3). *n* ≥ 1, *m* = 3.

b MSU-43085 shows a membrane potential dissipation activity only at the highest used concentrations. NT: not tested, ND: not determined.

We next sought to validate the proposed mechanism of action and characterize the biological activities of the compounds, including their ability to disrupt membrane potential, kill nonreplicating Mab, disrupt or kill Mab growing in biofilms, and act against bacteria in other species. HC2091 and HC2099 have been shown to directly inhibit MmpL3 in Mtb, including by directly binding the MmpL3 protein in a probe displacement assay and inhibition of TDM synthesis.^38^ To biochemically validate their mechanism of action in Mab, total lipids were extracted from Mab cultures treated with 5× the MIC of the aforementioned analogs and analyzed using thin-layer chromatography (TLC) (Figure S4). The presence of TDM was observed at high levels in the DMSO-treated cells and was almost undetectable in the cultures treated with MmpL3 inhibitors, providing biochemical evidence for the inhibition of MmpL3 function as the mode of action in Mab, as we previously observed in Mtb. Some MmpL3 inhibitors cause the loss of membrane potential, possibly due to their disruption of the proton-relay Asp–Tyr pairs in the MmpL3 binding pocket. For the tested analogs, the inhibitors do not strongly dissipate membrane potential in Mab (Figure S5). Only MSU-43085, an HC2099 analog, slightly dissipated membrane potential at high concentrations (160 μM), consistent with what we previously reported^31,38^ (Table 1, Figure S5a). Disruption of membrane potential is associated with the ability of MmpL3 inhibitors to kill nonreplicating mycobacteria, and indeed, the compounds lacked bactericidal activity in nonreplicating Mab (Table 1, Figure S6) when examined in a starvation-induced dormancy model.^39,40^ Mab can grow in biofilms, and we examined whether MmpL3 inhibitors can disrupt biofilm formation and kill bacteria when growing in biofilms. The analogs exhibited biofilm-disrupting activities in the mature submerged biofilm model, except for MSU-45518, and are capable of penetrating the biofilm matrix and killing bacteria with activities comparable to standard-of-care treatments such as amikacin, bedaquiline, clarithromycin, and tigecycline (Table 1, Figure S7). We also examined the activity of the analogs against a panel of other bacterial species and observed a very narrow spectrum of activity, exclusive to mycobacteria, i.e., Mtb, Mab, and *M. smegmatis* (Msmeg)^31^ (Table 2, Figure S8), a finding consistent with MmpL3 being an exclusive target to mycobacteria, demonstrating the putative selectivity of the analogs to the target.

**Table 2:** Spectrum of Activity of MmpL3 Inhibitors (EC_50_ Values in μM) Showing a Very Narrow Spectrum of Activity That Is Exclusive to *Mycobacteria* (Figure S8)­^a^

	MSU-43085	MSU-43644	MSU-44147	MSU-43557	MSU-45518	MSU-45431	MSU-45683
*M. tuberculosis* **(Erdman)**	0.17	1.17	0.26	0.17	0.61	0.23	0.06
*M. tuberculosis* **(CDC1551)**	0.23	0.25	0.19	0.19	0.55	1.05	0.05
*M. tuberculosis* **(H*37Rv*)**	0.19	0.49	0.19	0.13	0.38	0.73	0.05
*M. abscessus*	4.76	0.44	0.46	1.82	1.88	0.20	0.21
*M. smegmatis*	3.93	15.54	4.16	2.54	17.01	0.86	14.57
*E. coli*	>80	>80	>80	>80	>80	>80	>80
*P. vulgaris*	>80	>80	>80	>80	>80	>80	>80
*E. faecalis*	>80	>80	>80	>80	>80	>80	>80
*P. aeruginosa*	>80	>80	>80	>80	>80	>80	>80
*S. aureus* **ATC25923**	>80	>80	>80	>80	>80	>80	>80
*S. aureus* **ATC29213**	>80	>80	>80	>80	>80	>80	>80

a *n* ≥ 2, *m* = 3.

### Cross-Resistance Profiles of the Mutant and Inhibitor Panels Indicate Specific Protein–Ligand Interactions Drive Resistance and Sensitivity

To study the structure of MmpL3 and how it reflects the binding and structure–activity relationship (SAR) of the analogs, we conducted a forward genetic selection to isolate resistant mutants against selected analogs and study their resistance/sensitivity patterns to the different analogs via dose–response assays. In the selection, the analogs display a relatively low frequency of resistance for Mab in the 10^–7^ to 10^–8^ range, except MSU-43085 and MSU-45518, which have a frequency of resistance at 10^–6^ (Table 1). In total, 123 resistant mutants were isolated and confirmed against the analogs they were isolated against via dose–response assays, the genomic DNA of 62 of which was sequenced, and 16 distinct mutations in *mmpL3* (MAB_4508) were identified (Table S1).

We hypothesized that by examining differences in resistance and susceptibility between the different mutants and analogs, we can refine the structure–activity relationships of the ligands and study the MmpL3 pocket influence on the mechanisms of the compounds. That is, we anticipate that structural aspects such as specific amino substitutions in MmpL3 and their interactions with specific R-groups on the analogs will be associated with functional aspects of resistance or susceptibility. Toward this goal, we expanded the diversity of analogs to examine the impact of specific R-groups on susceptibility or resistance in the panel of 16 resistant mutants. In addition to the panel of 7 analogs described above, 8 more analogs spanning different substitutions on the parent scaffold (Figures 2a and S9c) with comparable activities in Mab (Figure S9) were included in the cross-resistance study. The list includes additional HC2099 analogs such as **MSU-45655** (3-(5-chloro-6-nitro-1*H*-1,3-benzimidazol-2-yl)-*N*,*N*-di­(propan-2-yl)­propanamide), **MSU-43186** (1-(azepan-1-yl)-3-(5,6-dichloro-1*H*-1,3-benzimidazol-2-yl)­propan-1-one), **MSU-45540** (3-[5,7-bis­(trifluoromethyl)-1*H*-1,3-benzimidazol-2-yl]-*N*,*N*-di­(propan-2-yl)­propanamide), **MSU-45516** (3-(5,6-dichloro-1*H*-1,3-benzimidazol-2-yl)-*N*-(1,1,1-trifluoropropan-2-yl)-*N*-methylpropanamide), and **MSU-45538** (*N*-cyclopentyl-3-(5,6-dichloro-1*H*-1,3-benzimidazol-2-yl)-*N*-ethylpropanamide) and mixed series analogs like **MSU-45606** (1-(chloromethyl)-*N*-(5,6-dichloro-1*H*-1,3-benzimidazol-2-yl)­cyclohexane-1-carboxamide), **MSU-45819** (*N*-(5-trifluoromethoxy-1*H*-1,3-benzimidazol-2-yl)-1-(trifluoromethyl)­cyclohexane-1-carboxamide), and **MSU-45350** (*N*-(5,6-dibromo-1*H*-1,3-benzimidazol-2-yl)-1-ethylcyclohexane-1-carboxamide). These additional analogs exhibited EC_50_s in the range of 0.19 to 1.8 μM (Figure S9a).

**Figure 2:**
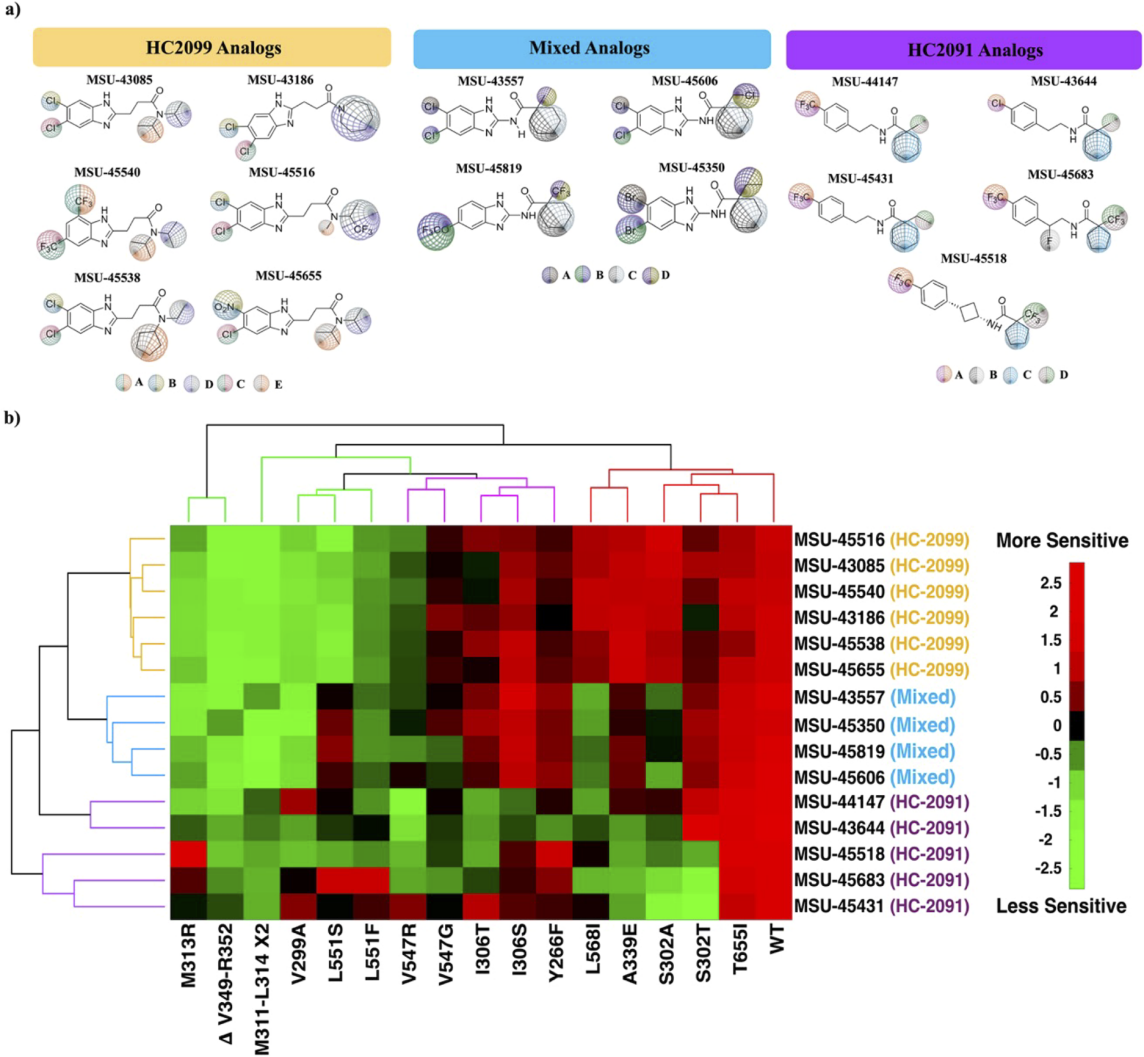
Characterization of the activity of selected analogs against *Mab mmpL3* mutants reveals complex sensitivity/resistance patterns and hints at structure–function associations. (a) Structure of 15 representative MmpL3 inhibitors of three series, categorized by parent scaffold. The different substitution sites (A, B, C, D, and E) are differentially labeled with colored globes of different sizes, each with a distinct color signature, emphasizing the differences of the substitutions at a particular site on the parent scaffold. (b) Cluster analysis of the cross-resistance between analogs in panel a with 16 *mmpL3* mutant strains and the WT (see Table S1). The color scale is based on the *Z*-scores of the treated strains, where green indicates less activity of the analog and red indicates more activity of the analog, all with reference to the mean activity (black). Compounds and mutations are clustered into different clades, signifying the parent scaffold and the mutations’ impact on drug binding and efficacy. Clades of the analogs and mutations are color-coded to match the label of the parent scaffold and the location/impact of the mutation. *n* = 3, *m* = 3.

We examined the susceptibility of the panel of 16 mutants against the 15 analogs by examining dose–response curves. As anticipated, many mutants exhibited nonideal dose–response patterns owing to their resistance (i.e., the dose–response relationship does not follow the standard sigmoidal curve), so an EC_50_ could not be used as a reference for comparison. Instead, the areas under the curve (AUC) of every mutant–analog combination were calculated (Table S2), as previously described.^31^ To avoid the impact of the varying potencies of the analogs in the WT, we normalized the calculated AUCs of the mutants treated with one analog to that of the WT treated with the same analog and standardized the normalized AUCs by calculating the *Z*-scores, which were analyzed using hierarchical cluster analysis. In the resulting clustergram (Figure 2b), analogs and resistant mutants (denoted by their amino-acid substitutions) group into different clades. Analogs cluster based on their SAR into three clades representing the three series of inhibitors, showing that structural elements of the ligands are driving their patterns of resistance and susceptibility. Mutations also cluster into three clades: clade A, which contains pan-resistant mutations to all three scaffolds; clade B, which exhibits specificity to HC2091 analogs; and clade C, which displays resistance to HC2091 and the mixed series but not HC2099 analogs.

Since mutations frequently come with a fitness cost, the fitness of the 16 mutants, in terms of relative growth rate to the WT, was assessed. Growth curves were generated over 5 days, and AUCs of the growth curves were calculated (Figure S10), normalized to the WT, and plotted in a beehive graph (Figure S11). The entire panel exhibits lower growth rates than the WT, hinting toward a putative fitness defect and a role of the mutated residues in the function of MmpL3. However, it is important to note that the mutations leading to putative fitness defects need to be re-established in an isogenic background with the WT to eliminate the possibility of a confounding background mutation contributing to the observed phenotype. Under the assumption that a fitness defect-driving mutation would compromise the bacteria against other treatments, a subset of mutants presenting major fitness defects was tested for sensitivity against commonly prescribed standard-of-care treatments (i.e., clarithromycin and amikacin) and those targeting the cell envelope (i.e., meropenem). Several of these mutants displayed more sensitivity toward the drugs, especially meropenem (Figure S12), hinting at the impact of fitness-altering mutations conferring resistance to MmpL3 inhibitors on drug sensitivity to the standard-of-care treatments and the compound effect of envelope-targeting agents in MmpL3 mutants.

Mutations were modeled to the crystal structure, which was generated by homology-based modeling of the primary structure of Mab MmpL3 against the crystal structure of *M. smegmatis* (PDB: 6AJH),^41^ using the SWISS-MODEL online server.^42^ As shown in Figure 3b, mutations are color-coded to match the clades in Figure 2b. Clade A harbors mutations that yield dramatic perturbations in the protein structure, disregarding their proximity to the binding pocket. These include insertion and deletion mutations, as well as amino-acid substitutions with a major difference in size or polarity (e.g., M313R and L551F). However, mutations with proximity to the pocket are clustered in clades B and C, most of which are lining the central vestibule (i.e., binding pocket), suggesting a role in protein–ligand interactions.^43^

**Figure 3:**
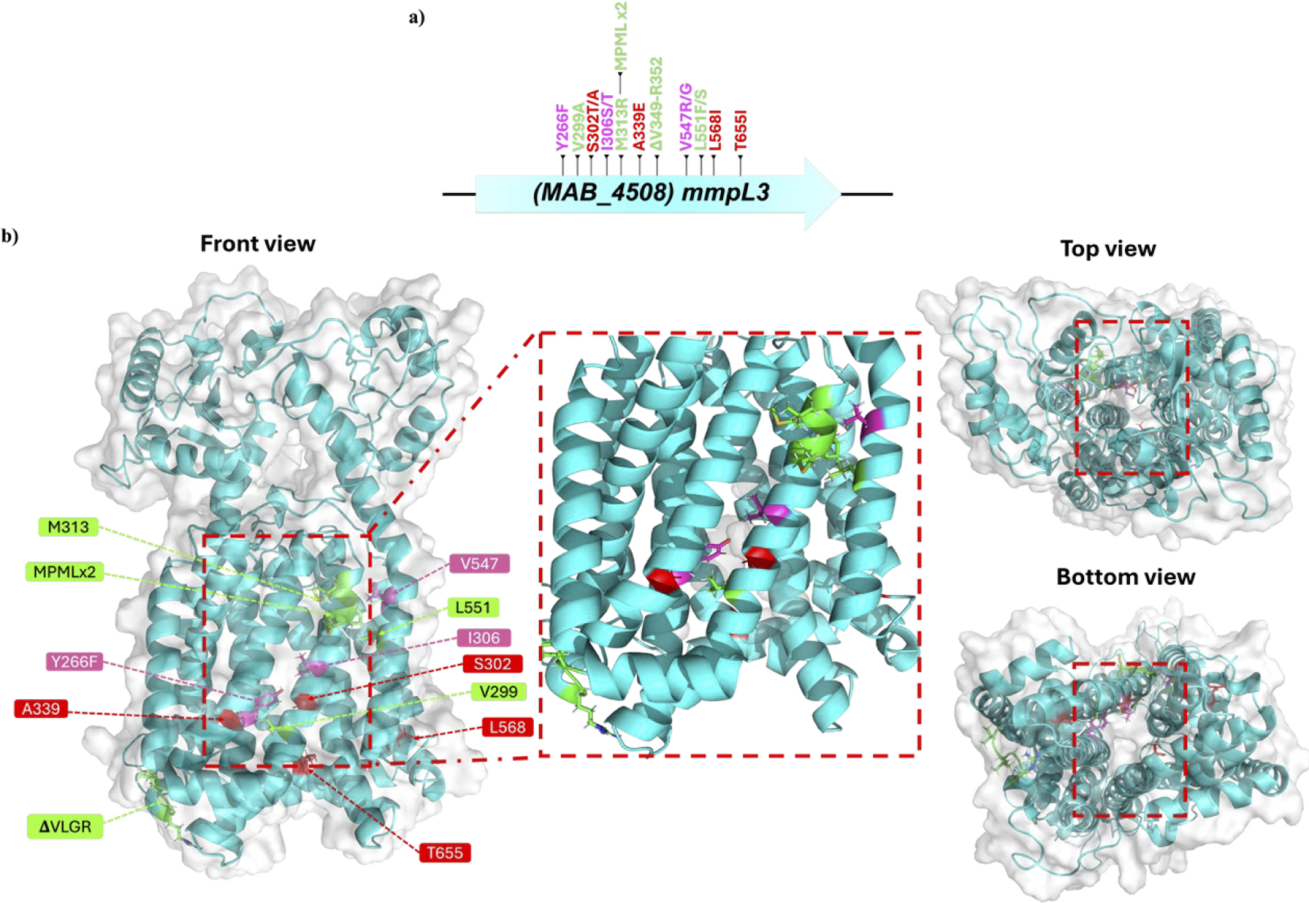
2D and 3D representation of the isolated mutations in MmpL3 (MAB_4508) in *Mab*, depicting how structural aspects of the protein and the binding pocket reflect functional aspects regarding MmpL3 inhibition, resistance, and sensitivity. (a) 2D representation of the 16 isolated mutations in MmpL3 of *Mab*. (b) 3D representation of the mutated residues showing their distribution across the protein structure. Residues are color-coded to reflect functional associations (i.e., level of resistance induced against a particular analog by mutating a particular residue), showing a correlation between the extent of structural perturbations induced by the mutations, their position relative to the binding pocket, and the associated level of resistance or sensitivity. Green indicates pan-resistance to all analogs (cluster 1 in Figure 2), while magenta and red represent series-specific resistance (clusters 2 and 3 in Figure 2).

### In Silico Modeling Reveals Common Patterns for Binding Energetics and Cross-Resistance Profiles

A subset of mutants, shown in Figure 4, was selected for molecular docking calculations and molecular dynamics simulations to examine ligand–pocket interactions, pocket size, solvent accessibility, and residue contribution to the binding energy. We hypothesized that the modeled interactions of analogs and mutants will recapitulate patterns of resistance and sensitivity observed in whole cells. The best docking poses of a few analogs were aligned in the binding pocket (Figure 4a), portraying their capacity to differentially engage subpockets and interact with nearby residues of MmpL3. A few mutations (e.g., S302A, V299A, and M313R) are selectively presented here as a proof of concept of the structure–function associations of the target and the inhibitors and how they drive cross-resistance.

**Figure 4:**
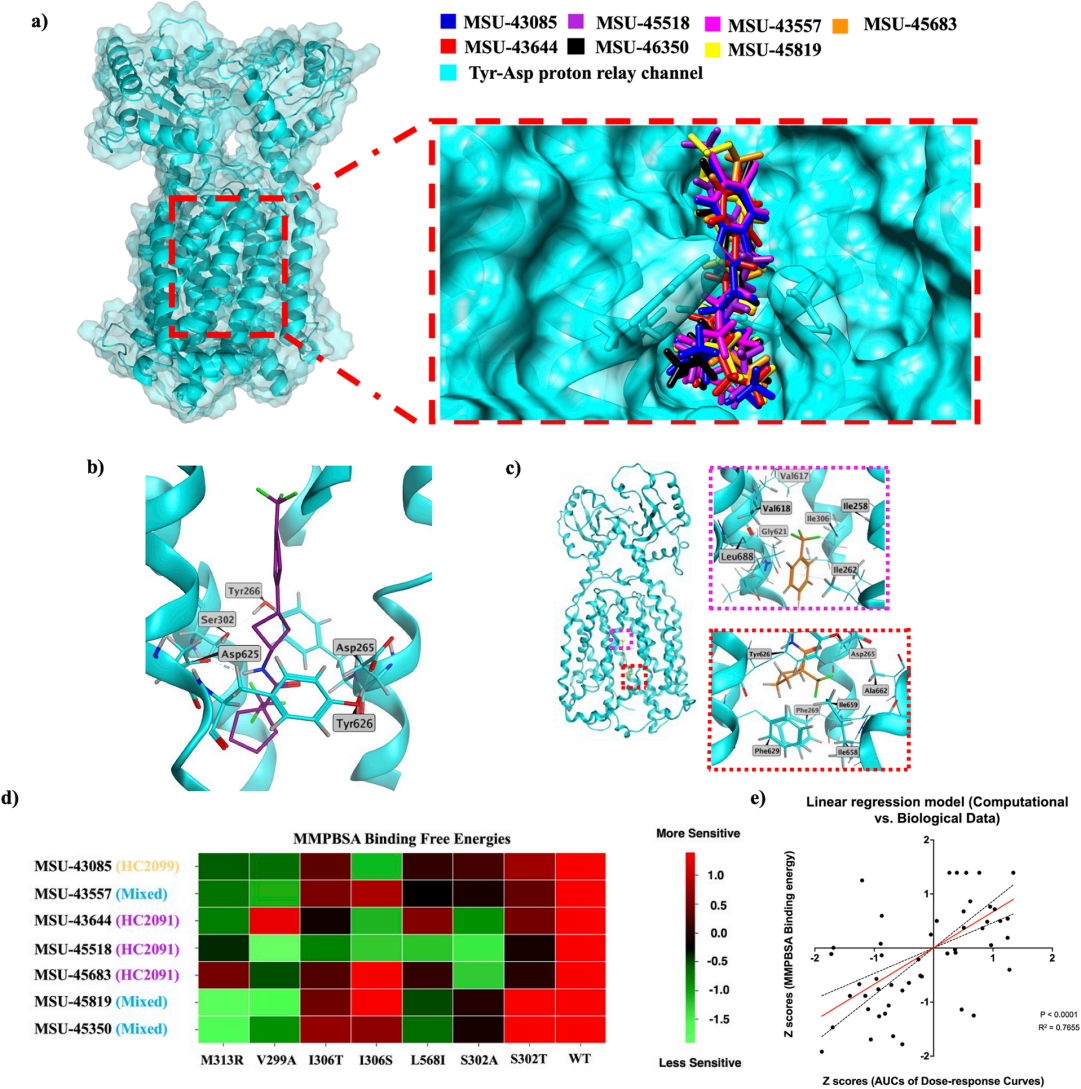
In silico simulation of the binding of different MmpL3 inhibitors (by scaffold) and binding energetics from molecular dynamics simulations of different analog MmpL3-mutant combinations, mirroring the biological data. (a) Best-scoring docking poses of MmpL3 inhibitor analogs, color-coded and aligned in the active site with the tyrosine-aspartate proton relay channel. (b) A snapshot taken from the end of the simulation trajectory with MSU-45518 and wild-type MmpL3 protein of *Mab* showing a hydrogen bond interaction between the amide of the ligand and ASP625, disrupting the hydrogen-bond network around MSU-45518. (c) A snapshot taken from the end of the simulation trajectory of MSU-45683 and wild-type MmpL3 protein of *Mab,* showing the different residues interacting with the trifluoromethyl ends of the ligand. Residues within 4.5 Å of the fluorine atoms of the two CF_3_ at the two ends of MSU-45683 were selected and labeled in MOE. (d) Cluster analysis of computationally predicted cross-resistance patterns showing that the Z-scores calculated based on the predicted binding energetics (MMPBSA) mostly match those from the observed biological activities (AUCs of dose–response curves) and that the clustering patterns and mutation distribution in clades are similar to the biological data (see Figure 2b). (e) A linear regression model depicting the correlation between *Z*-scores of computational parameters (i.e., binding energies) and biological parameters (i.e., AUCs of cross-resistance dose–response curves). *P* < 0.0001, *R* ^2^ = 0.7655. Since all the WT data points were normalized to themselves, they were not included in the linear regression analysis.

Throughout the simulation trajectory of the wild-type apo protein, the proton relay residues D265, Y266, D625, and Y626, as well as S302, display extensive connectivity via a hydrogen-bond network (Figure 4b), while hydrophobic residues in the upper and lower parts of the pocket (i.e., I262, I258, I306, V618, G621, L688, F269, and F629) were shown to encase the hydrophobic substituents on the north and south ends of the inhibitors (i.e., phenyl, benzimidazole, or carboxamide substituents) through van der Waals interactions (Figure 4c). Notably, all of the analogs were shown to disrupt the hydrogen-bond network upon the formation of transient hydrogen bonds between their amide group and residues of the proton relay channel (Figure 4b). Additionally, the S302A substitution was shown to impact this network of hydrogen bonds as well as the contribution of hydrophobic residues to analog binding. This mutation was shown to expand the binding pocket, diffuse the hydrogen-bond network, and selectively impact the binding of HC2091 analogs but not HC2099 and mixed analogs (Figure 4d).

The V299A amino-acid substitution, which causes pan-resistance to all three scaffolds (Figure 2b), was established as another crucial residue in the binding pocket. The V299A substitution drives a reduction in the pocket size through altering the aforementioned hydrogen-bond network. Residue decomposition analysis (Table S7) indicates that the contribution of hydrophilic residues in the pocket decreases for most ligands as a result of the pocket size reduction, impacting the affinity toward the ligands.

The M313R substitution, which also causes pan-resistance to the three scaffolds, was shown not to directly interact with the ligands but to alter intramolecular interactions with the target. The mutated residue was shown to form charge-assisted hydrogen bonds with the backbone of nearby residues, leading to several structural perturbations of the target and changes in pocket solvent accessibility. This seems to impact the binding of most of the simulated ligands, except MSU-45683 (Figure 4c,d). While the presence of background mutations could present a limitation to correlations between the biological cross-resistance patterns (Figure 2b) and the computationally predicted patterns (Figure 4d), correlation was established through linear regression (Figure 4e), showing a strong association (*R* ^2^ = 0.7655, *p* < 0.0001). In this manner, the combined interactions of the isolated amino-acid substitutions and the analogs provide structural insights into features driving resistance and susceptibility to MmpL3 inhibitors.

### The MmpL3 Inhibitor Panel Exhibits Varying Efficacy against Multidrug-Resistant Clinical Isolates

To establish the spectrum of activity against drug-resistant clinical isolates, selected inhibitors were screened against a panel of 30 clinical isolates in a dose–response assay. All of the isolates except MAB019 were characterized for their resistance profiles to standard-of-care treatments. All of the characterized isolates (29 of 30) exhibit multidrug resistance to multiple classes of standard-of-care treatments for Mab (e.g., macrolides (clarithromycin), aminoglycosides (amikacin, tobramycin), tetracyclines (doxycycline, minocycline), and fluoroquinolones (ciprofloxacin, moxifloxacin)) based on the CLSI standards,^44,45^ with tigecycline being the only active agent against all characterized isolates (Table S3). Similar to the mutant cross-resistance, normalized AUCs (Table S4) were used to calculate *Z*-scores, which were clustered in Figure 5. Clinical isolates fall into three clusters based on both their AUC and EC_50_ values: a cluster resistant to all the scaffolds and their analogs (Mab004–Mab089), a scaffold-dependent resistant cluster, resistant to HC2091 analogs (Mab041-Mab057), mixed analogs (Mab 007), or HC2099 analogs (Mab086), and a cluster sensitive to all the scaffolds (Mab048-Mab006 and Mab019-Mab088). The observed resistance and sensitivity patterns did not correlate with parameters such as the cell morphotypes (rough vs smooth), extent of antibiotic resistance, or the phylogeny of the isolates. Additionally, variant calling in the *mmpL3* region of the isolates revealed no correlations between those harboring mutations (Table S3) and their patterns of resistance, suggesting alternative correlations that can be uncovered by conducting a genome-wide association study, linking genetic variations to the observed phenotypes.

**Figure 5:**
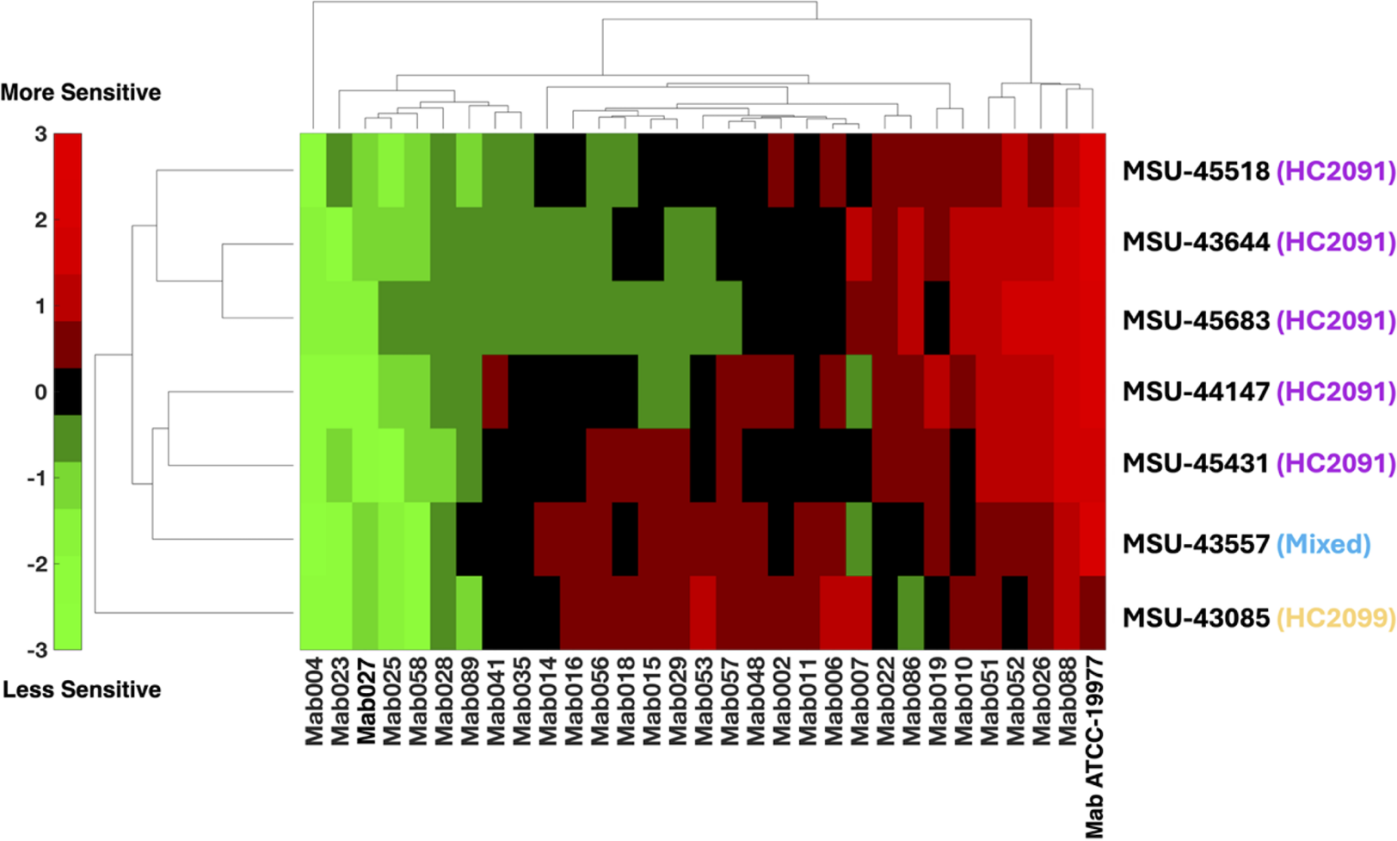
Multidrug-resistant clinical isolates have varying levels of sensitivity and resistance to MmpL3 inhibitors. Analogs and isolates are clustered into different clades, reflecting the potential of the different scaffolds in clinical contexts and hinting at the underlying genomic–phenotypic associations. The color scale is based on the *Z*-scores of the treated groups, normalized to the treated reference strain (ATCC 19977), where green indicates less activity (i.e., more resistance) and red indicates more activity (i.e., less resistance) compared to the reference. The dendrogram on the left side reflects the clustering pattern of the analogs based on their structural similarities, while that on top reflects the similarities of the resistance/sensitivity profiles of the clinical isolates which can be attributed to common genomic signatures that can be studied via a genome-wide association study (GWAS). *n* = 2, *m* = 3.

### Pairwise Combination Studies Reveal Complex Drug Combination Interactions

Multidrug regimens are the basis for antimycobacterial therapy; therefore, it is important to define drug interaction profiles with the standard-of-care treatments (i.e., additivity, synergism, or antagonism).^2,46–48^ Notably, MmpL3 inhibitors are predicted to synergize with some antibiotics as their activity will weaken the permeability barrier of the mycomembrane. Indeed, in Mtb, the HC2091 and HC2099 MmpL3 inhibitors exhibited strong synergy with rifampin.^31^ We assessed the pairwise drug interaction profiles of the MmpL3 inhibitor panel with other test compounds, standard-of-care treatments, and relevant drugs (Table S5) using the diagonal measurement of n-way drug interaction (DiaMOND) assay.^31,49^

For the DiaMOND assay, the fractional inhibitory concentration of a two-drug combination (i.e., FIC_2_) was the metric used to evaluate the interaction. Drug interactions are defined in terms of additivity: 0.8 < FIC_2_ < 1.2, synergism: FIC_2_< 0.8, and antagonism: FIC_2_ > 1.2. A matrix of FIC_2_ values was generated for every drug combination (Table S6), and a hierarchical cluster analysis algorithm was used to create the DiaMOND clustergram in Figure 6. The MmpL3 panel demonstrates a favorable drug–interaction profile that is analog-dependent, displaying additivity or marginal synergism with linezolid, ethambutol, and rifampicin (FIC_2_ of 0.67–1.13). Notably, rifampicin is characterized as inactive in Mab due to the drug-modifying activity of the ADP-ribosyl transferase Arr_MAB_,^50^ and we later report in this study a potential mechanism of the inhibitor-dependent sensitization of Mab to rifampicin. Many of the MmpL3 inhibitors also synergize with ciprofloxacin, bedaquiline, amikacin, clofazimine, rifabutin, meropenem, and clarithromycin, with a few analogs displaying additive interactions (FIC_2_ of 0.53–1.05). The MmpL3 inhibitors mostly exhibit additivity with HC2210 (FIC_2_ = 0.88–0.97), a nitroreductase-dependent nitrofuran that we previously described,^51,52^ with MSU-45431 showing synergism (FIC_2_ = 0.42). Notably, the only antagonism observed with an MmpL3 inhibitor is that with the aminoglycoside kanamycin (FIC_2_ = 1.46–2.27), to which Mab confers resistance via multiple aminoglycoside kinases and acetyl and methyl transferases.^13,14^ Amikacin, the other aminoglycoside in the study, is less susceptible to enzyme-dependent deactivation,^53^ and thus, it demonstrates a different interaction profile than kanamycin.

**Figure 6:**
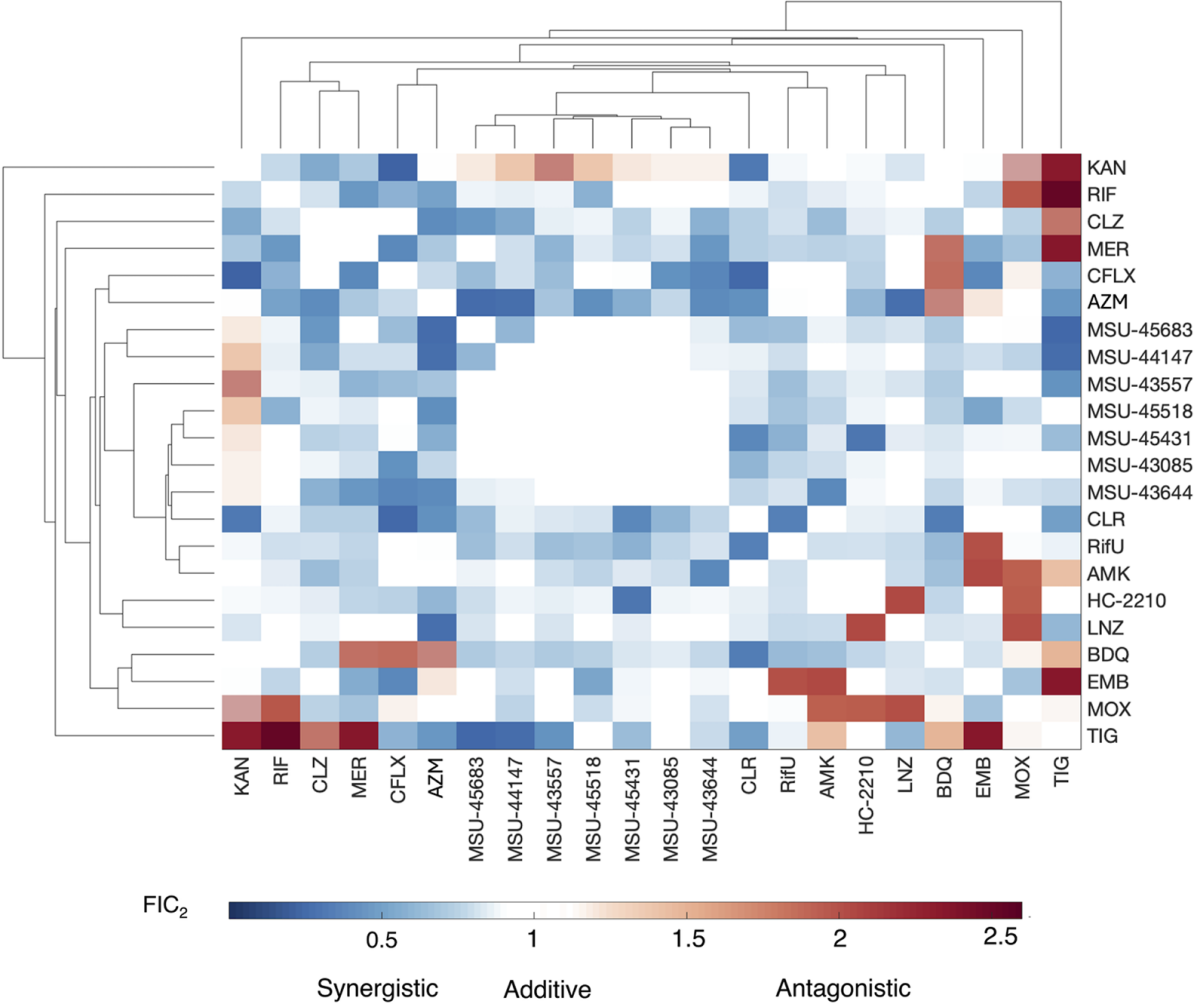
Drug interaction studies via DiaMOND reveal complex interaction profiles between different classes of antimycobacterial drugs, lead analogs, and relevant drugs. Pairwise interactions of all combinations of the 20 different compounds (See Table S5), where hierarchical cluster analysis of the FIC_2_ matrix (see Table S6) identified additive (FIC_2_ of 0.8 to 1.2), synergistic (FIC_2_ < 0.8), and antagonistic (FIC_2_ > 1.2) interactions between the different combinations. *n* ≥ 2, *m* = 3. KAN: Kanamycin, RIF: rifampicin, CLZ: clofazimine, MER: meropenem, CFLX: ciprofloxacin, AZM: azithromycin, CLR: clarithromycin, RifU: rifabutin, AMK: amikacin, LNZ: linezolid, BDQ: bedaquiline, EMB: ethambutol, MOX: moxifloxacin, TIG: tigecycline.

Consistent with previous reports, moxifloxacin demonstrates varying degrees of antagonism with several treatments (e.g., kanamycin, bedaquiline,^54^ ciprofloxacin, linezolid, and amikacin (FIC_2_ = 2.16–2.47)).^49,55^ Additionally, ethambutol exhibits antagonism in combination with amikacin and rifabutin (FIC_2_ = 2.5 and 2.47, respectively).^49,55^ Bedaquiline was also shown to antagonize meropenem (FIC_2_ = 2.33), consistent with previous findings that it attenuates the efficacy of β-lactams,^56,57^ and ciprofloxacin.^54^ Contrary to some studies, however, the combination of moxifloxacin or amikacin with clarithromycin was found to exhibit additivity (FIC_2_ = 1.02 and 1, respectively) instead of antagonism,^58,59^ in accordance with more recent studies.^55^ Additionally, significant synergism was observed for ciprofloxacin with both kanamycin and clarithromycin (FIC_2_ of 0.29 and 0.33, respectively), and rifabutin with clarithromycin (FIC_2_ = 0.44), as previously reported.^55^

### The Transcriptomic Signature of MmpL3 Inhibitors Reflects Their Bactericidal Mode of Action

To assess the metabolic and biochemical consequences of MmpL3 inhibition, we conducted a transcriptomic analysis of Mab treated with 5 times the MIC of MSU-43085 or MSU-45683, relative to a DMSO-treated control. Differential expression in 1809 and 2033 genes was observed for MSU-43085 and MSU-45683, respectively (Data set 1). Of those, 1258 were similarly altered by both treatments, representing the Mab transcriptomic signature under MmpL3 inhibition (Figure S13).^60^ Ortholog search and clustering were conducted using *M. tuberculosis* H37Rv genome as a reference using orthovenn3 ^61^ (GO term enrichment significance *p* < 0.05), and secondary pathway enrichment was conducted using Kyoto Encyclopedia of Genes and Genomes (KEGG).^62^ Data was then categorized by pathway while considering differential expression to summarize the genetic, molecular, and metabolic responses to the treatments (Figures S15 and S16), and key pathways are presented in Figure S14.

Since the MmpL3 inhibitors target mycolic acid (MA) utilization, it is necessary to focus on the mycolic acid synthesis machinery. Under MmpL3 inhibition for 24 h in vitro, the genes involved in the synthesis or functionalization of MA are downregulated as reported in Mtb (*inhA, fabG/H, acpM/S, pks13, kasA, fabD, fadD32, accD4/5, umaA1, far, accA3, fasI*, *desA2*, and *mabA*), while *desA1* is upregulated, contrary to observations in Mtb.^32,63–66^ Notably, the MAB_2027-MAB-2038 locus is upregulated, where these genes were linked to long-chain MA synthesis (Figure S14),^67,68^ suggesting either it is a compensatory mechanism involving alternative lipids to rescue growth under MmpL3 inhibition or it plays a role in synthesizing a membrane lipid other than MA since it has its own *mmpL/S* genes and a complete lipid synthetic machinery.

Additionally, markers associated with bactericidal activity and metabolic stress are observed. These include downregulation of DNA replication/repair and translational machinery, oxidative phosphorylation,^69^ de novo synthesis of several lipids,^70,71^ and steroid metabolism.^63,67,72–76^ Various stress responses include strong upregulation of alternative σ-factors (*sigB, sigH, sigD, sigE*, and *sigI*), the *iniBAC* operon, heat-shock proteins, and DNA-chaperones (*htpX, hsp, hsp31, hsp20, hspR, dnaJ*, and *dnaK*), indicating general, oxidative, and envelope stresses, consistent with the mode of action of the MmpL3 inhibitors and previous reports in Mtb (Figure S14).^32,66,73,74^ Several members of the two-component regulatory systems (*senX-regX, mprA*, and *prrAB*) are upregulated, while others (*mtrAB*, *kdpDE*, and *narL/S*: MAB_4519c/MAB_4520c) are downregulated.^63,73,75–77^ Several WhiB transcriptional regulators are differentially expressed. Examples include *whiB2*, *whiB7, and whiB1,* involved in cell cycle regulation, drug-resistance behavior of Mab, and redox balance.^78–81^

The mammalian cell entry (MCE) genes, associated with lipid transfer and biofilm formation, are among the most downregulated genes.^82,83^ All annotated MCE loci are either not changed or downregulated, except for the locus downstream of the *mmpL3* gene (MAB_4508), MAB_4511c-MAB_4518c, indicating a putative role under MmpL3 inhibition (Figure S14). There is also downregulation of ATP-binding cassette (ABC) permeases involved in nutrient acquisition, such as amino acids and monosaccharides, while ABC permeases for ribonucleotides, several ions, and metals are upregulated. Multidrug ABC permeases such as MAB_2176, MAB_2177, MAB_2178, and *mk1* are all upregulated.^84–86^ Also, several MmpL/S systems are differentially expressed, which are associated with multiple pathways in other mycobacteria, including glycolipid and siderophore transport, and drug efflux (Figure S15i).^64–66^

MmpL3 inhibition results in upregulation of drug-resistance genes for several drug classes, including macrolides (*erm41*) and aminoglycosides (*acc1, acc2, acc2′* , and *eis2*), which are known to be driven by *whiB7*.^59,86^ This may explain the antagonism that kanamycin exhibits in combination with MmpL3 inhibitors. However, among their respective classes, aminoglycoside amikacin and macrolides azithromycin and clarithromycin exhibit relatively low susceptibility to enzymatic inactivation, which underlies their inclusion as core agents in most therapeutic regimens. Notably, they retain synergistic activity with MmpL3 inhibitors despite the induction of drug-modifying enzymes targeting aminoglycosides and macrolides in response to MmpL3 inhibition. However, *arrMab*, an ADP-ribosyl transferase involved in resistance to rifampicin, was downregulated, suggesting the MmpL3-associated downregulation of the gene as a putative mechanism of the synergy with rifampicin observed in the DiaMOND assay.

## Discussion

MmpL3 is a promising drug target in *M. tuberculosis* and NTMs. However, the only MmpL3 inhibitor to reach clinical trials is SQ109 for the treatment of *M. tuberculosis*. Notably, SQ109 is inactive in several NTMs, including Mab. Here, we present evidence supporting continued study of the HC2091, HC2099, and merged series analogs as potential new treatments for Mab infection. Several lines of evidence support the proposed mechanism of action of the presented analogs (i.e., inhibition of MmpL3). In a competitive binding assay, early analogs (HC2091 and HC2099) were shown to directly bind to MmpL3 of *M. tuberculosis* and displace the fluorescent probe North 114 bound to it in a dose-dependent manner.^31^ Additionally, in this study, the analysis of isolated lipids revealed a reduction of TDM under MmpL3 interference relative to the vehicle control, which, when added to their very narrow spectrum of activity and the selection of multiple independent mutations in the *mmpL3* gene (MAB_4508) conferring resistance to the analogs, demonstrates that targeting and inhibiting MmpL3 is the most probable mechanism of action of the analogs.

The MmpL3 inhibitors demonstrate favorable antimycobacterial properties, including high potency and time-dependent bactericidal activity, low cytotoxicity, efficacy in disrupting biofilms and reducing the viability of Mab inside the biofilms, low frequency of resistance, and putative intracellular efficacy in macrophages, warranting further development as new therapeutics for NTMs. Notably, the very narrow spectrum of activity, which is exclusive to mycobacteria, reflects the exclusivity of the targeted mechanism involving TMM transport by the MmpL3. Also, the inhibitors exhibit favorable pairwise interaction profiles with the standard-of-care treatments in the DiaMOND assay in the form of in vitro synergism with linezolid, ethambutol, rifampicin, ciprofloxacin, bedaquiline, amikacin, clofazimine, rifabutin, meropenem, and clarithromycin and additivity/synergism with moxifloxacin depending on the analog. Also, the efficacy of the panel is not affected by drug efflux, which is a common mechanism of resistance to several drug classes. Figure S16 shows that there is no significant difference between the efficacy of the analogs alone and in combination with verapamil, an inhibitor of efflux. This establishes the potential clinical value of regimens including an MmpL3 inhibitor with either amikacin and clarithromycin or amikacin and rifabutin as first-line treatment with clofazimine and bedaquiline potentially added in the step-down phase of treatment. The compounds are effective when used in combination with standard-of-care antibiotics and are active against several drug-resistant clinical strains. Future studies will need to be conducted to further define the drug-like properties of the compounds, including establishing efficacy against Mab in animal models of infection.

Comparing differences in compound structures and resistance conferred by specific MmpL3 amino-acid substitutions provides an opportunity to define specific protein–ligand structure associations with resistance and susceptibility. In a forward genetic selection, 16 different mutations were isolated, conferring resistance to MmpL3 inhibitors. The MmpL3 amino-acid substitutions, insertion, or deletion mutants span the six transmembrane helices (TM1–TM6), with one in a loop (L6). The mutations map to specific structural features, with one substitution in TM4 (Y266F), seven in TM5 (V299A, S302T/A, I306S/T, M313R, and MPML X2 (311–314)), one in TM6 (A339E), four residues in L6 (ΔV349–R352), two in TM8 (V547R/G and L551S/F), one in TM9 (L568I), and one in TM11 (T655I). Eleven of the substitutions line the binding pocket, suggesting that the resistance is driven by disruption of ligand–protein interactions. Of these, only V299A, S302A, I306S, V547G, and L551S/F were reported in the literature, and the rest we believe are novel to this study.^87,88^ Notably, the only large-scale mutations (i.e., insertions/deletions) previously reported were a deletion in I552 and M313-L314.^30^ Here, we report two additional mutations: a duplication in the same region of the two-amino-acid deletion, MPML (311–314) X2, and a total deletion of loop 6 (V349-R352), which, despite being distant from the binding pocket, are pan-resistant to all three scaffolds (clade A), reflecting the impact of the structural perturbations they impose on the pocket. Another interesting mutation is Y266F, which is one of the conserved tyrosines involved in proton relay. Similar mutations were isolated in Mtb, including Y252C/S,^87^ suggesting that an electron density (i.e., the phenyl of the phenylalanine, thiol of cysteine, or hydroxyl of serine) is sufficient, but not optimal, for the proton relay and coupling to TMM transfer.

Cross-resistance profiling demonstrates that analog and mutation clustering are dependent on the SAR of the former and the impact of the latter on analog binding. Analogs cluster into three clades representing the three different series, with intraseries differences suggesting different interactions between their substituents and residues. For example, in the mixed series, MSU-43557 and MSU-45606, which, unlike the rest of the mixed analogs, share the same smaller substitutions on the benzimidazole side (sites A and B), have similar resistance profiles with S302A, suggesting a common putative interaction that is lost in the mutant but retained by bulkier substitutions on the other analogs. Another example is M313R, which impacts HC2091 analogs except MSU-45431, MSU-45518, and MSU-45683, which share a bulky group (CF_3_/ethyl) on the carboxamide side that is smaller in the other HC2091 analogs (methyl), suggesting the impact of substitutions at site D in anchoring the molecules in the pocket despite the disruptive perturbations introduced by the mutation. A similar observation can be deduced for MSU-43186 (an HC2099 analog with a bulky azepane on the carboxamide) in the case of S302T and Y266F, which are on the same plane on different sides in the pocket.

Similar clustering patterns have been observed for the analog subset used in clinical isolate cross-resistance (Figure 5), reflecting the impact of the SAR of the analogs on their antimycobacterial activity. However, less is understood about the drivers of the clustering patterns of the isolates as no associations with morphology, extent of drug resistance, or phylogeny were observed. A similar observation was recorded when the activity of HC2210, a nitro-containing compound we previously characterized, was studied in the same set of clinical isolates, showing a similar lack of associations but a different pattern of sensitivity and resistance than MmpL3 inhibitors.^52^ This suggests that their resistances are not driven by generic intrinsic mechanisms (e.g., membrane permeability) but rather specific mechanisms relevant to their modes of action. Genome alignments between the ATCC 19977 and variant calling revealed no associations with variations in the *mmpL3* region as well, but the high variability among the isolates, demonstrated in Figure S18, presents several potential associations to be investigated. Further genetic and genome-wide association (GWAS) studies are needed to understand the mechanisms driving sensitivity and resistance patterns. This is especially crucial to understand the limited efficacy of the analogs against one cluster of isolates (Mab004–Mab089, Figure 5).

Moreover, the recorded limited efficacy of MmpL3 inhibitors in this cluster of clinical isolates underscores the major clinical challenges in the treatment of *M. abscessus* compared to the rest of the mycobacterial infections. As shown in Table S3, except for tigecycline, not a single standard-of-care treatment, including the first-line drugs clarithromycin and amikacin, is effective against all of the clinical isolates, owing to several mechanisms of intrinsic resistance (e.g., drug impermeability, efflux, and inducing biofilms) or acquired resistance (i.e., a mutation in the target). For the MmpL3 inhibitors, no target mutations were found driving the observed resistance in the isolates Mab004–Mab089, suggesting either a broader acquired resistance mechanism or a shared intrinsic resistance mechanism in these isolates. While this challenges the clinical potential of the inhibitors on the surface, the fact that not even the first-line standard-of-care drugs retain efficacy against all isolates emphasizes the clinical challenges of Mab treatment and reiterates the clinical potential of MmpL3 inhibitors.

Associations between amino-acid substitutions, in terms of impact on normal function and strain fitness, were also observed. In general, the closer the mutation is to the TMM pocket, which lies between TM7–10,^22^ and the more disruptive it is to the shape, size, and electrostatic or van der Waals maps of the pocket (i.e., residue polarity and hydrophobicity), the more impact it has on the normal protein function and hence the fitness of the mutant. Perturbations in pocket size (e.g., MPML x2, S302T, and L551F), polarity (I306S/T, L551S, and S302A), or both (M313R and V547R) were observed, and all putatively impacted the fitness proportionally to the level of disruption. Less disruptive mutations that are distant from the TMM pocket (i.e., L568I and T665I) had a smaller impact on fitness. Potential changes in the proton relay capacity associated with Y266F can explain the putative fitness defect observed for this mutant.

Molecular dynamics simulations of wild-type or mutant MmpL3 with the different classes of inhibitors reveal putative underlying mechanisms of several structure–function associations discussed above. The overarching mechanism of resistance conferred by a specific mutation involves structural perturbations induced by the mutation, which change the pocket size as well as its electrostatic/hydrophobic maps. This changes the exposure of the pocket to external solvent (i.e., surrounding water molecules), resulting in the solvation of the ligand and formation of transient solvent–ligand interactions at the expense of ligand–pocket interactions and affinity. Unless the ligand can compensate for the solvent-driven loss of interactions with the pocket in the mutant, the mutation would render the ligand less effective against this mutant. In addition to solvent accessibility, a change in pocket size also alters the ligand–pocket interactions and, thus, the residue contribution to binding energy, independent of the solvent.

Three amino-acid substitutions were presented in light of this proposed mechanism: S302A, V299A, and M313R, each altering the aforementioned parameters in a specific way. For S302A, the introduction of a hydrophobic side chain slightly diffuses the hydrogen-bond network that stabilizes the proton-relay channel and the central vestibule of MmpL3. It also alters the size of the ILE-rich hydrophobic subpocket in the upper section of the pocket (Figure 4c). This increases solvent accessibility, exposing ligands to solvent molecules and stripping the interactions from pocket residues. However, in the presence of the S302A mutation, HC2099 and mixed analogs engage the surrounding hydrophilic residues through hydrogen bonding with their benzimidazole nitrogen, potentially compensating for the solvent-driven loss of interactions. For HC2091 analogs, the absence of additional interactions under the pocket expansion, and the loss of van der Waals interactions between the hydrophobic subpocket and the Cl/CF_3_ substitutions of their phenyl, due to the solvation of the ligand, makes this mutation selectively resistant to HC2091 analogs, reflecting the cross-resistance profile in Figure 2b. For the V299A mutation, the pocket size is reduced and the hydrogen-bond network is more compact, altering the ligand accessibility to the pocket and reducing the residue contribution of the hydrophilic residues in or near the proton relay channel to the binding. As a result, the hydrogen bond between the ligand amide and the proton relay channel, a key interaction for all 3 scaffolds, becomes more transient, weakening binding of all analogs and leading to pan-resistance. An exception to this resistance profile is the HC2091 analogs MSU-45431 and MSU-45683, which are small enough to access the altered pocket and leverage additional van der Waals interactions to anchor the ligands in the pocket, leading to sensitivity against the V299A mutant.

For the M313R mutation, even though it does not directly interact with the ligands, the charge-assisted hydrogen bonding with the backbones of several pocket-lining residues expands the pocket, increasing the ligand solvation and loss of ligand–pocket interactions. This renders analogs of the three series less effective against the mutant, except the HC2091 analogs MSU-45431, which maintains the same activity as the wild-type, and MSU-45518 and MSU-45683, which have better sensitivity. The presence of bulkier hydrophobic substituents on the carboxamide end (i.e., ethyl or CF_3_) offers a justification for this exception, as they anchor the molecules in the pocket and compensate for the loss of interactions by making more interactions with the pocket (i.e., van der Waals or hydrogen bonding).

Notably, bridging the resistance-driving mutations with their consequent putative fitness defects further reflects the potential of the presented MmpL3 inhibitors. In this study, the analogs were leveraged not only as candidate therapeutics but also as probes to study the protein structure of MmpL3 and direct the process of evolution-driven drug discovery in Mab. We hypothesize that specific analogs may bias selection for specific amino-acid substitutions that confer resistance while leading to a fitness defect. By analyzing patterns of sensitivity and resistance alongside mutant fitness assessments, we can prioritize the most effective analogs against the majority of mutants. At the same time, we account for the inevitable emergence of resistance by favoring analogs that select for mutants with the greatest fitness disadvantage. Mutations in M311–L314 residues, isolated against MSU-44147 (HC2091 analog) and MSU-43557 (mixed analog) (Table S1), best exemplify this association. All mutations in this region of the protein lead to the greatest fitness defect while conferring resistance to all three series (Figure 2b). Thus, clinical emergence of these mutations under MSU-44147 or MSU-43557 treatment would compromise Mab growth and infection capacity. This presents an extra advantage in the context of multidrug therapeutic regimens since mutations conferring resistance to an MmpL3 inhibitor while leading to fitness defect were shown to be more sensitive to other treatments commonly used in the regimens, especially those targeting the cell envelope, such as meropenem (Figure S12). Both this strategy and the experimental framework we present here support the potential of evolution-driven drug discovery to slow the emergence of drug resistance and generate more durable drugs.

MmpL3 inhibition has been associated with a state of oxidative, metabolic, and osmotic stress in Mtb.^72,73^ In this study, we report a similar transcriptomic signature in Mab with minor differences reflecting the differences between the two species. A global downregulation of central metabolism and oxidative phosphorylation, replication and transcription machinery, and permeases/transferases is observed, potentially to mitigate metabolic and osmotic stress. Exceptions include the pentose-phosphate pathway, which is necessary for synthesizing the sugar moieties of various membrane lipids, arabinogalactans, and peptidoglycans.^89,90^ A global upregulation in pathways involved in redox and osmotic balance, envelope stress, and drug resistance is observed. However, less is understood about the MCE locus downstream of the MmpL3 gene (MAB_4511c-MAB_4518c) and the putative mycolic acid synthesis locus (MAB_2027-MAB-2038), which are upregulated, and their role in mycolic acid synthesis and transfer and virulence. A combination of genetic, biochemical, and metabolic in vitro and in vivo experiments can explain the functional aspects of differential expression in these loci/pathways under MmpL3 inhibition in Mab.

### Limitations of the Study

While multiple mutants isolated against the MmpL3 inhibitor panel harbor an MmpL3 mutation in an isogenic background to the wild type, several other mutants carry multiple background mutations (Table S1). Additionally, several mutants isolated against MSU-43085, but not other analogs, which we excluded from this study, display varying levels of resistance to the MmpL3 inhibitors in the absence of an MmpL3 mutation, suggesting the involvement of secondary resistance mechanisms and a putative secondary mechanism of action of MSU-43085 in Mab. Also, mutants carrying the same MmpL3 mutation in different genetic backgrounds displayed variable levels of fitness relative to the wild type. This presents a limitation when establishing a genotype–phenotype correlation (i.e., mutation-resistance pattern or fitness), especially because the contribution of the background mutations to the phenotype is unknown. Another limitation imposed by the presence of background mutations is the presence of outliers in the biological data that skew the linear regression between the computational and biological parameters (Figure 4), yielding a correlation coefficient of 0.7655. To address this limitation, a conditional MmpL3 knockout mutant needs to be generated,^91,92^ in which mutant alleles are complemented back, to compare the impact of the mutant alleles in isogenic backgrounds. Nevertheless, for several MmpL3 mutants (Table S1), there appear to be few or no unlinked mutations, allowing us to ascribe the likely phenotype (i.e., resistance/fitness defects) to MmpL3 mutations. Additionally, since the findings of this study were undertaken using the *M. abscessus* ATCC 19977, more consideration should be given to the heterogeneity among Mab isolates for the findings to be generalizable.

### Concluding Remarks

In this study, we introduce several representative analogs belonging to three series of MmpL3 inhibitors (i.e., HC2091, HC2099, and mixed series). These analogs are presented as both potential therapeutics and experimental probes to establish structure–function associations regarding MmpL3 function, sensitivity, and resistance to inhibitors and influence on bacterial fitness. Characterization of the analogs’ mode of action indicates their favorable antimycobacterial properties (i.e., comparable efficacy in vitro, in biofilms, and in macrophages) to the standard-of-care treatments, efficacy against multidrug-resistant clinical isolates, low frequency of resistance, and positive pairwise interaction profiles with standard-of-care treatments). Cross-resistance studies and fitness assessment of the *mmpL3* mutants emphasize several associations between structural aspects of MmpL3 and the inhibitors and functional aspects concerning their sensitivity and resistance profiles and fitness defects linked to particular mutations. This experimental framework, coupled with computational modeling and molecular dynamics simulations, enforces the structure–function associations and their potential in directing the evolution-driven discovery of more durable drugs to be used in combination with current regimens.

## Materials and Methods

### Primary Cell Cultures

Bone marrow-derived macrophages (BMMΦ) were isolated as described by Johnson et al.^93^ C57Bl/6 mice were euthanized by carbon dioxide asphyxiation followed by cervical dislocation, and the ilia, femurs, and tibias were sterilely removed to isolate the bone marrow. Bone marrow-derived macrophages (BMMΦ) were cultured in Dulbecco’s Modified Eagle medium (DMEM) supplemented with 1% sodium pyruvate (v/v), 1% l-glutamine (v/v), 10% heat-inactivated fetal bovine serum (FBS) (v/v), 1% penicillin/streptomycin (10,000 IU/mL each) (v/v), and 10% L-cell (L-929) conditioned media and incubated for 5–7 days at 37 °C under 5% CO_2_. Only P0 and P1 cells were used for the cytotoxicity and intracellular killing assays.

### Bacterial Strains and Dose–Response Assays

Mab was grown to an optical density of 0.5 to 1.0 at 600 nm (mid log phase) in a shaking incubator, and cultures were diluted to an OD_600_ of 0.1 and aliquoted into clear-bottom black 96-well plates (198 μL/well). Compounds to be tested, including MmpL3 inhibitor analogs and standard-of-care treatments, were prepared by dissolving fresh powders in a solvent (DMSO or water) and serially diluting in the same solvent, starting from 8 mM to 8 nM (16-point serial dilutions, 2.5-fold steps). For compounds known to be weakly active in Mab (e.g., rifampin, meropenem, and ethambutol), a starting concentration of 16 mM was used. Amikacin was used as the positive control at 3 mM, and blank solvents were used as the negative control. Treatments and controls were added to culture plates (2 μL/well, 1:100 dilution), creating a dose range between 80 and 0.08 nM for the tested treatments and 30 μM for the positive control. The plates were incubated for 3 days at 37 °C in zip-lock bags with moist paper towels to maintain the humidity, after which OD_600_ values were taken using a PerkinElmer Envision plate reader. OD values were used to calculate the percentage of growth inhibition using the amikacin and DMSO controls as the 0% and 100% references, respectively, and dose–response curves were constructed using the variable slope, four-parameter nonlinear regression model in Prism 10 software. Each compound was tested in three technical replicates, and in vitro treatment efficacy was determined based on the EC_50_ and Minimal Inhibitory Concentration (MIC), where the latter was calculated using the former and the hillslopes of the dose–response curves. To assess the spectrum of activity of the 7 MmpL3 inhibitors, a dose–response assay was conducted using the strains previously mentioned (Table S8). Mycobacterial strains were grown in Middlebrook 7H9 medium (BD Difco) supplemented with 10% OADC (oleic acid, albumin, dextrose, and catalase) (v/v), and 0.05% Tween 80 (v/v), while nonmycobacterial strains were grown in LB medium (Sigma-Aldrich), except *Enterococcus faecalis*, in Brain-Heart Infusion broth (Sigma-Aldrich). Tobramycin was used as a positive control for *P. aeruginosa*, amikacin for *M. abscessus*, and rifampicin for *M. tuberculosis* and kanamycin for the remaining strains.

### Kinetic Killing Assay

Mab was grown as previously described, and cultures were diluted to an OD of 0.2 and seeded in T25 tissue culture flasks (15 mL each). In triplicate, flasks were treated with 5× the MIC of the designated treatment: MmpL3 inhibitors (**MSU-43085**, **MSU-43644**, **MSU-44147**, **MSU-43557**, **MSU-45518**, **MSU-45431**, and **MSU-45683**), and controls (DMSO, amikacin, and bedaquiline) (at 20 and 5 μM final concentration, respectively), were used as the negative control and the positive controls. Flasks were placed in a latch box with moist paper towels and incubated at 37 °C. Samples were taken from the flasks at 3, 6, 12, and 24 h and then every 12 h until day 5, serially diluted seven times, 10-fold each, in 1× phosphate-buffered saline (PBS) with 0.15% Tween 80 (v/v) in 96-well plates, and plated on 7H10 quadrant plates with OADC (10% v/v) and cycloheximide (1% v/v). Plates were incubated in zip-lock bags at 37 °C for 3–4 days, and colonies were counted to estimate bacterial survival and time-dependent killing. Experiments were repeated twice, and all compounds were tested in triplicate.

### Intracellular Killing Efficacy and Analog Cytotoxicity

Studies were carried out as previously described.^31,32,93^ Mab (ATCC 19977) expressing mEmerald GFP (pmV261 hsp60’::mEmerald) was grown in 7H9 supplemented with zeocin (Thermo Scientific) at 5 μg/mL to an OD_600_ of 1 and resuspended in prewarmed uptake buffer at a final concentration of 9.2 × 10^8^ cfus/mL. Primary bone marrow-derived macrophages (BMMΦ) were isolated, pelleted, and resuspended to a final cell concentration of 2 × 10^6^ cells/mL. Cells were seeded the day before the infection in tissue culture grade 96-well plates and infected with Mab at an MOI of 6. Infected macrophages were incubated at 37 °C and 5% CO_2_ for 3 h to allow bacterial uptake, after which the bacterial suspension was removed, and the cells were washed three times, treated with 20 μM of amikacin, and incubated for 3 h to kill extracellular bacteria. Following another washing step, BMMΦ were treated with MmpL3 inhibitors, clarithromycin, and rifabutin at 30 μM as the positive controls and DMSO as the negative control and incubated at 37 °C and 5% CO_2_ for 3 days. The bactericidal activity of the inhibitors was assessed using the dose–response curves (GFP intensity (excitation at 495 nm and emission at 509 nm) versus inhibitor concentration) constructed by Prism 10 (Figure S3). Eukaryotic cytotoxicity was determined as previously described by treating BMMΦ with the MmpL3 inhibitors (200 to 0.13 μM), the positive control, 4% Triton X-100, and the negative control, DMSO. Cells were incubated for 3 days at 37 °C and 5% CO_2_, and cytotoxicity was assessed using the Cell Titer-Glo assay kit. Treatments were tested in triplicate, and intracellular killing experiments were conducted twice, while cytotoxicity was assessed once. A limitation of this assay is that some extracellular bacteria may exist following wash steps, AMK treatment, or macrophage cell death, and some of the observed inhibition may include the killing of extracellular bacteria. Additionally, given the long half-life of GFP (i.e., the presence of a readout even in the absence of viable bacteria), these results demonstrate killing of Mab in macrophages, but the dynamic range of killing may differ from CFU-based assays.

### Total Lipid Extraction and Thin-Layer Chromatography of Mab

Lipid extraction and TLC were performed as previously described with slight modifications.^31,32^ Mab culture (OD_600_ of 0.6) was divided into 40 mL portions in T75 flasks, treated with 5× the MIC of the panel of MmpL3 inhibitors and DMSO as a negative control, and incubated for 24 h. A 10 mL sample was taken from each flask at 24 h to perform lipid extraction and TLC analysis. Lipids were extracted using a mixture of chloroform:methanol (2:1), dried in a nitrogen bath, and resuspended in 3 mL of the same mixture. To remove water-soluble impurities, 0.5 mL of water was added, the samples were centrifuged, and the organic phase was separated, dried in a nitrogen bath, and resuspended in 0.5 mL of the mixture. Samples were spotted on an HPTLC silica gel 60 plate (EMD Chemicals Inc.), and TLC was performed by using a 24:1:0.5 chloroform/methanol/H_2_O solvent system. To visualize the TLC, plates were sprayed with phosphomolybdic acid (5%, w/v, in ethanol) and charred with a heat gun.

### Membrane Potential Assay

The membrane potential (ΔΨ) assay was performed as previously described^31,94^ with slight modifications. Mab was grown to the mid log phase (OD_600_ of 0.5 to 1.0), and 10 mL of the culture was centrifuged at 4000*g* and resuspended in 1 mL of 7H9 medium. Cells were labeled with 60 μM DiOC_2_ (Thermo Scientific) and incubated at 37 °C for 30 min. Cells were washed twice, suspended in 7H9 medium to an OD_600_ of 0.2, and aliquoted in 96-well clear-bottom plates (198 μL/well). Cells were treated in triplicate with the 7 MmpL3 inhibitors at different concentrations (160, 40, 10, and 1 μM), the positive control protonophore CCCP (Sigma-Aldrich) at 30 μM, and the negative control DMSO. The kinetics of fluorescence (the ratio of the red/green fluorescence intensities) was measured every 2 min for 60 min (excitation at 485 nm and two emissions at 610 and 515 nm) over the 60 min reading period. The experiment was repeated twice to validate the results, and data were represented as geometric means with error bars representing the standard deviations from the mean (Figure S5).

### Nonreplicative Killing Assay

The nonreplicative killing assay was conducted as described by Berube et al. and Betts et al.^39,40,95^ To establish nutrient starvation conditions, Mab cultures grown to an OD of 1 were pelleted, washed twice with PBS, and then resuspended in 1× PBS supplemented with tyloxapol (0.05% v/v) at an OD of 0.5. Cells were aliquoted in 96-well clear-bottom plates (200 μL/well) and incubated for 1 day at 37 °C. Treatments or controls were added on the second day after the initial incubation. Cells were treated in triplicate with the 7 MmpL3 inhibitors at different concentrations (160, 40, 10, and 1 μM), the positive control Amikacin at 30 μM, and the negative control DMSO. Plates were incubated at 37 °C for 3 days, and readouts (OD_600_ and CFU counts) were taken on both day 2 and day 3. The experiment was performed twice, and the results of one representative experiment are shown in Figure S6.

### Biofilm Assessment and In Vitro Treatment Efficacy in Biofilms

Biofilm treatment efficacy was assessed in two different methods: crystal violet assay to determine the biomass of the biofilms and resazurin blue viability assay to assess viability, as previously described.^96,97^ Mab was grown and diluted to a final OD_600_ of 0.2, then seeded in two sets of 96-well polystyrene poly-d-lysine-coated plates (200 μL/well), one for each assay, and incubated at 37 °C for 6 days to form submerged biofilms, after which the medium was removed, the biofilms were washed gently with PBS, and new medium was added. The biofilms were treated with serially diluted MmpL3 inhibitors (16-point dilution, 160 μM to 0.18 nM), the positive control amikacin, at 80 μM, and the negative control DMSO and incubated for 3 days. Similarly, several standard-of-care treatments, e.g., amikacin, bedaquiline, clarithromycin, and tigecycline, were also tested in both assays in 16-point dilutions (80 μM to 0.08 nM). Treatments were tested in triplicate, and assays were performed three times (Figure S7).

### Mutant Isolation, Sequencing, and Frequency of Resistance

The isolation and confirmation of resistant mutants were done as previously described with slight modifications.^31,52^ Cultures were spun down and resuspended to an OD of 1, and 1 mL (equivalent to 3 × 10^9^ cfus) was plated on 7H10 plates supplemented with OADC, cycloheximide (10% and 1% v/v, respectively), and the 7 MmpL3 inhibitors at different concentrations (1, 3, 5, 10, 50, and 100× the MIC). Plates were incubated at 37 °C for 2 weeks or until colonies started appearing. Colonies were picked from every concentration and expanded to confirm resistance, make stocks, isolate, and sequence genomic DNA as described by Jagatia and Cantillon.^98^ Illumina-based whole-genome sequencing using 150-bp reads was conducted to identify genetic variations (single-nucleotide polymorphisms (SNPs), insertions, and deletions) with reference to the wild type. The frequency of resistance of the inhibitors was estimated at a selected concentration of every inhibitor (3× the MIC) by dividing the total number of colonies appearing on a plate by the total number of plated cfus.

### Cross-Resistance Studies

Cross-resistance studies were carried out as previously described for ref 31. A panel of 16 different validated mutants (i.e., sequenced and confirmed for resistance) and 30 clinical isolates (shown in Tables S1 and S3, respectively) was treated singly with the panel of 7 MmpL3 inhibitors. For the mutant cross-resistance, 8 additional MmpL3 inhibitors (analogs of HC2099: MSU-45655, MSU-43186, MSU-45540, MSU-45516, and MSU-45538, or belonging to the mixed series MSU-45606, MSU-45819, and MSU-45350) were tested. Dose–response curves were used to calculate the Area Under the Curves (AUCs) using Prism 10 software, which were normalized by treatment to that of the WT and standardized by calculating the *Z*-scores, which were clustered in MATLAB_R2024b software by hierarchical agglomerative clustering using the default settings of the clustergram function (Euclidean distance model and average linkage clustering).

### Bacterial Fitness Assessment

Mutants were prioritized if they had an MmpL3 mutation in a genetic background identical to that of the WT (see Table S1). If none are available for a specific mutation, the mutant with the fewest background mutations was selected. The mutants and the WT were each grown independently in 10 mL of 7H9 media in T25 flasks, as previously described. Cultures were diluted to an OD_600_ of 0.1 and seeded in 96-well clear-bottom plates labeled with the time point designated to take the OD reading of the plate (6, 12, 24, 36, 48, 72, and 96 h). Plates were incubated at 37 °C, and OD readings were taken to construct a growth curve over 5 days (Figure S10). The growth curves were used to calculate the AUCs, which were normalized to that of the WT and plotted in Figure S11. The experiment was conducted 3 times, and every strain was tested in 12 technical replicas per time point.

### Molecular Dynamics Simulation and Computational Modeling

The system was established using AlphaFold3 ^99^ to obtain a 3D structure of the apo protein (UniProt ID: A0A0U0YMV6),^100^ due to the absence of crystal structures of the Mab MmpL3. The model was minimized using the AMBER10: extended Huckel theory force field, and Amber ff10 was used for the protein structure as implemented in the Molecular Operating Environment software (MOE).^101–103^ Molecular dynamics (MD) simulations of the model were carried out using an Amber 2022. The model was prepared using the tleap module implemented in Amber 2022.^104^ The Ff14SB and Tip4PEw force fields were used for the protein and solvent (water), respectively.^105,106^ A stepwise energy minimization, decreasing the restraint weight as follows: 100, 50, 10, and 0 kcal/mol/Å^2^, was performed. Incremental heating was applied using a 1 fs time step as the temperature was increased from 0 to 300 K. A 500 ps *NVT* equilibration was carried out, followed by a 500 ps *NPT* equilibration. Isotropic pressure coupling and a Langevin thermostat were used for pressure and temperature control, respectively. Finally, a 20 ns production run was applied in 1 ns time blocks. The SHAKE algorithm was used to constrain the covalent bond lengths with hydrogen atoms.^107^ The Particle Mesh Ewald method was implemented for electrostatic interaction calculations with a 12 Å cutoff distance. System charges were neutralized with NaCl, and the ion concentration was set to 0.15 M. The final protein structure was used for ligand docking.

The molecular docking site was selected using Site Finder, which is implemented in the MOE. The relaxed structure of the wild-type apo protein, obtained from the prior step, was used as the receptor for docking, subsequent simulations, and generation of the mutated proteins. Two hundred docking poses were generated using the triangle matcher method with a London Δ*G* scoring function.^108^ Twenty poses were then refined using the induced fit method and the GBVI/WSA Δ*G* scoring function. The protein–ligand pose with the lowest docking score was selected and minimized in MOE.^101,108^ Molecular dynamics simulations of the ligand-MmpL3 complexes were performed by using the same MD protocol previously described. The AM1-BCC method was used for the partial charge assignment for the ligands as implemented within the antechamber module in Amber 2022.^104,109^ The Ff14SB, GAFF2, and Tip4Pew force fields were used for the protein, ligands, and water, respectively.^105,110,111^ Simulations were performed in duplicate. Molecular Mechanics Poisson–Boltzmann Surface Area (MMPBSA) binding energies were calculated for the last 1 ns of the simulation.^104,112^ The average binding energy from the duplicate simulations was calculated.

### Drug Interaction Assay (DiaMOND)

Standard-of-care treatments of different classes (Table S5) were tested for their drug interactions with MmpL3 inhibitors by using the DiaMOND assay. The DiaMOND assay was conducted as described by Cokol et al., with minor modifications.^31,49^ Linear ranges of all the tested agents were established using the equation Δ*D* = (*M* – *m*)/(N – 1), where *M* and *m* are the lowest and highest concentrations, resulting in 100% and 0% growth inhibition, respectively, and *N* is the number of doses in the linear ranges. The linear ranges were constructed by starting with the dose *m* and adding Δ*D* to *N* – 1 times until *M* was reached. Following the null additivity model, Mab cultures, seeded at an OD_600_ of 0.1 in 96-well clear bottom plates, received either the null treatment (linear ranges of single agents [Χ_*n*_]) or the combination treatment ([1/2 Χ_*n*_] of two agents in combination). The plates were incubated for 3 days at 37 °C, and OD readings were taken to generate dose–response curves and interpolate the EC_50_ of the combinations. The drug interactions were evaluated by calculating the FIC_2_ (fraction of inhibitory concentration in a 2-drug combination) as described.^49^

### Transcriptional Profiling and Pathway Enrichment Analysis

In duplicates, 20 mL of an OD of 0.5 of *M. abscessus* culture was treated with 5× the MIC of the two MmpL3 inhibitors MSU-43085 and MSU-45683 (53 and 19 μM, respectively), or an equivalent volume of DMSO as the negative control, and incubated standing at 37 °C for 24 h. Total bacterial RNA was then extracted as described by Rohde *et al.*,^113^ and RNA-seq data was analyzed using the QIAGEN CLC genomics workbench software. The DMSO control was a shared control with another RNA-seq experiment performed in tandem and presented by Eke et al.^52^ and uploaded to the GEO Database (accession number GSE287881). Differentially expressed genes were defined as those with at least a 1.5-fold change from the control and an FDR *p*-value <0.05. This gene set (∼1260 gene (Figure S13)) was filtered based on the presence of annotations or confirmed orthologs (based on sequence identity) to 800 genes, which was used for pathway enrichment analysis using the Kyoto Encyclopedia of Genes and Genomes (KEGG)^62^ and by identifying and mapping orthologs of genes of *M. tuberculosis* H37Rv in the gene set and clustering them into pathways using orthovenn3.^61^ Data pooled from both methods were used to generate Figures S14–S16. RNA-seq data have been deposited in the GEO Database under the accession number (GSE296524).

### Quantification and Statistical Analysis

Data are presented as means ± the standard deviation from the mean. For all the experiments, “*n*” and “*m*” indicate the number of biological and technical replicates per treatment condition, respectively. Statistical analysis was performed using GraphPad Prism 10 and the MATLAB_R2024 b statistics package. The mutant fitness data were analyzed using one-way ANOVA, and a *p*-value of <0.05 was considered statistically significant. A *p* > 0.05 is indicated by ns, a *p* < 0.05 by *, a *p* < 0.01 by **, a *p* < 0.001 is by ***, and a *p* < 0.0001 by ****. All statistical details, including statistical tests and significance, are found in the figure legends or the method details section.

## Supplementary Material







## Notes

Supplemental figures and tables: Figures S1–S16 and Tables S1–S7, including processed and summarized data (e.g., dose–response and growth curves), characterization of mutants and clinical isolates, and breakdown of reagents and resources used in the study (PDF)
RNA-seq data set (XLSX)
Synthesis schemes and characterization by NMR (1H and 19F) and high-resolution mass spectrometry using electrospray ionization (HRMS-ESI) (PDF)
