# Supplementary material for: Defining the Mechanism of Action and Resistance of New Mycobacterium abscessus MmpL3 Inhibitors
Source: ACS Chem Biol. 2026 Jan 13;21(2):284–301. doi: 10.1021/acschembio.5c00709 (PMC12930390; doi:10.1021/acschembio.5c00709)
Supplement: Supplementary file 1 [file cb5c00709_si_001.pdf]

Supporting Information

Defining the mechanism of action and resistance of new *Mycobacterium abscessus* MmpL3 inhibitors

Bassel J. Abdalla[1,2], Matthew B. Giletto[2], Nazli Goksel Carpa[3], Angela K. Wilson[3], Edmund Ellsworth[2], & Robert B. Abramovitch[1,*]

[1]Department of Microbiology, Genetics & Immunology, Michigan State University, East Lansing, MI,48824

[2]Department of Pharmacology and Toxicology, Michigan State University, East Lansing, MI, 48824

[3]Department of Chemistry, Michigan State University, East Lansing, MI,48824

***Correspondence:** Robert B. Abramovitch

E-mail: abramov5@msu.edu

Phone: (517) 884-5416

Fax: (517) 353-8957

Supplemental Figure Legends

Figure S1: MmpL3 inhibitors exhibit a comparable activity to the most active of the standard-of-care treatments in *M. abscessus* ATCC 19977.

Figure S2: The dose-response curves of bone marrow-derived macrophages BMMΦ infected with *M. abscessus* ATCC 19977 expressing mEmerald GFP and treated with a panel of MmpL3 inhibitors. The MmpL3 inhibitors exhibit a strong intracellular bactericidal effect and are comparable in activity to rifabutin and clarithromycin, the gold standard of intracellular bactericidal efficacy for *M. abscessus.* Data are represented as the geometric mean of triplicates per condition, and error bars indicate the standard deviation.

Figure S3: Eukaryotic cytotoxicity assay of bone marrow-derived macrophages (BMMΦ) treated with a panel of 7 MmpL3 inhibitors, showing minimal to very low cytotoxicity of the inhibitors with reference to their therapeutic concentrations both *in vitro* and *ex vivo*. Data are represented as the geometric mean of triplicates for each condition, and error bars indicate the standard deviation. DMSO and 4% Triton X-100 were used as the negative and positive controls, respectively.

Figure S4: Thin-layer chromatography of total isolated lipids of *M. abscessus* treated with our panel of MmpL3 inhibitors and DMSO as a negative control, showing lower TDM presence in the treated groups relative to the negative control and demonstrating biochemical evidence for the proposed mechanism of action. Asterisks depict the expected location of TMM based on similar observations in *M. tuberculosis*.

Figure S5: The measured kinetics of fluorescence of $DioC_2$ (excitation at 485 nm and two emissions at 610 nm and 515 nm) in response to changes in the membrane potential of *M. abscessus* ATCC 19977, induced by the MmpL3 inhibitors, DMSO and CCCP (negative and positive controls). The figure shows the changes in fluorescence kinetics in terms of the ratio of the red/green emission fluorescence intensities, which correlates to the strength of changes in the membrane potential in response to different concentrations of the MmpL3 inhibitors **(160,40,10, 1 µM)**. HC-2091 and mixed series analogs (b, c, e, f, g) and (d), respectively, do not exhibit a membrane potential disruptive activity, while HC-2099 analogs (a) slightly dissipate membrane potential only at high concentrations (160 µM). A representative data set of a single experiment is represented as the geometric mean, and error bars indicate the standard deviation. n=2, m=3. µ

Figure S6: Non-replicative bactericidal assay of *M. abscessus* ATCC 19977 treated with different concentrations of 7 MmpL3 inhibitors (160,40,10, 1 µM), showing no significant difference from the negative control (DMSO). None of the MmpL3 inhibitors in this panel was shown to kill ***M. abscessus*** in a nutrient-starved, non-replicative condition, establishing cell replication as a condition for the mode of action of

this series of MmpL3 inhibitors. The dotted and solid lines represent the OD_{600} and CFU/mL counts, respectively. n=2, m=3. Data are represented as the geometric mean, and error bars indicate the standard deviation.

Figure S7: Biofilm disruption assay and cell viability assay in the biofilms showing the potential of MmpL3 inhibition in disrupting mature submerged *M. abscessus* ATCC 19977 biofilms and reducing the viability of cells inside the biofilm. MmpL3 inhibitors show considerable efficacy in disrupting the mature biofilm of *M. abscessus,* except MSU-45518 (e), and reduce the viability of cells in the biofilm. The difference between biofilm disruption and cell viability kinetics in terms of the EC_{50} for the analogs and standard-of-care treatments (SoCs) was observed and needs to be examined further. Standard-of-care treatments tested in the assays include Amikacin (h), Bedaquiline (i), Clarithromycin (j), and Tigecycline (k), all of which show similar trends, but overall enhanced activity compared to MmpL3 inhibitors. The inhibitors show similar activity to the SoCs in the viability assay, and less comparable activity in terms of the biomass of the biofilm. Data are represented as the geometric mean, and error bars indicate the standard deviation, n=3, m=3.

Figure S8: MmpL3 inhibitors have a very narrow spectrum of activity and are exclusive to Mycobacteria (i.e., *M. tuberculosis* (a)Erdman, b) CDC-1551, and c) H37Rv*)* and d) *M. smegmatis (mc^{2}155*). e) The EC50 values of the different analogs against each strain in μM*.* n=2, m=3*.* Data are represented as the geometric mean, and error bars indicate the standard deviation.

Figure S9: Dose-response curves of the additional HC-2099 analogs and Mixed-series analogs used in the mutant cross-resistance assay against WT *M. abscessus* ATCC 19977. a) EC_{50} values of the different analogs in **μ**M. b) Dose-response curves of the analogs, spanning different ranges of activity, which might be attributed to how the parent scaffold is substituted**.** c) structures of the additional analogs categorized by parent scaffold. n=2 and m=3. Data are represented as the geometric mean, and error bars indicate the standard deviation.

Figure S10: The relative fitness of MmpL3 mutants and WT. a) Growth curves of the panel of mutants and the WT *M. abscessus* ATCC 19977. b) The AUC values calculated using the growth curves.

Figure S11: Fitness profiling of the 16 *mmpL3* mutant strains depicting mutants exhibiting a putative fitness defect phenotype. Fitness assessment was conducted using the areas under the growth curves of the mutants normalized to that of the wild type (See Fig. S10). A ratio of less than 1 indicates a lower growth rate than the WT, which hints at a putative fitness defect. One-way ANOVA was performed to analyze the fitness data, **** $p<0.0001$. n= 3, m= 12.

Figure S12: Drug sensitivity assessment of a subset of the 16 *mmpL3* mutants exhibiting major fitness defects, displaying correlation between fitness and drug

sensitivity. Mutants displaying major defects in growth were selectively tested for sensitivity against a) Clarithromycin, and b) Amikacin, two commonly prescribed standard-of-care treatments, and c) Meropenem, a drug targeting the cell envelope, showing more sensitivity and hinting at the impact of fitness-altering mutations on drug sensitization. d) EC50s of the drugs against the mutants, presented in nM. Mutants labeled with * are in an isogenic background to the wild type. e) Normalized EC_{50} of the three treatments in the Mutants against that of the wildtype. SoCs: Standard-of-care treatments.

Figure S13: A summary of differentially expressed genes in response to MmpL3 inhibition, showing alterations in one-fifth of the Mab genome. Treatment with MSU-43085 and MSU-45683 (5x the MIC) causes alterations in 1809 and 2033 genes with at least a 1.5-fold change. The expression of 1258 genes are commonly altered by both treatments, representing genes differentially associated with MmpL3 inhibition.

Figure S14. Selected heat maps of the RNA-seq transcriptome analysis in *Mab* under MmpL3 inhibition showing differential expression in relevant pathways. The panel summarizes the response of *Mab* under MmpL3 inhibition, showing a strong upregulation of several pathways involved in stress mitigation (i.e., osmotic stress, envelope stress, and oxidative stress) and perturbations in several membrane lipid synthesis pathways (i.e., Mycolic acid and various glycolipids), potentially impacting the general cell envelope integrity not only that of the mycomembrane. While all MCE loci are either downregulated or normally expressed, a single locus, downstream of the *mmpL3* gene (MAB_4508), is the only upregulated MCE locus, suggesting a potential role for mitigating MmpL3-induced envelope stress. CA: Carbonic anhydrase.

Figure S15: Heat maps of the RNA-seq transcriptome analysis in *M. abscessus* ATCC 19977 treated with MSU-43085 and MSU-45683, focusing on signaling, nutrient transport, and stress response. The Panel depicts the response to MmpL3 inhibition in Mab on genetic, signaling, and homeostatic levels, FC: Fold change from the negative control (DMSO), ESX: ESAT6 secretion (aka type VII secretion), MCE: Mammalian cell entry, ABC: ATP-binding cassette. n=1, m=2.

Figure S16: Heat maps of the RNA-seq transcriptome analysis in *M. abscessus* ATCC 19977 treated with MSU-43085 and MSU-45683, focusing on metabolic and biochemical responses. The panel focuses on changes in central metabolism, energetics, and the biosynthetic capacity of the bacteria, with emphasis on membrane lipid synthesis (Glycolipids and Mycolic acid). * Locus 2 includes genes that are not within the locus (MAB_4098c -MAB_4117c) but have a predicted functional association based on their orthologs in *m. tuberculosis*.

Figure S17: The efficacy of the MmpL3 inhibitor panel in *M. abscessus*, used alone or in combination with verapamil (VER), an inhibitor of drug efflux, showing no significant difference.

Figure S18: Genome alignment for a subset of clinical isolates (25 isolates) with the reference genome of *M. abscessus* ATCC 19977, where bands in violet are conserved stretches of open reading frames (ORFs), while those in white are ORFs with multiple genomic variations (SNPs and Indels). Alignments show a high level of variation between several clinical isolates and do not reveal any correlations between the phylogeny of the isolates and the resistance/sensitivity profiles against MmpL3 inhibitors (Figure 5). However, it sets the stage for a genome-wide association study to examine them.

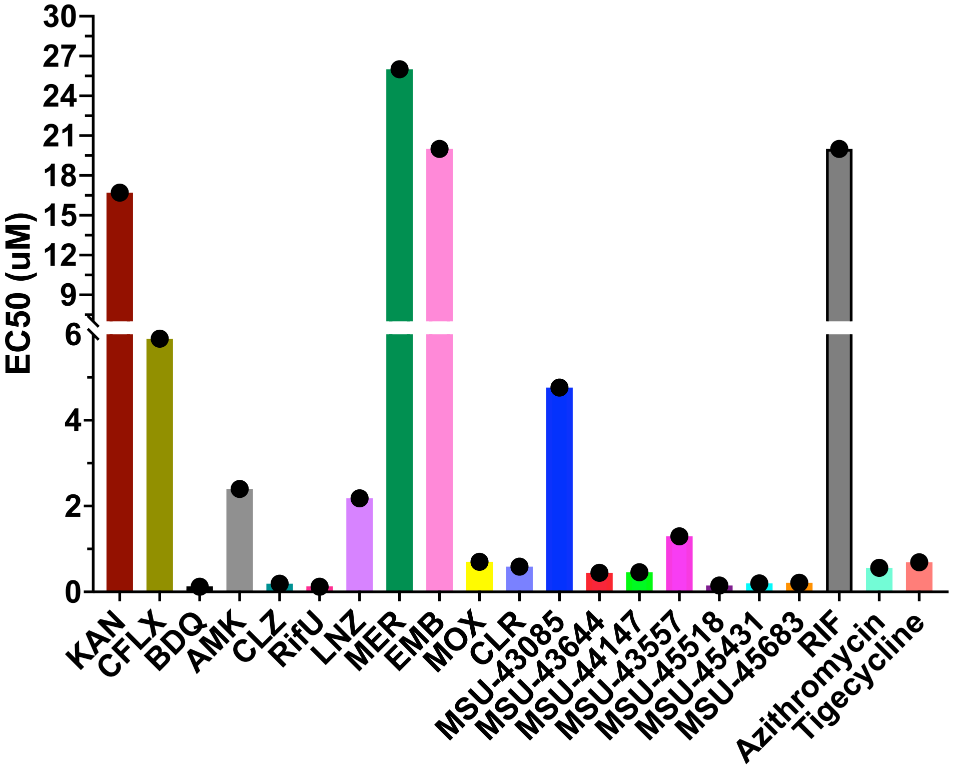

Supplemental Figure 1

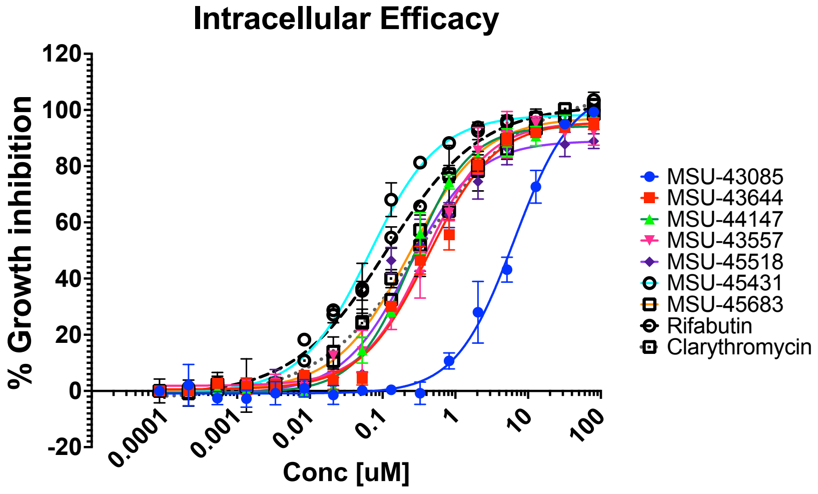

	MSU-43085	MSU-43644	MSU-44147	MSU-43557	MSU-45518	MSU-45431	MSU-45683	Rifabutin	Clarythromycin
HillSlope	1.078	0.9414	1.119	1.011	0.9425	0.9147	0.8591	0.6732	0.6067
IC50	6.426	0.4214	0.2557	0.3889	0.2309	0.06568	0.2327	0.1146	0.3411

Supplemental Figure 2

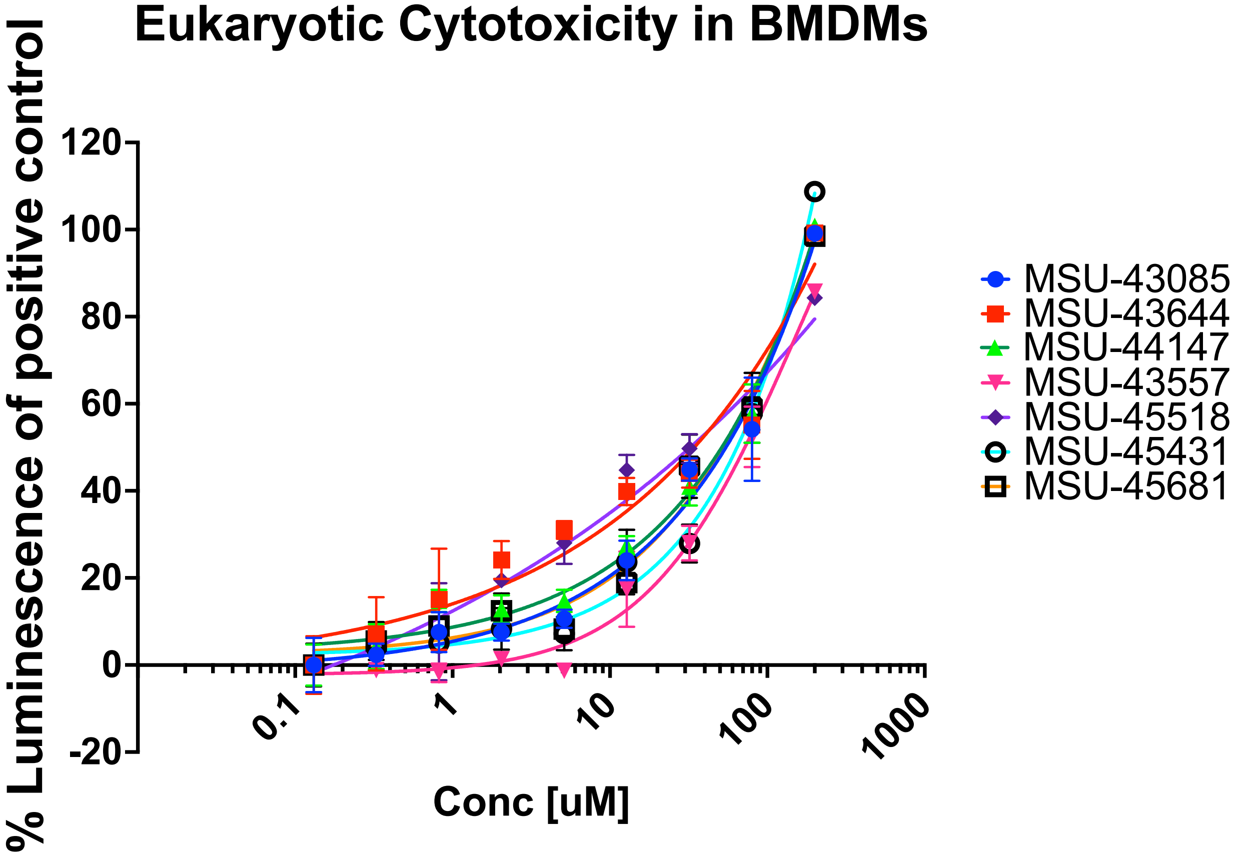

Supplemental Figure 3

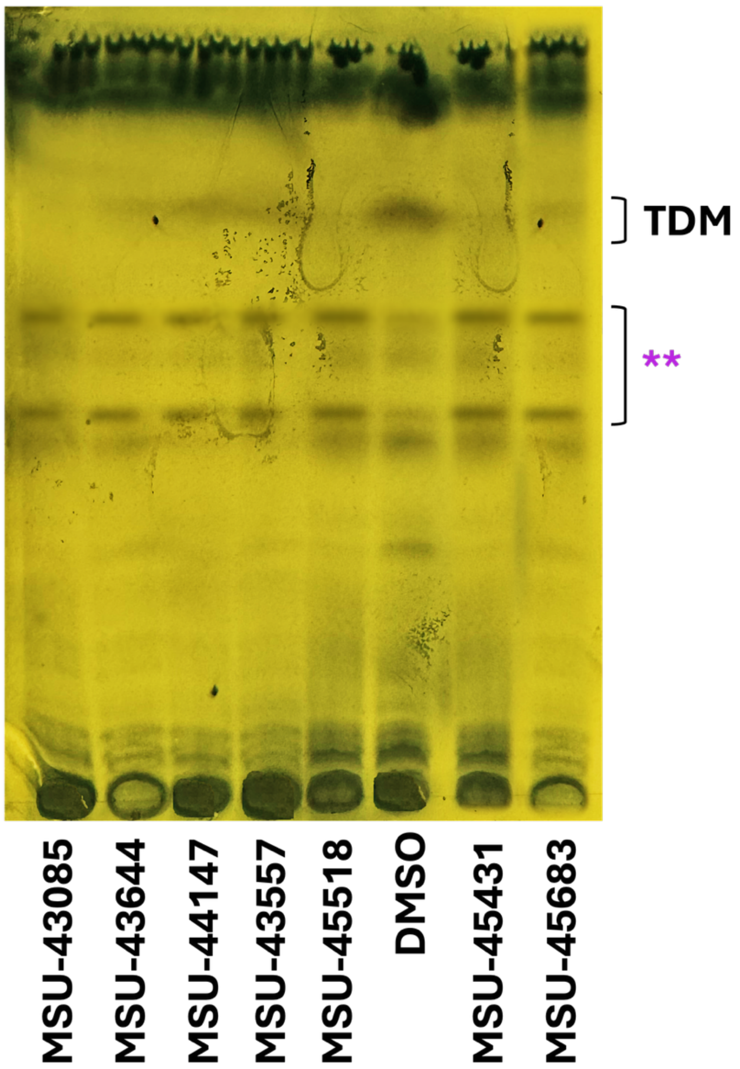

Supplemental Figure 4

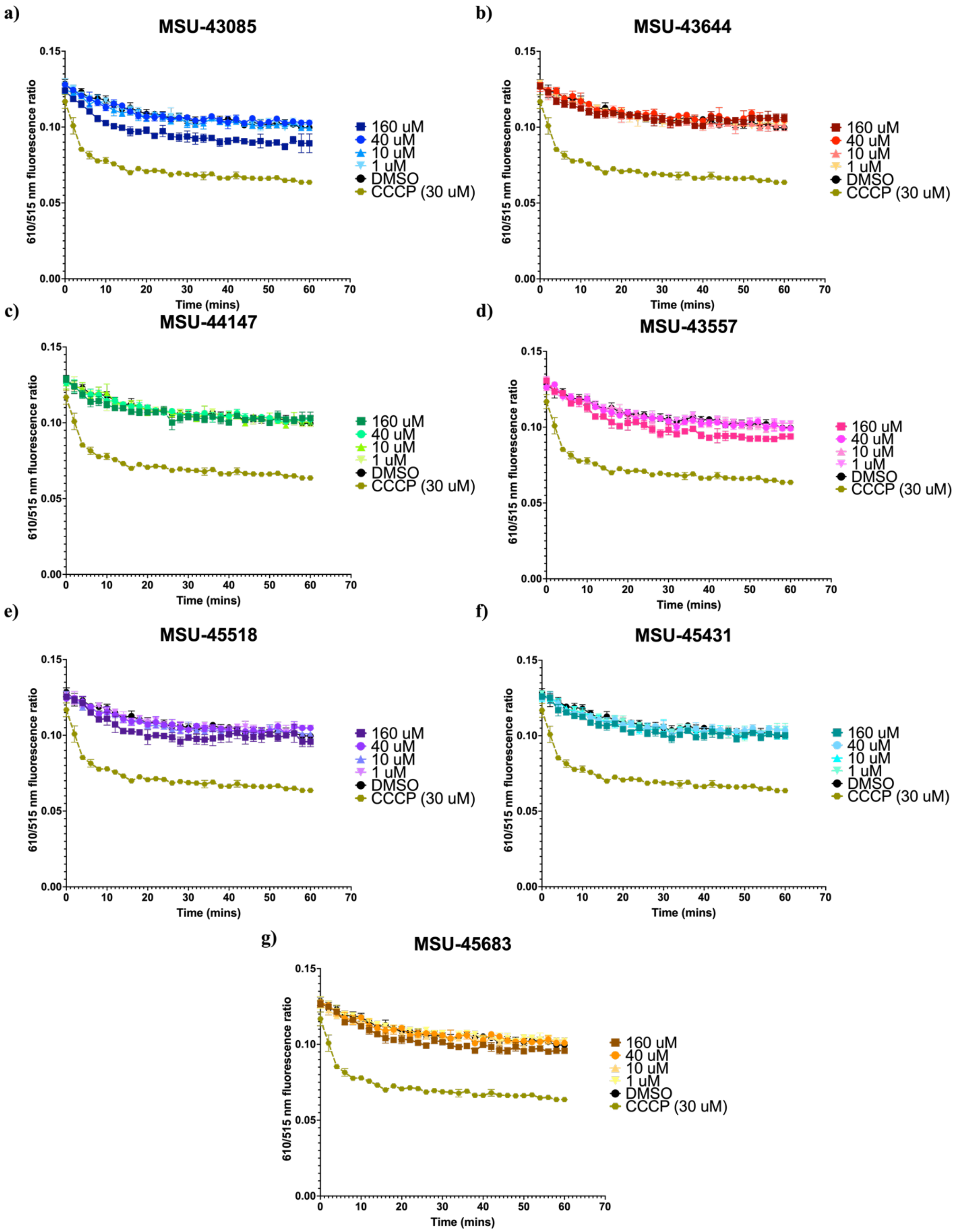

Supplemental Figure 5

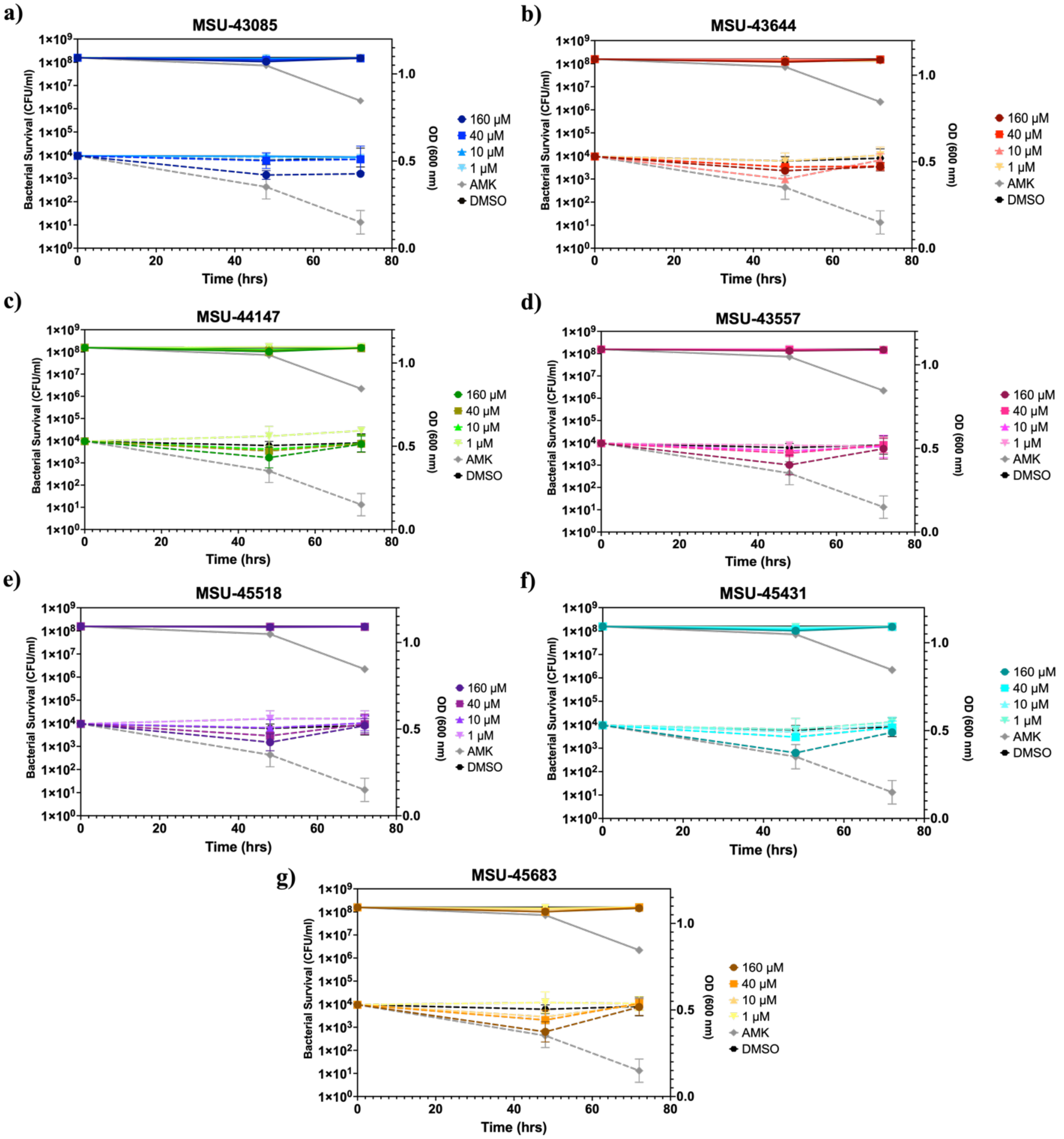

Supplemental Figure 6

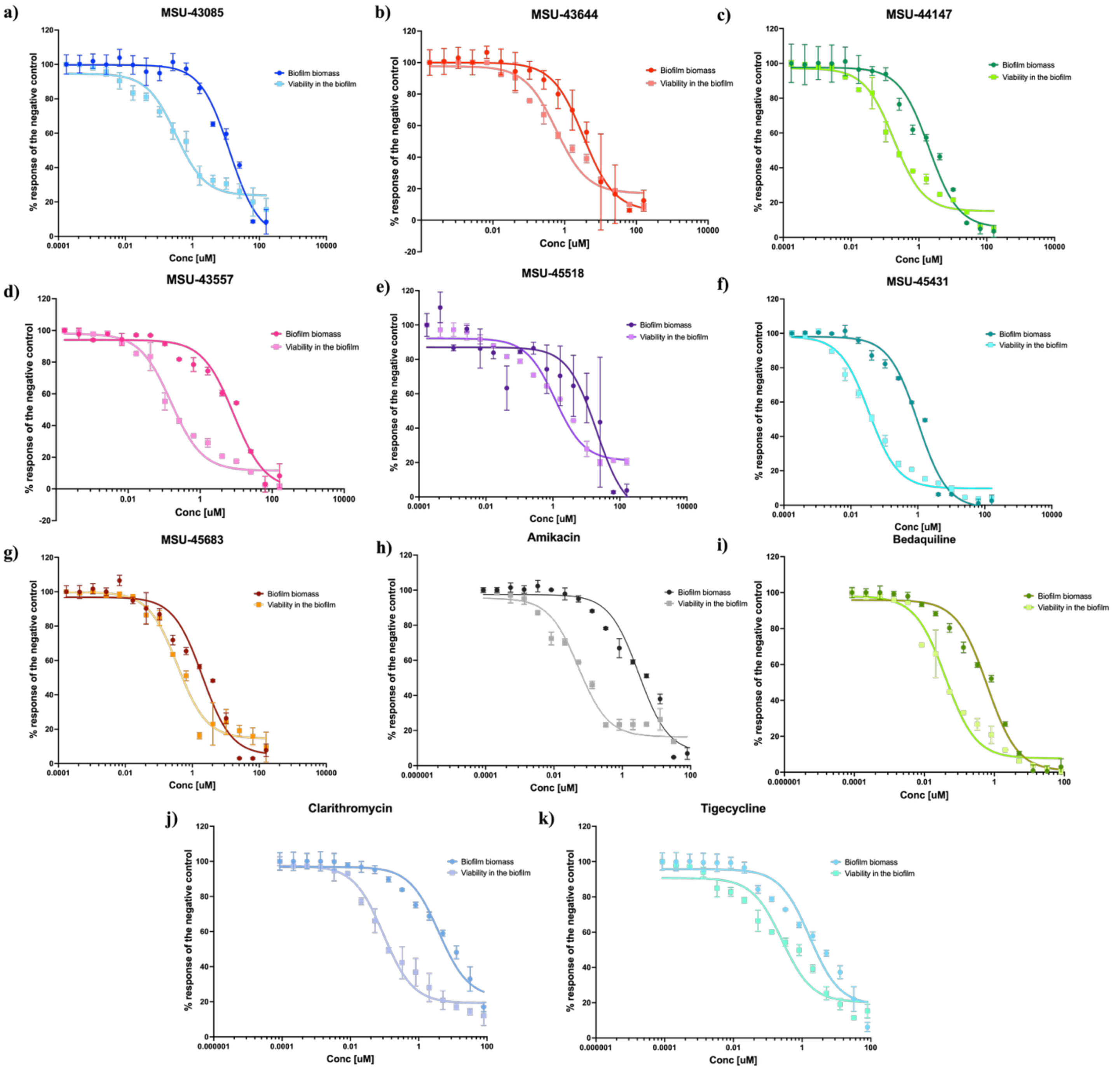

Supplemental Figure 7

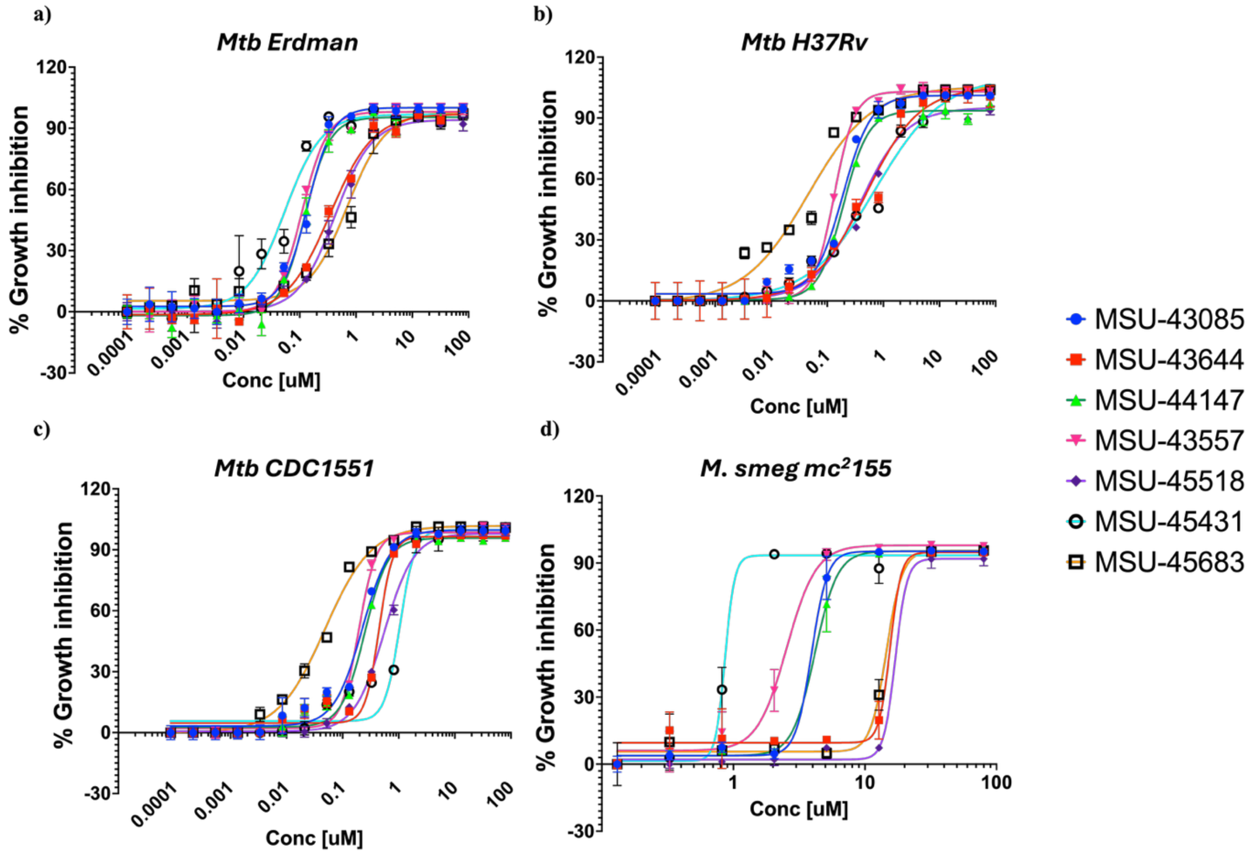

Supplemental Figure 8

a)

	MSU-45655	MSU-43186	MSU-45540	MSU-45516	MSU-45538	MSU-45606	MSU-45819	MSU-45350
**EC_{50} [uM]**	0.189	1.202	0.680	1.758	0.217	0.321	0.656	1.087

b)

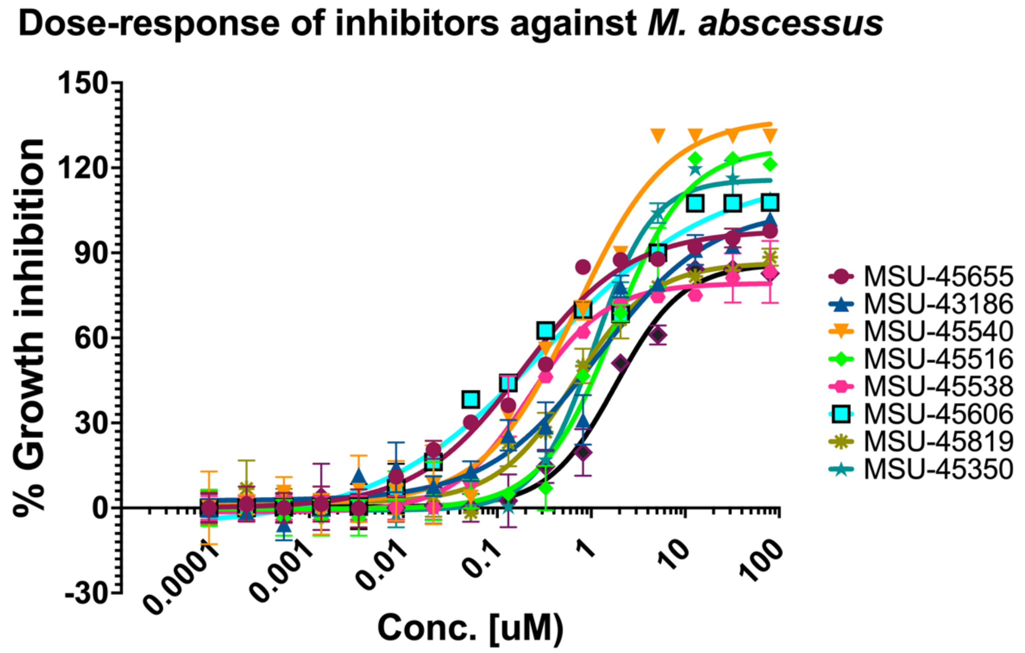

c)

HC2099 Analogs

MSU-45655

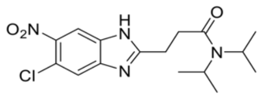

MSU-43186

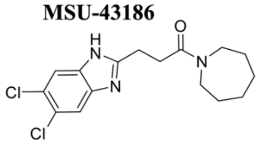

MSU-45540

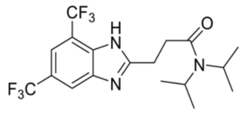

MSU-45516

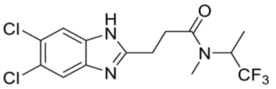

MSU-45538

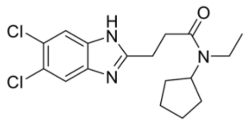

Mixed Analogs

MSU-45350

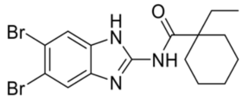

MSU-45606

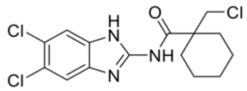

MSU-45819

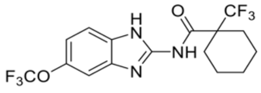

Supplemental Figure 9

a)

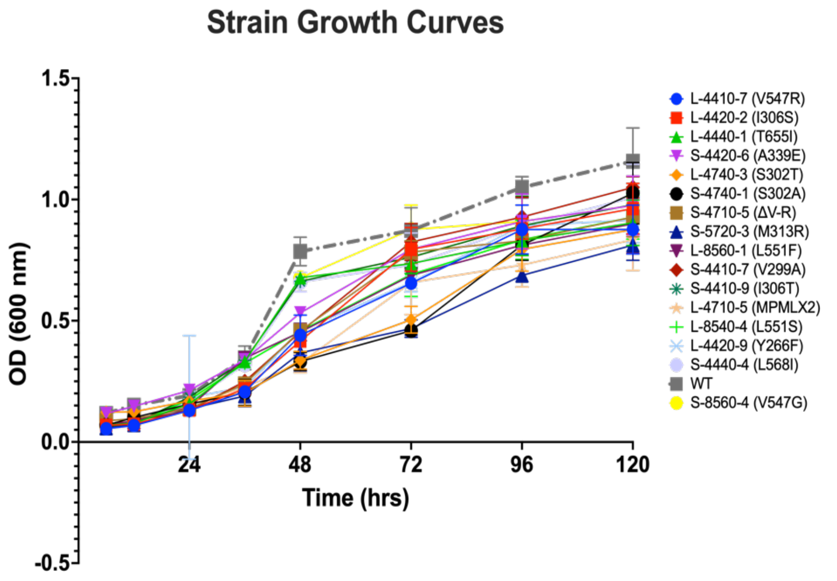

b)

	Strain	AUC
1	L-4410-7 (V547R)	59.98
2	L-4420-2 (I306S)	64.29
3	L-4440-1 (T655I)	67.39
4	S-4420-6 (A339E)	70.48
5	L-4740-3 (S302T)	53.69
6	s-4740-1 (S302A)	54.21
7	S-5720-3 (M313R)	48.79
8	L-8560-1 (L551F)	61.79
9	S-4410-7 (V299A)	76.5
10	S-4410-9 (I306T)	68.37
11	L-4710-5 (MPMLX2)	54.55
12	L-8540-4 (L551S)	62.81
13	S-4710-5 (V-R)	74.98
14	L-4420-9 (Y266F)	62.13
15	S-4440-4 (L568I)	69.62
16	S-8560-4 (V547G)	73.55
17	WT	82.35

Supplemental Figure 10

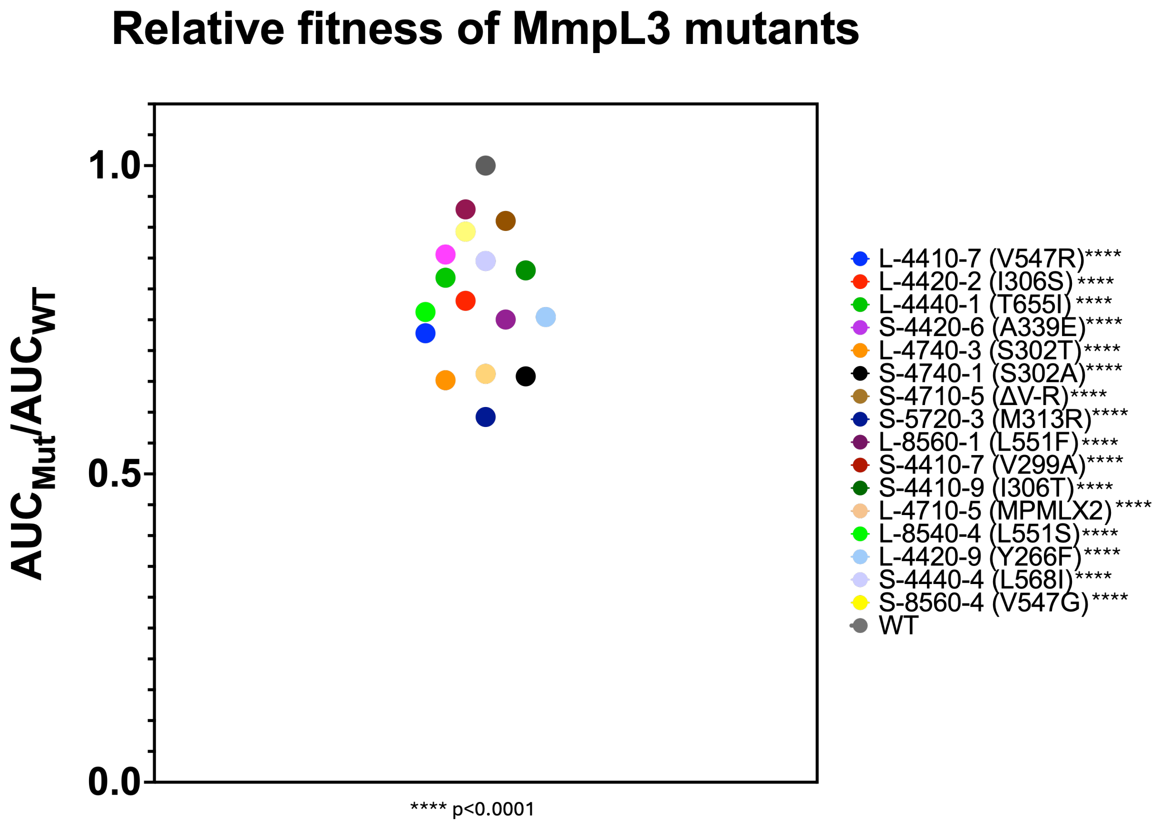

Supplemental Figure 11

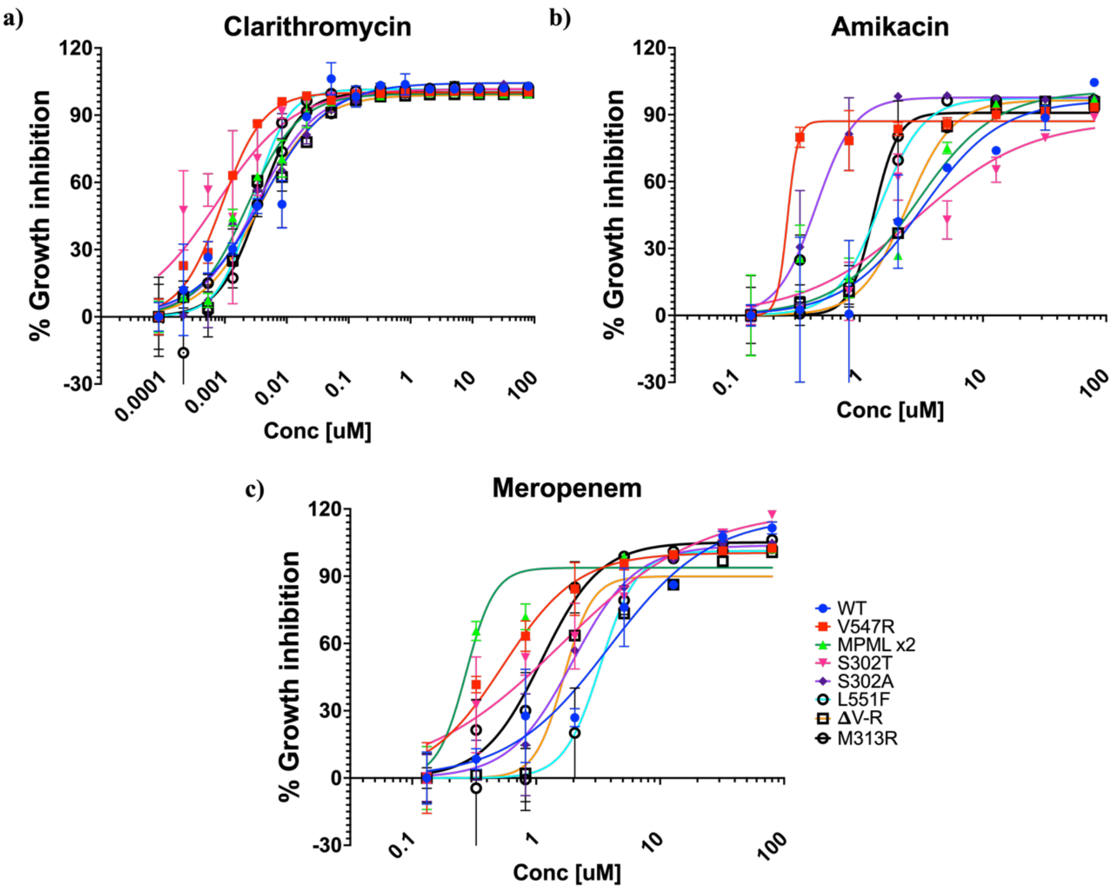

d)

	Clarithromycin	Amikacin	Meropenem
WT	3.9	3,233	3,886
V547R*	0.9	260.0	541.0
MPML x2	2.3	2,866	272.0
S302T	0.7	2,890	1,621
S302A*	3.3	444.0	1,966
L551F*	2.8	1,451	3,316
ΔV-R	3.7	2,408	1,668
M313R*	3.6	1,303	1,107

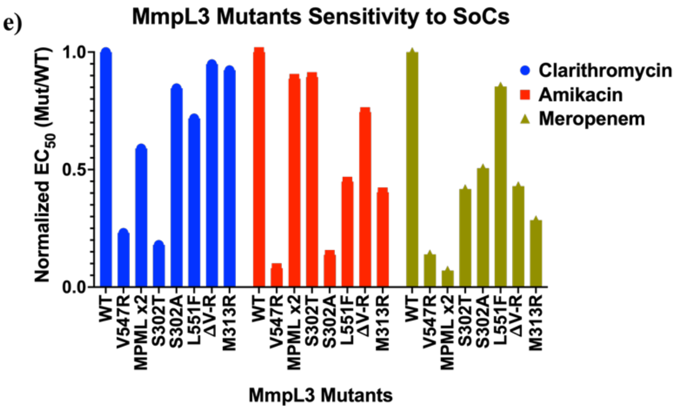

Supplemental Figure 12

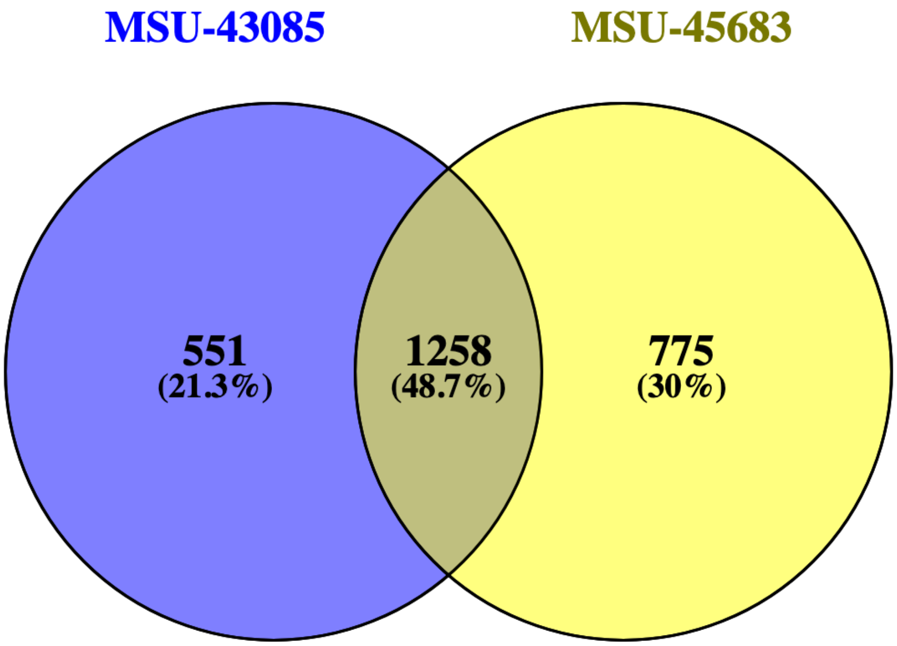

Supplemental Figure 13

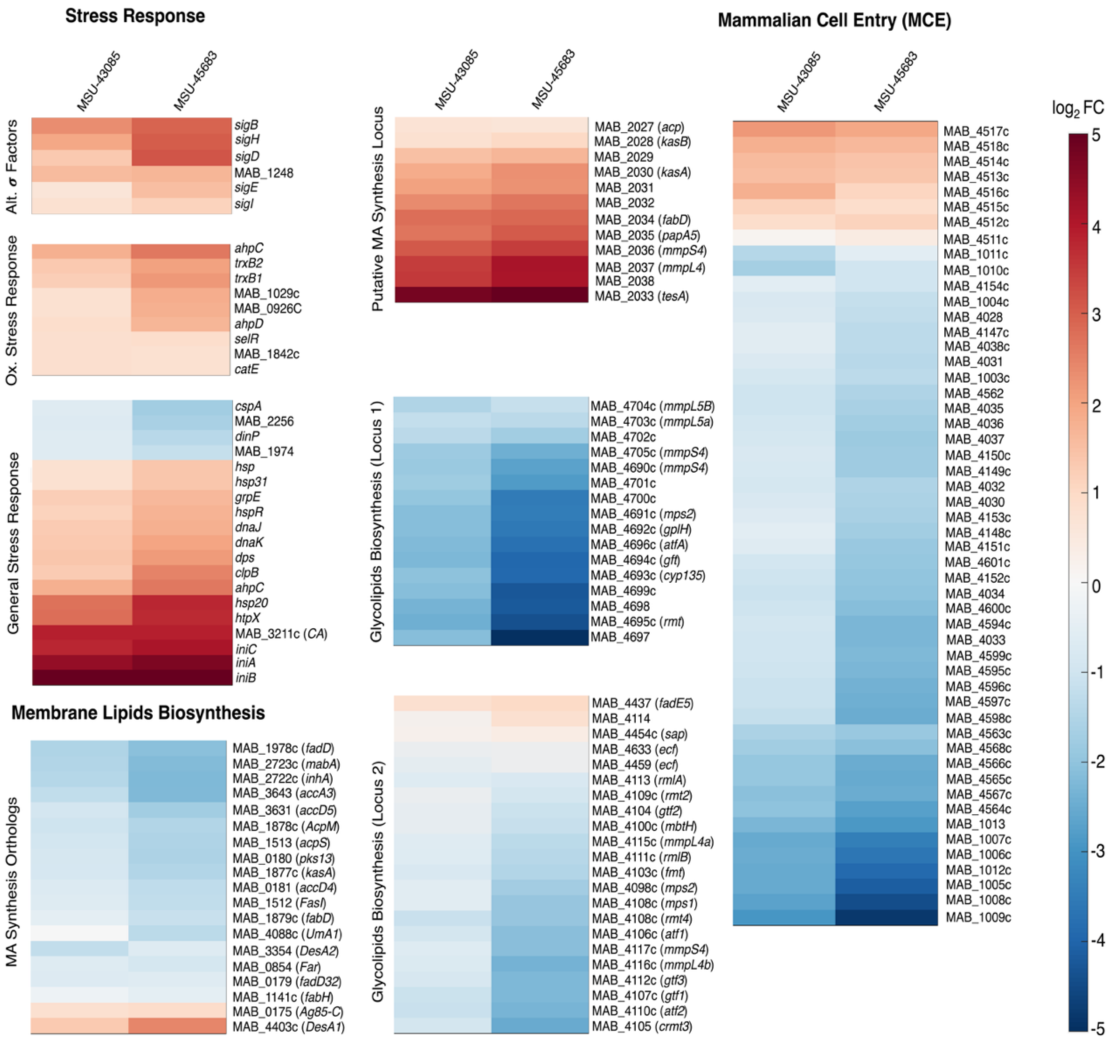

Supplemental Figure 14

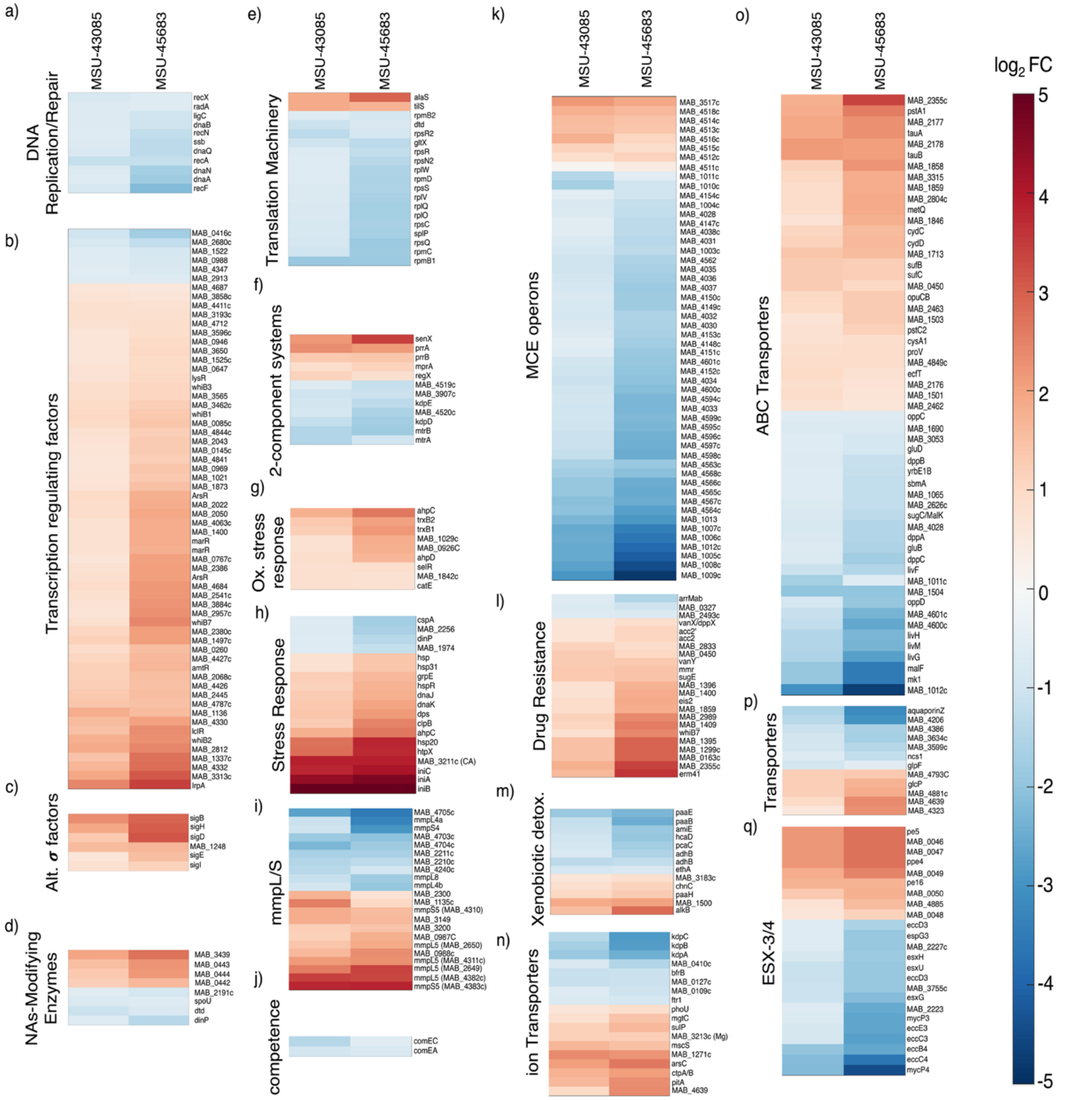

Supplemental Figure 15

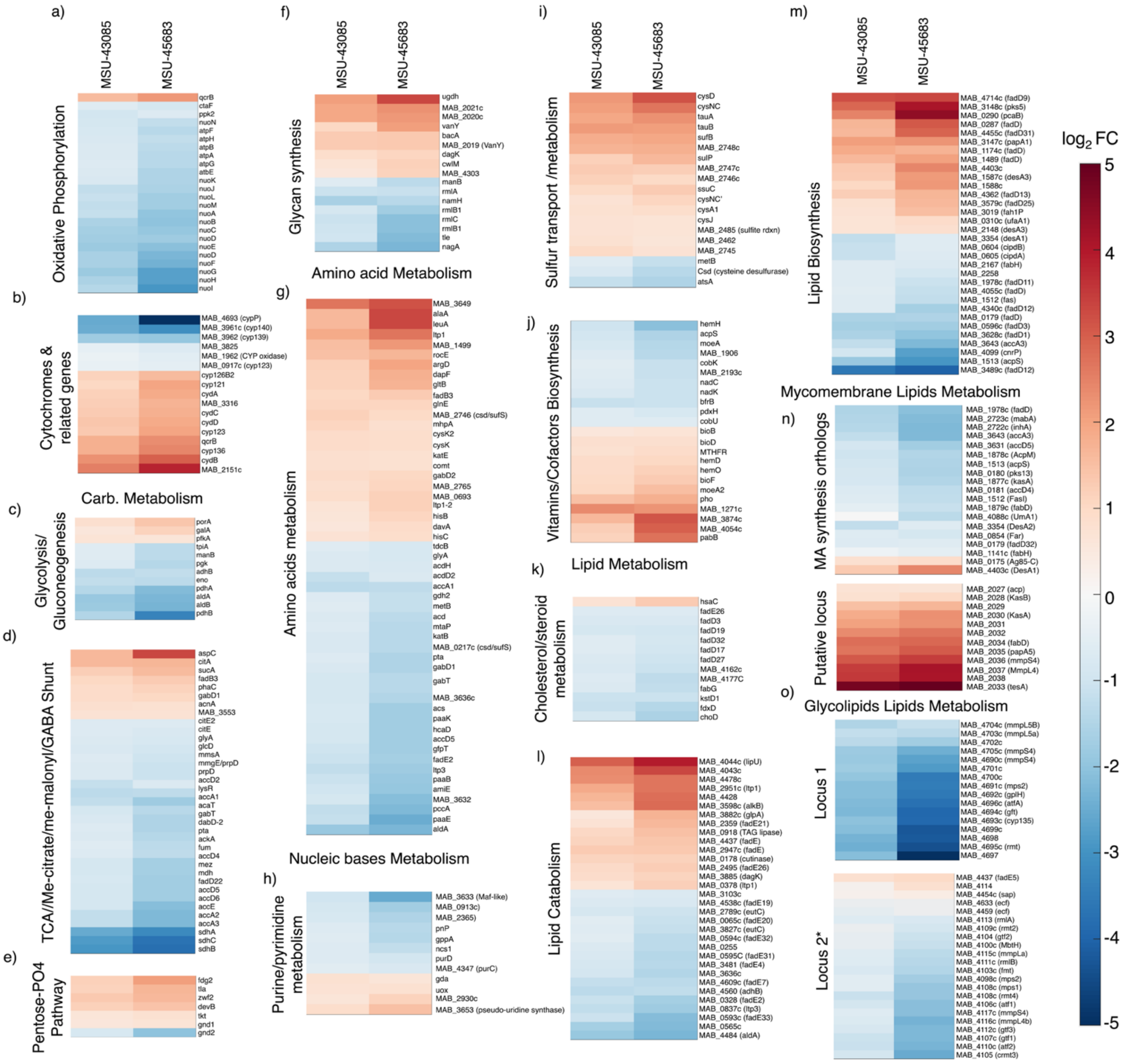

Supplemental Figure 16

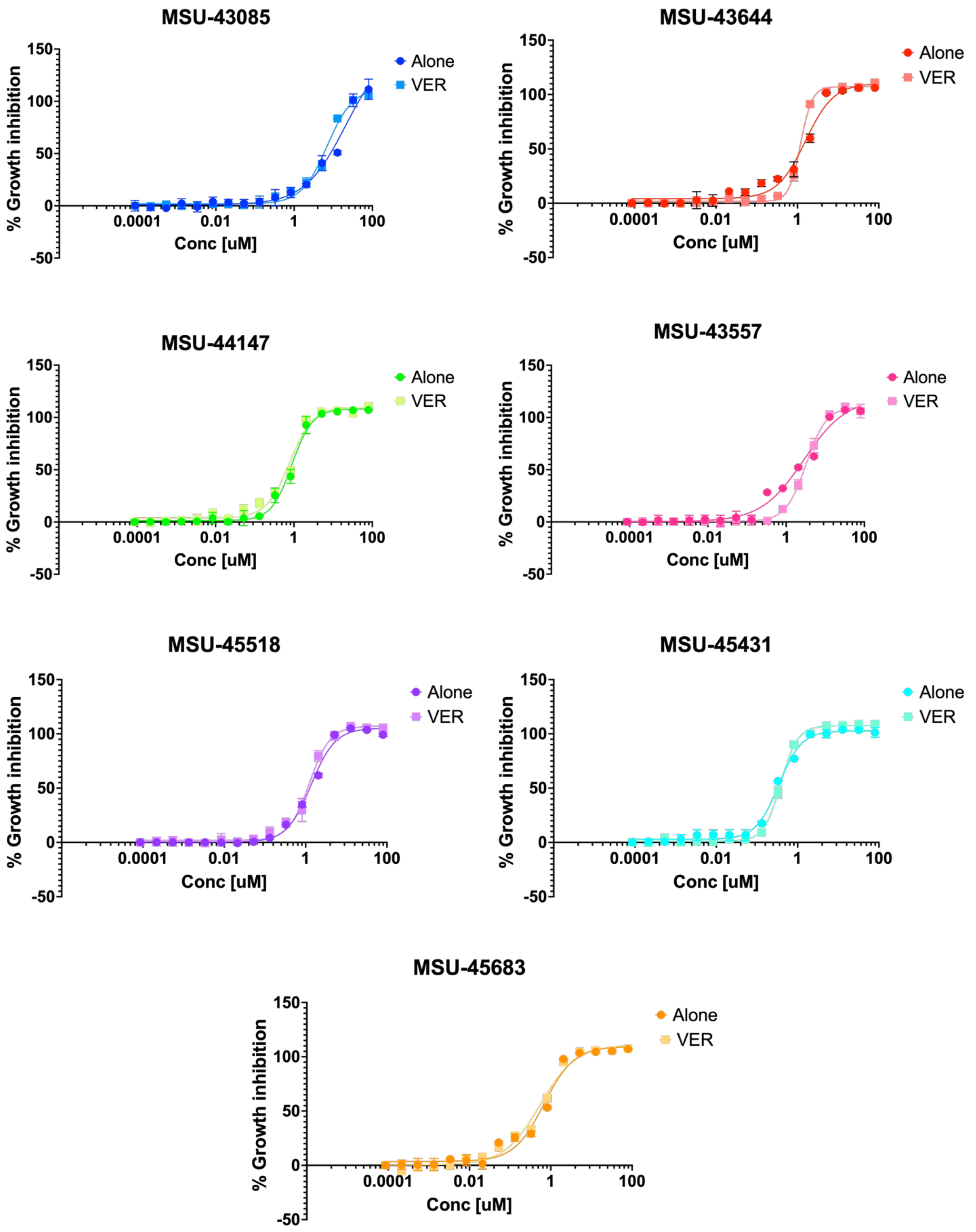

Supplemental Figure 17

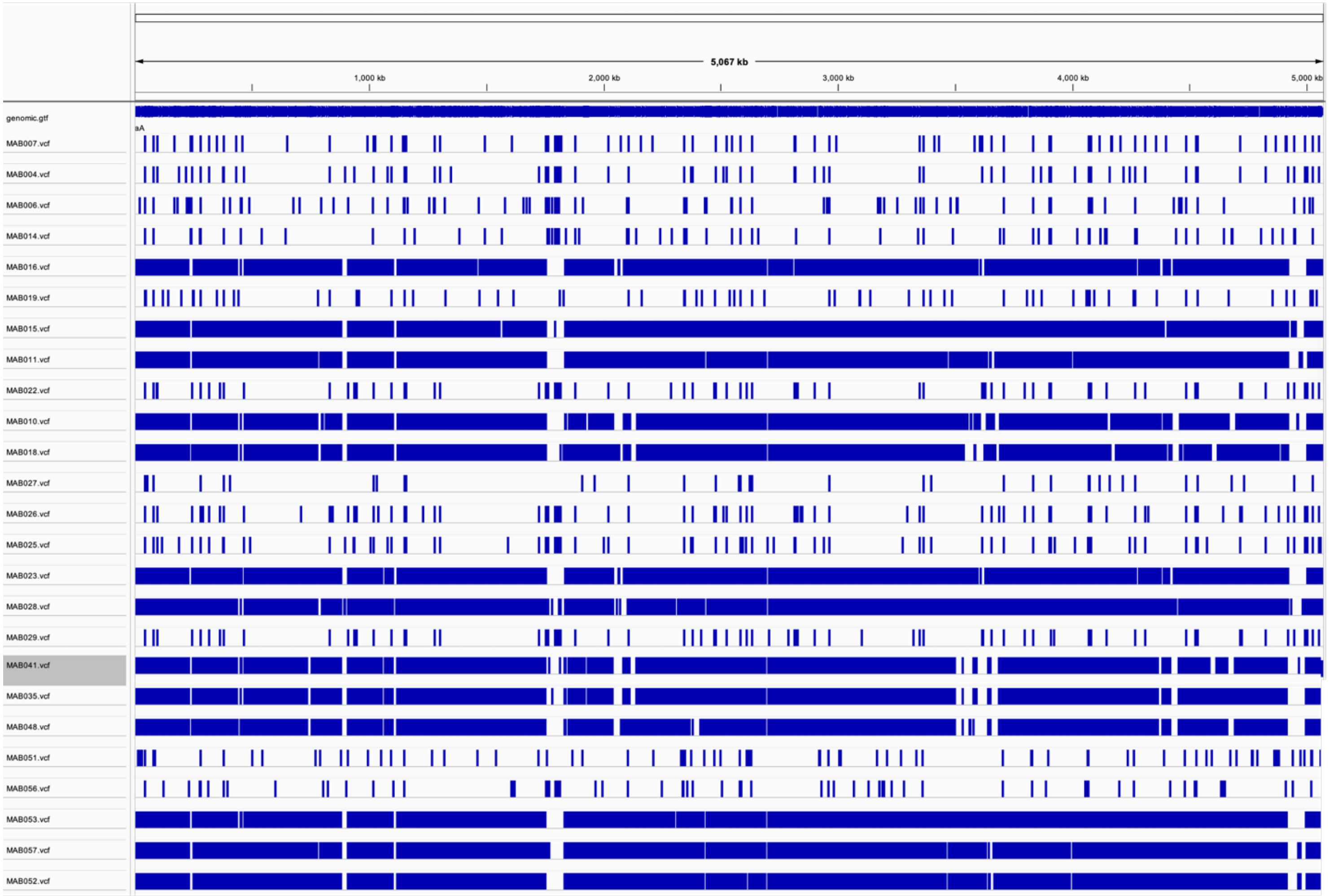

Supplemental Figure 18

Supplementary Tables

Table S1: Characterization of the mutants isolated against a subset of the MmpL3 inhibitors. Mutants are annotated based on several factors: 1- the size of the isolated colony, Small or Large (S/L), the last two digits of the inhibitor they were isolated against (85,57,47 and 44), the concentration of the analog (10, 20, 40 and 60 µM) and an index to indicate the different mutants. Mutants labeled with * have a single mutation in the MmpL3 gene in an isogenic environment to the WT. Sequencing Data are summarized in terms of SNP location, the gene, the nucleotide, and the associated amino acid change. Mutants labeled with † are, to the best of our knowledge, novel to this study and were not reported in the literature.

#	Compound	Isolated Strain	SNP location	Gene	Mutation	AA change
1	**MSU-43085**	**L-8560-1***	1,722,967	MAB_4508 (*mmpL3*)	**TTG→TTT**	L551F
2		**L-8540-4**	1,722,966		**TTG→TCG**	L551S
3		**S-8560-4**	1,722,954		**GTG→GGG**	V547G
4	**MSU-43557**	**S-5720-3*†**	1,722,252		**ATG→AGG**	M313R
5	**MSU-44147**	**L-4710-5†**	1,722,258		**(GCCGATGCTGAT)$_{1\to2}$**	M311-L314 X2
6		**S-4710-5†**	1,722,358		**Δ12 bp**	Δ V349-R352
7		**L-4740-3†**	1,722,218		**TCC→ACC**	S302T
8		**S-4740-1***	1,722,218		**TCC→GCC**	S302A
9	**MSU-43644**	**S-4440-4†**	1,723,016		**CTA→ATA**	L568I
10		**S-4410-9†**	1,722,231		**ATC→ACC**	I306T
11		**S-4410-7**	1,722,210		**GTC→GCC**	V299A
12		**S-4420-6*†**	1,722,330		**GCG→GAG**	A339E
13		**L-4440-1†**	1,723,278		**ACC→ATC**	T655I
14		**L-4420-2***	1,722,231		**ATC→AGC)**	I306S

15		L-4410-7*†	1,722,953		GTG→AGG	V547R
16		L-4420-9†	1,722,111		TAT→TTT	Y266F

Table S2: AUC values of dose-response curves from the cross-resistance study.

	MSU-43085	MSU-43644	MSU-44147	MSU-43557	MSU-45518	MSU-45431	MSU-45683	MSU-45655	MSU-43186	MSU-45540	MSU-45516	MSU-45538	MSU-45606	MSU-45819	MSU-45350	MSU-45821
**V547R**	73.29	23.12	26.41	116.5	13.21	205.1	94.5	129.36	101.371	135.48	95.959	97.347	156.47	94.77	130.47	109.4
**I306S**	104	60.2	135	149.5	245.2	185.4	180.8	175.43	119.28	194.28	146.31	129.38	187.37	134.237	161.2	80.347
**T655I**	126.1	189.23	231.21	173.27	293.21	243.31	245.35	232.21	183.48	283.1	187.31	154.32	232.1	167.21	209.21	143.1
**A339E**	147.5	74	139.2	110.6	53	62	66	221.45	187.4	278.37	230.4	165.46	113.2	112.39	131	73.58
**S302T**	124.37	214.37	197.37	143.21	23	12	23	174.37	114.38	212.31	156.27	132.19	184.31	143.2	178.39	123.31
**S302A**	136.9	56.88	142.7	135.2	25.23	129.1	85.15	250.3	197.47	276.437	195.46	193.9	174.47	132.39	143.38	95.38
**Δ V349-R352**	11.95	49.69	52.53	67.96	2.575	153.9	106.9	18.347	28.31	33.45	20.43	17.56	70.25	60.27	101.5	52.77
**M313R**	42.9	72.05	69.63	72.74	301	166.3	163.7	78.38	49.458	73.47	83.47	49.638	115.5	82	59.5	63.27
**L551F**	55.97	88.99	95.86	110.6	46.12	186.4	231.21	102.68	85.63	120.36	89.237	82.17	132.41	93.28	106.36	72.17
**V299A**	30.63	53.17	174.95	70.13	8.19	206.8	143.3	64.47	44.59	52.37	63.47	45.517	89.73	73.21	36.58	61
**I306T**	118.9	75.93	104.1	185.9	133.9	191.2	155.7	231.2	163.48	254.38	163.47	182.38	209.12	163.21	195.36	131.19
**M311-L314 x2**	13.49	66.18	107	102.4	25.37	123.4	88.5	27	26.39	23.45	21	23.45	45.31	32.43	33.21	23.42
**L551S**	43	80.44	130.4	126.9	24.36	169.3	231.12	43.43	34.38	73.47	23.46	25.36	165.437	138	158.48	75.58
**Y266F**	89.23	80.44	130.4	126.9	77.37	169.3	104.3	166.321	153.487	189.253	142.948	121.39	142.37	100.4	152.3	72.38
**L568I**	125.1	76.03	88.77	99.1	106.8	174.6	87.32	193.4	189.41	278.34	189.27	152.36	127.36	99.27	101.21	68.37
**V547G**	82.12	54.31	85.38	143.1	23.21	232.12	125.21	154.12	143.12	167.98	167.38	153.21	167.21	132.12	178.31	129.12
**WT**	141	229.6	260.1	191.4	307	282.7	267.9	256.7	211.6	293.1	219.6	197.9	265.1	188.2	215.7	144.9

Table S3: Clinical isolates characterization summary, including site of isolation, morphology, MmpL3 mutations, drug resistance, and sensitivity profiles based on CLSI guidelines and cutoffs [(1), (2)]. Multi-drug resistance (MDR) is designated for strains exhibiting Intermediate or total resistance to two or more different drug classes. SXT: Sulfamethoxazole and Trimethoprim.

Isolate code	Morphology	Isolation site	MmpL3 Mutations?	Resistance Status	Resistance profile	Sensitivity Profile
**MAB002**	Smooth	Arm	N/A	MDR	Cefoxitin, Ciprofloxacin, Doxycycline, Imipenem, Minocycline, Moxifloxacin, Tobramycin, SXT	Amikacin, Clarithromycin, Linezolid, Tigecycline
**MAB006**	Smooth	Sputum	N/A	MDR	Amikacin, Cefoxitin, Ciprofloxacin, Clarithromycin, Doxycycline, Imipenem,	Linezolid, Tigecycline

					Minocycline, Moxifloxacin, Tobramycin, SXT	
**MAB007**	Smooth	Spine	N/A	MDR	Cefoxitin, Ciprofloxacin, Clarithromycin, Doxycycline, Imipenem, Linezolid, Minocycline, Moxifloxacin, Tobramycin, SXT	Amikacin, Tigecycline
**MAB004**	Rough	Sputum	N/A	MDR	Amikacin, Cefoxitin, Ciprofloxacin, Clarithromycin, Doxycycline, Imipenem, Minocycline, Moxifloxacin, Tobramycin, SXT	Linezolid, Tigecycline
**MAB010**	Smooth	Lung	N/A	MDR	Amikacin, Cefoxitin, Ciprofloxacin, Doxycycline, Imipenem, Linezolid, Minocycline, Moxifloxacin, Tobramycin, SXT	Clarithromycin, Tigecycline
**MAB011**	Smooth	Sputum	M300I	MDR	Amikacin, Cefoxitin, Ciprofloxacin, Doxycycline, Imipenem,	Tigecycline

					Linezolid, Moxifloxacin, Tobramycin, SXT	
**MAB014**	Rough	Sputum	N/A	MDR	Cefoxitin, Ciprofloxacin, Clarithromycin, Doxycycline, Imipenem, Minocycline, Moxifloxacin, Tobramycin, SXT	Amikacin, Linezolid, Tigecycline
**MAB015**	Rough	Sputum	N/A	MDR	Cefoxitin, Ciprofloxacin, Clarithromycin, Doxycycline, Imipenem, Linezolid, Minocycline, Moxifloxacin, Tobramycin, SXT	Amikacin, Tigecycline
**MAB016**	Smooth	Breast	N/A	MDR	Cefoxitin, Ciprofloxacin, Clarithromycin, Doxycycline, Imipenem, Linezolid, Minocycline, Moxifloxacin, Tobramycin, SXT	Amikacin, Tigecycline
**MAB018**	Smooth	Sputum	Q973*	MDR	Cefoxitin, Ciprofloxacin, Clarithromycin, Doxycycline, Imipenem, Minocycline,	Amikacin, Linezolid, Tigecycline

					Moxifloxacin, Tobramycin, SXT	
**MAB019**	Rough	Bronchial Wash	N/A	N/A	N/A	N/A
**MAB022**	Smooth	Sputum	N/A	MDR	Cefoxitin, Ciprofloxacin, Clarithromycin, Doxycycline, Imipenem, Linezolid, Moxifloxacin, Tobramycin, SXT	Amikacin, Tigecycline
**MAB023**	Smooth	Sputum	N/A	MDR	Cefoxitin, Ciprofloxacin, Clarithromycin, Doxycycline, Imipenem, Linezolid, Moxifloxacin, Tobramycin, SXT	Amikacin, Tigecycline
**MAB025**	Rough	Sputum	N/A	MDR	Amikacin, Cefoxitin, Ciprofloxacin, Clarithromycin, Doxycycline, Imipenem, Linezolid, Moxifloxacin, Tobramycin, SXT	Tigecycline
**MAB026**	Smooth	Abscess	N/A	MDR	Amikacin, Cefoxitin, Ciprofloxacin, Clarithromycin, Doxycycline, Imipenem,	Tigecycline

					Linezolid, Moxifloxacin, Tobramycin, SXT	
**MAB028**	Rough	Sputum	P163R	MDR	Amikacin, Cefoxitin, Ciprofloxacin, Clarithromycin, Doxycycline, Imipenem, Minocycline, Moxifloxacin, Tobramycin, SXT	Linezolid, Tigecycline
**MAB029**	Smooth	Sputum	N/A	MDR	Cefoxitin, Ciprofloxacin, Clarithromycin, Doxycycline, Imipenem, Minocycline, Moxifloxacin, Tobramycin, SXT	Amikacin, Linezolid, Tigecycline
**MAB035**	Smooth	Bronchial Wash	N/A	MDR	Cefoxitin, Ciprofloxacin, Clarithromycin, Doxycycline, Imipenem, Linezolid, Minocycline, Moxifloxacin, Tobramycin, SXT	Amikacin, Tigecycline
**MAB027**	Smooth	Sputum	N/A	MDR	Amikacin, Cefoxitin, Ciprofloxacin, Clarithromycin, Doxycycline, Imipenem,	Tigecycline

					Linezolid, Moxifloxacin, Tobramycin, SXT	
**MAB041**	Rough	Sputum	N/A	MDR	Cefoxitin, Ciprofloxacin, Doxycycline, Imipenem, Linezolid, Minocycline, Moxifloxacin, Tobramycin, SXT	Amikacin, Clarithromycin, Tigecycline
**MAB048**	Smooth	N/A	N/A	MDR	Amikacin, Ciprofloxacin, Doxycycline, Imipenem, Minocycline, Moxifloxacin, Tobramycin, SXT	Cefoxitin, Clarithromycin, Linezolid, Tigecycline
**MAB051**	Smooth	Bronchial Wash	N/A	MDR	Amikacin, Cefoxitin, Ciprofloxacin, Doxycycline, Imipenem, Linezolid, Minocycline, Moxifloxacin, Tobramycin, SXT	Tigecycline
**MAB052**	Smooth	Bronchial Wash	M300I	MDR	Cefoxitin, Ciprofloxacin, Clarithromycin, Doxycycline, Imipenem, Minocycline, Moxifloxacin,	Amikacin, Linezolid, Tigecycline

					Tobramycin, SXT	
**MAB053**	Rough	Sputum	P163R	MDR	Amikacin, Cefoxitin, Ciprofloxacin, Doxycycline, Imipenem, Linezolid, Minocycline, Moxifloxacin, Tobramycin, SXT	Tigecycline
**MAB056**	Smooth	Skin	N/A	MDR	Amikacin, Cefoxitin, Ciprofloxacin, Doxycycline, Imipenem, Linezolid, Minocycline, Moxifloxacin, Tobramycin, SXT	Tigecycline
**MAB057**	Smooth	N/A	M300I	MDR	Cefoxitin, Ciprofloxacin, Clarithromycin, Doxycycline, Imipenem, Minocycline, Moxifloxacin, Tobramycin, SXT	Amikacin, Linezolid, Tigecycline
**MAB058**	Smooth	Sputum	N/A	MDR	Cefoxitin, Ciprofloxacin, Clarithromycin, Doxycycline, Imipenem, Linezolid, Minocycline, Moxifloxacin,	Tigecycline

					Tobramycin, SXT	
**MAB086**	Smooth	Sputum	N/A	MDR	Cefoxitin, Ciprofloxacin, Clarithromycin, Doxycycline, Imipenem, Linezolid, Minocycline, Moxifloxacin, Tobramycin, SXT	Tigecycline
**MAB088**	Rough	Ear	N/A	MDR	Cefoxitin, Ciprofloxacin, Clarithromycin, Doxycycline, Imipenem, Linezolid, Minocycline, Moxifloxacin, Tobramycin, SXT	Amikacin, Tigecycline
**MAB089**	Smooth	Arm	N/A	MDR	Cefoxitin, Ciprofloxacin, Clarithromycin, Doxycycline, Imipenem, Linezolid, Minocycline, Moxifloxacin, Tobramycin, SXT	Amikacin, Tigecycline

Table S4: EC50, MIC and AUC values of the 30 clinical isolates and the lab strain *M. abscessus* ATCC 19977, treated with the panel of 7 MmpL3 inhibitors. The EC50 and MIC values are presented in µM. N/A indicates an inability to calculate MIC due to a lack of a sigmoidal dose-response curve.

	MSU-43085			MSU-43644			MSU-44147			MSU-43557			MSU-45518			MSU-45431			MSU-45683		
Isolate	EC50	MIC	AUC	EC50	MIC	AUC	EC50	MIC	AUC	EC50	MIC	AUC	EC50	MIC	AUC	EC50	MIC	AUC	EC50	MIC	AUC
Mab048	3.68	37.69	132.50	20.30	562.67	117.30	1.12	10.62	188.80	1.71	13.23	173.40	4.96	19.40	131.70	0.63	0.97	209.10	5.02	22.23	135.20
Mab051	3.37	17.01	137.70	1.17	2.43	175.90	0.67	1.33	197.70	1.29	3.43	177.80	0.92	1.88	162.10	0.28	0.40	247.30	0.56	1.13	196.70
Mab052	4.87	9.89	119.20	1.21	2.17	178.60	0.83	1.89	197.90	1.51	3.02	175.10	0.81	2.37	180.40	0.27	0.34	246.20	0.39	0.81	227.60
Mab056	2.89	5.44	130.20	8.41	19.75	102.40	2.48	3.97	151.10	1.71	5.62	166.80	6.77	9.24	106.10	0.59	0.85	209.90	7.28	9.74	109.40
Mab053	1.85	8.42	147.30	10.05	34.30	98.32	2.54	4.18	149.40	1.12	4.28	175.40	4.67	28.67	127.50	0.73	0.83	200.70	6.10	15.78	117.90
Mab088	3.71	8.34	149.10	1.36	2.62	200.80	0.80	1.65	229.30	1.58	4.61	196.20	1.36	4.68	194.20	0.29	0.45	286.20	0.68	1.52	242.20
Mab058	>80	N/A	32.50	20.61	60.38	68.85	6.03	112.72	96.17	14.21	15.71	45.04	3.05	7.17	65.12	2.31	30.58	117.70	2.57	20.65	95.78
Mab089	10.81	49.82	67.19	6.35	46.70	106.80	2.94	6.67	115.90	1.42	6.11	152.60	4.64	9.06	77.71	1.00	2.49	167.50	3.28	8.95	116.90
Mab086	7.17	15.12	95.43	1.12	2.15	164.00	0.71	1.06	184.10	1.92	3.42	156.00	0.88	0.99	156.10	0.35	0.94	229.70	0.42	0.86	212.70
Mab016	4.32	35.57	127.40	7.07	10.37	103.30	2.72	5.49	147.30	1.92	8.53	160.50	4.82	7.87	123.10	0.58	1.11	199.20	6.65	9.34	110.00
Mab035	4.72	15.59	105.90	12.39	14.48	88.45	2.61	7.23	147.40	1.93	8.58	152.10	5.12	18.01	111.90	0.57	1.10	193.40	6.82	11.00	110.70
Mab057	2.44	10.02	143.60	7.00	15.04	113.50	1.49	5.23	174.00	1.37	2.49	174.70	2.75	6.56	133.10	0.71	0.80	218.80	6.21	14.13	123.20
Mab026	5.41	10.14	124.60	1.55	2.32	178.20	0.68	1.20	213.90	1.44	3.17	182.10	1.21	2.01	169.70	0.31	0.68	266.90	0.57	1.12	221.10
Mab011	2.59	15.87	140.00	6.06	11.36	119.50	2.66	4.58	156.30	1.78	5.73	160.30	5.16	42.17	134.30	0.57	0.89	209.70	5.63	37.30	136.60
Mab015	3.34	20.42	134.60	6.38	10.16	115.20	3.99	6.82	132.50	1.52	4.58	172.90	5.73	6.62	115.00	0.65	0.81	216.80	6.54	8.48	110.50
Mab014	2.89	5.15	123.50	10.32	33.91	100.50	2.34	2.71	141.90	1.42	2.42	162.30	3.46	7.22	129.60	0.77	0.88	197.10	4.91	16.74	117.40
Mab004	>80	N/A	25.01	>80	N/A	27.30	>80	N/A	35.24	>80	N/A	21.18	>80	N/A	19.06	>80	N/A	38.94	>80	N/A	29.27
Mab028	18.23	349.58	86.55	13.62	233.95	104.30	2.99	12.59	127.30	4.01	26.64	127.70	6.03	8.85	92.33	0.63	1.04	129.60	6.18	33.82	109.10
Mab019	5.53	13.85	121.00	1.96	6.14	162.80	0.88	2.88	198.20	1.77	4.83	165.00	1.74	6.63	163.10	0.41	0.87	227.10	3.06	9.47	155.00
Mab022	4.70	14.60	108.80	1.93	6.62	146.50	1.04	2.58	170.60	2.83	8.74	140.20	2.18	8.07	151.20	0.37	0.93	219.10	0.91	3.59	185.40
Mab029	2.01	4.96	142.00	8.33	13.48	105.00	4.04	8.77	133.90	1.47	5.79	169.60	5.51	20.40	124.40	0.49	0.73	214.20	5.33	8.61	119.10
Mab002	3.00	18.53	131.30	6.15	22.70	125.60	1.62	4.24	173.90	2.51	11.01	157.00	2.39	11.65	152.60	0.61	1.24	208.60	5.79	16.46	126.90
Mab037	>80	N/A	61.53	>80	N/A	58.05	>80	N/A	25.00	>80	N/A	103.00	>80	N/A	76.56	>80	N/A	43.13	>80	N/A	41.26
Mab041	4.75	5.55	108.10	8.76	37.75	102.60	1.41	5.10	172.40	3.53	8.04	136.00	6.74	10.25	106.30	0.75	1.07	196.40	6.47	12.18	115.00
Mab010	2.67	45.21	138.50	1.24	5.40	186.80	1.40	2.74	184.70	2.79	15.95	159.70	1.25	3.47	168.50	0.58	3.28	206.90	0.80	2.14	206.40
Mab006	1.62	5.90	165.10	8.02	71.53	120.20	1.19	3.55	173.80	1.57	7.17	169.70	2.46	9.59	146.00	0.53	1.02	202.50	2.43	10.53	147.40
Mab018	2.48	5.56	134.10	6.15	17.61	117.80	3.54	9.90	140.70	2.97	6.23	153.80	6.55	8.83	109.50	0.45	0.98	223.40	5.67	9.25	118.60
Mab023	>80	N/A	32.44	>80	N/A	16.89	>80	N/A	30.63	>80	N/A	39.43	>80	N/A	98.73	>80	N/A	107.80	>80	N/A	46.70
Mab025	>80	N/A	43.15	>80	N/A	65.62	>80	N/A	93.51	>80	N/A	58.71	>80	N/A	40.88	>80	N/A	25.54	>80	N/A	100.60
Mab007	1.54	24.84	154.70	1.72	9.12	164.20	2.81	3.50	132.10	2.99	12.26	115.80	1.65	6.53	119.70	0.34	1.56	208.70	0.59	67.31	184.30
Mab ATCC_19977	4.76	10.55	141.00	0.47	9.15	251.80	0.42	8.15	262.10	1.96	3.69	250.30	0.13	16.93	307.00	0.19	1.28	282.70	0.23	3.79	271.30

Table S5: Summary of 20 antimycobacterial agents used in pair-wise drug interaction studies, including mechanisms of action and potencies against *M. abscessus* ATCC 19977.

Antimycobacterial Agent	Abbreviation	Drug Class	Mechanism of Action/Target	EC50 (µM)
**MSU-43085**	N/A	MmpL3 Inhibitor	MmpL3 function inhibition	4.76
**MSU-43644**	N/A			0.44
**MSU-44147**	N/A			0.46
**MSU-43557**	N/A			1.29
**MSU-45518**	N/A			0.15
**MSU-45431**	N/A			0.20
**MSU-45683**	N/A			0.21
**Amikacin**	AMK	Aminoglycoside	Protein synthesis inhibition (30S ribosomal subunit). Krause et al.[3]	2.40
**Kanamycin**	KAN			16.70
**Clarithromycin**	CLR	Macrolide	Protein synthesis inhibition (50S ribosomal subunit). Parnham et al.[4]	0.59
**Linezolid**	LNZ	Oxazolidinones	Protein synthesis inhibition (23S of the 50S ribosomal subunit) Hashemian et al.[5]	6.00
**Meropenem**	MER	β-lactam antibiotic	Peptidoglycan cross-linking inhibition (Penicillin-binding proteins inhibition) i.e., L, D-transpeptidase. Zandi & Townsend [6]	26.00
**Ethambutol**	EMB	ethylenediamine derivative	Inhibits Mycolic acid attachment to 5' OH of D-arabinose in the	20.00

			arabinogalactan layer of the cell wall (Arabinosyltransferases i.e., EmbB). Zhang et al.[7]	
**Rifampin**	RIF	Ansamycins	Inhibit Transcription (RNA Polymerase). Johansen et al.[8]	20.00
**Rifabutin**	RifU			0.14
**Moxifloxacin**	MOX	Fluoroquinolones	Reduces genomic integrity by inhibiting DNA gyrase. Aldred et al.[9]	0.70
**Ciprofloxacin**	CFLX			5.90
**Bedaquiline**	BDQ	Diarylquinoline	Inhibits ATP synthesis (ATP Synthase). Sarathy et al.[10]	0.19
**Clofazimine**	CLZ	Riminophenazine	Unclear: ROS generation and DNA intercalation/binding? McGuffin et al. [11]	0.15
**HC-2210**	N/A	Nitro-furans	Prodrugs: Release nitric oxide to Inhibit Arabinogalactan synthesis (DprE2) Eke et al.[12]	0.50

Table S6: A matrix of FIC_2 values of 210 pair-wise combinations of test compounds and standard-of-care treatments. The matrix shows that different combinations exhibit additive (FIC_2 of 0.8 to 1.2), synergistic ($FIC_2 < 0.8$) , and antagonistic ($FIC_2 > 1.2$) interactions. The most synergistic interactions are reported between ciprofloxacin and kanamycin or clarithromycin, while the most antagonistic interactions are reported between Amikacin and both Meropenem and ethambutol as well as Linezolid with HC-2210, a nitro containing compound developed by our lab with collaborators.

	MSU-43085	MSU-43644	MSU-44147	MSU-43557	MSU-45518	MSU-45431	MSU-45683	KAN	CFLX	BDQ	AMK	CLZ	RifU	LNZ	MER	EMB	MOX	RIF	CLR	HC-2210
**MSU-43085**	1.00	1.01	1.01	1.16	1.05	1.02	1.01	1.46	0.60	0.93	0.88	0.96	0.85	1.13	0.89	1.01	1.02	1.02	0.73	0.96
**MSU-43644**	1.01	1.00	0.96	1.04	1.01	1.02	0.94	1.46	0.53	0.86	0.54	0.72	0.90	1.02	0.62	0.97	0.89	1.02	0.85	0.97
**MSU-44147**	1.01	0.96	1.00	1.03	1.06	1.00	0.74	1.91	0.89	0.85	1.06	0.69	0.88	1.00	0.89	0.89	0.84	0.94	0.95	0.96
**MSU-43557**	1.16	1.04	1.03	1.00	1.03	1.00	1.00	2.27	0.76	0.81	0.88	0.94	0.76	0.91	0.73	1.02	1.11	0.96	0.91	0.94
**MSU-45518**	1.05	1.01	1.06	1.03	1.00	1.02	1.10	1.91	1.05	0.83	0.84	0.96	0.78	1.09	0.92	0.67	0.87	0.72	0.90	0.96
**MSU-45431**	1.02	1.02	1.00	1.00	1.02	1.00	1.19	1.59	0.99	0.91	0.92	0.83	0.72	0.93	0.85	0.97	0.97	1.05	0.54	0.42
**MSU-45683**	1.01	0.94	0.74	1.00	1.10	1.19	1.00	1.56	0.76	0.81	0.95	0.62	0.76	0.91	1.02	1.02	1.21	0.96	0.75	0.88
**KAN**	1.46	1.46	1.91	2.27	1.91	1.59	1.56	1.00	0.29	1.00	1.10	0.69	0.97	0.91	0.80	1.23	2.16	0.86	0.44	0.98
**CFLX**	0.60	0.53	0.89	0.76	1.05	0.99	0.76	0.29	1.00	2.35	1.03	1.00	1.13	1.18	0.54	0.54	1.44	0.72	0.33	0.84
**BDQ**	0.93	0.86	0.85	0.81	0.83	0.91	0.81	1.00	2.35	1.00	0.77	0.82	0.75	0.90	2.33	0.90	1.41	1.05	0.46	0.85
**AMK**	0.88	0.54	1.06	0.88	0.84	0.92	0.95	1.10	1.03	0.77	1.00	0.76	0.89	0.88	0.83	2.50	2.40	0.93	1.00	1.03
**CLZ**	0.96	0.72	0.69	0.94	0.96	0.83	0.62	0.69	1.00	0.82	0.76	1.00	0.89	0.96	1.01	1.01	0.84	0.90	0.83	0.93
**RifU**	0.85	0.90	0.88	0.76	0.78	0.72	0.76	0.97	1.13	0.75	0.89	0.89	1.00	0.86	0.85	2.47	0.99	0.89	0.49	0.89
**LNZ**	1.13	1.02	1.00	0.91	1.09	0.93	0.91	0.91	1.18	0.90	0.88	0.96	0.86	1.00	1.09	0.92	2.47	1.00	0.93	2.50
**MER**	0.89	0.62	0.89	0.73	0.92	0.85	1.02	0.80	0.54	2.33	0.83	1.01	0.85	1.09	1.00	0.70	0.78	0.63	0.83	0.85
**EMB**	1.01	0.97	0.89	1.02	0.67	0.97	1.02	1.23	0.54	0.90	2.50	1.01	2.47	0.92	0.70	1.00	0.78	0.85	1.01	1.01
**MOX**	1.02	0.89	0.84	1.11	0.87	0.97	1.11	2.16	1.44	1.41	2.40	0.84	0.99	2.47	0.78	0.78	1.00	2.44	1.02	2.41
**RIF**	1.02	1.02	0.94	0.96	0.72	1.05	0.96	0.86	0.72	1.05	0.93	0.90	0.89	1.00	0.63	0.85	2.44	1.00	0.96	0.97
**CLR**	0.73	0.85	0.95	0.91	0.90	0.54	0.75	0.44	0.33	0.46	1.00	0.83	0.49	0.93	0.83	1.01	1.02	0.96	1.00	0.94
**HC-2210**	0.96	0.97	0.96	0.94	0.96	0.42	0.88	0.98	0.84	0.85	1.03	0.93	0.89	2.50	0.85	1.01	2.41	0.97	0.94	1.00

Table S7. The breakdown of energy contributions (kcal/mol) of relevant hydrophilic binding pocket residues to ligand binding in the wild type and V299A mutant. Data are shown for a selected subset of the simulated ligands.

		TYR266	THR298	SER302	ASP625	TYR626
**Wild Type**	**MSU-45683**	-1.646	-0.0145	-1.464	7.299	-2.3425
**V299A**	**MSU-45683**	-1.008	0.2225	-0.6365	8.6035	-0.821
**Wild Type**	**MSU-45819**	-1.5565	0.482	-1.9175	15.5605	-3.7765
**V299A**	**MSU-45819**	-1.23	0.037	-0.7975	11.6355	-1.016
**Wild Type**	**MSU-46350**	-1.8375	0.239	-1.0365	8.6745	-4.152
**V299A**	**MSU-46350**	-1.964	0.7	-0.829	5.774	-2.5675
**Wild Type**	**MSU-43085**	-2.87	0.612	-2.045	13.313	-1.507
**V299A**	**MSU-43085**	-1.7185	0.012	-0.271	5.238	-2.115
**Wild Type**	**MSU-435557**	-1.2705	0.3125	-1.678	14.6175	-3.813

V299A	MSU-435557	-1.8485	0.5155	-0.9615	8.031	-3.0835

Table S8: Reagents, Resources, and Software used in the study

REAGENT OR RESOURCE	SOURCE	IDENTIFIER
**Bacterial and virus strains**		
M. tuberculosis (Erdman)	Dr. JoAnne L. Flynn	N/A
M. tuberculosis (CDC1551)	Dr. David Russell	N/A
M. tuberculosis (H37Rv)	Dr. David Russell	N/A
M. smegmatis (MC^2155)	Dr. David Russell	N/A
M. abscessus	ATCC	Cat# ATCC 19977
P. aeruginosa	ATCC	Cat# ATCC 27853
E. coli	ATCC	Cat# ATCC 25922
P. vulgaris	ATCC	Cat# ATCC 13315
S. aureus	ATCC	Cat# ATCC 29213
S. aureus	ATCC	Cat# ATCC 25923
E. faecalis	ATCC	Cat# ATCC 29212
M. abscessus pmV261 hsp60::mEmerald	Dr. Andrew Olive	N/A
M. abscessus clinical isolates	Dr. Allison F. Carey	N/A
**Chemicals, peptides, and recombinant proteins**		
MSU-43085	This Study	N/A
MSU-43644	This Study	N/A
MSU-44147	This Study	N/A
MSU-43557	This Study	N/A
MSU-45518	This Study	N/A
MSU-45431	This Study	N/A
MSU-45683	This Study	N/A
MSU-45655	This Study	N/A
MSU-43186	This Study	N/A
MSU-45540	This Study	N/A

MSU-45516	This Study	N/A
MSU-45538	This Study	N/A
MSU-45606	This Study	N/A
MSU-45819	This Study	N/A
MSU-45350	This Study	N/A
Zeocin	Thermo Scientific	Cat#R25001
Amikacin Disulfate	Sigma Aldrich	A1774, 39831-55-5
DMSO	Sigma Aldrich	67-68-5
CCCP	Sigma Aldrich	C2759
$DioC_2$	Invitrogen	D14730
Tyloxapol	Sigma Aldrich	25301-02-4
Crystal violet	Sigma Aldrich	548-62-9
Resazurin (salt)	Sigma Aldrich	62758-13-8
Kanamycin Sulfate	Sigma Aldrich	25389-94-0
Linezolid	Sigma Aldrich	165800-03-3
Rifampin	Sigma Aldrich	13292-46-1
HC2210	This and previous Study [(12)]	N/A
Rifabutin	Sigma Aldrich	72559-06-9
Clofazimine	Sigma Aldrich	2030-63-9
Clarithromycin	Thermo Scientific	Cat#J66005
Azithromycin	Sigma Aldrich	CAS#117772-70-0
Tigecycline	Thermo Scientific	CAS#220620-09-7
Meropenem	Sigma Aldrich	CAS#119478-56-7
Ciprofloxacin	Sigma Aldrich	85721-33-1
Bedaquiline	Asta Tech	Cat#43211
Ethambutol Dihydrochloride	Sigma Aldrich	CAS#1070-11-7
Moxifloxacin	Sigma Aldrich	186826-86-8
Ethoxzolamide	Sigma Aldrich	452-35-7

Verapamil Hydrochloride	Sigma Aldrich	152-11-4
D-cycloserine	Sigma Aldrich	68-41-7
Trimethoprim	Sigma Aldrich	98 23256-42-0
Amoxicillin	Acros Organics	26787-78-0
Potassium Clavulanate	Sigma Aldrich	61177-45-5
Ampicillin- Sodium	Sigma Aldrich	171254
Streptomycin Sulfate	Sigma Aldrich	S9137
**Critical commercial assays**		
Cell Titer-Glo 2.0 assay kit	Promega	Cat#G7570
**Deposited data**		
RNA-seq Data Sup1	This Study	Accession number: GSE296524
**Experimental models: Cell lines**		
Mouse: Primary Bone marrow-derived macrophages	This Study	N/A
**Experimental models: Organisms/strains**		
Mouse: C57BL/6J	The Jackson Laboratory	Stock No: 000664
**Software and algorithms**		
GraphPad Prism 10	GraphPad software	https://www.graphpad.com/
MATLAB_R2024b	MATLAB software	https://www.mathworks.com/products/matlab.html
CLC Genomics Workbench	QIAGEN	https://www.qiagen.com Cat. No. / ID: 832021
OrthoVenn3 (13)	Sun et al., 2023	https://orthovenn3.bioinfotoolkits.net/home

Kyoto Encyclopedia of Genes and Genomes (KEGG)[14]	Kanehisa et al., 2015	https://www.genome.jp/kegg/pathway.html
Venny 2.1 [15]	Oliveros, C., 2015	https://bioinfogp.cnb.csic.es/tools/venny/
Pymol	Schrödinger	https://pymol.org/2/
Amber22	University of California San Francisco	https://ambermd.org/GetAmber.php
CCG MOE	Chemical Computing Group (CCG)	https://www.chemcomp.com/en/index.html
VMD	University of Illinois Urbana-Champaign	http://www.ks.uiuc.edu/Research/vmd/ RRID:SCR_024368

References:

1. Yang, T.; Beach, K. E.; Zhu, C.; Gan, M.; Wang, W.; Zhou, H.; Peng, L.; Wang, S.; Cai, L.; Li, W.; et al. Genomic analysis of global Mycobacterium abscessus isolates reveals ongoing evolution of drug-resistance-associated genes. J Infect Dis 2024. DOI: 10.1093/infdis/jiae580 From NLM Publisher.

2. Waites, K. B.; Bade, D. J.; Bebear, C.; Brown, S. D.; Davidson, M. K.; Duffy, L. B.; Kenny, G.; Matlow, A.; Shortridge, D.; Talkington, D.; et al. Methods for Antimicrobial Susceptibility Testing for Human Mycoplasmas; Approved Guideline; 2011.

3. Krause KM, Serio AW, Kane TR, Connolly LE. Aminoglycosides: An overview. Cold Spring Harbor Perspectives in Medicine. 2016 Jun;6(6). doi:10.1101/cshperspect.a027029

4. Parnham MJ, Haber VE, Giamarellos-Bourboulis EJ, Perletti G, Verleden GM, Vos R. Azithromycin: Mechanisms of action and their relevance for clinical applications. Pharmacology & Therapeutics. 2014 Aug;143(2):225–45. doi:10.1016/j.pharmthera.2014.03.003

5. Hashemian SM, Farhadi T, Ganjparvar M. Linezolid: A review of its properties, function, and use in critical care. Drug Design, Development and Therapy. 2018 Jun;Volume 12:1759–67. doi:10.2147/dddt.s164515

6. Zandi TA, Townsend CA. Competing off-loading mechanisms of meropenem from an L,d -transpeptidase reduce antibiotic effectiveness. Proceedings of the National Academy of Sciences. 2021 Jun 29;118(27). doi:10.1073/pnas.2008610118

7. Zhang L, Zhao Y, Gao Y, Wu L, Gao R, Zhang Q, et al. Structures of cell wall arabinosyltransferases with the anti-tuberculosis drug Ethambutol. Science. 2020 Jun 12;368(6496):1211–9. doi:10.1126/science.aba9102

8. Johansen MD, Daher W, Roquet-Banères F, Raynaud C, Alcaraz M, Maurer FP, et al. Rifabutin is bactericidal against intracellular and extracellular forms of mycobacterium abscessus. Antimicrobial Agents and Chemotherapy. 2020 Oct 20;64(11). doi:10.1128/aac.00363-20

9. Aldred KJ, Blower TR, Kerns RJ, Berger JM, Osheroff N. Fluoroquinolone interactions with *mycobacterium tuberculosis* gyrase: Enhancing drug activity against wild-type and resistant gyrase. Proceedings of the National Academy of Sciences. 2016 Jan 20;113(7). doi:10.1073/pnas.1525055113

10. Sarathy JP, Gruber G, Dick T. Re-understanding the mechanisms of action of the anti-mycobacterial drug Bedaquiline. Antibiotics. 2019 Dec 11;8(4):261. doi:10.3390/antibiotics8040261

11. McGuffin SA, Pottinger PS, Harnisch JP. Clofazimine in nontuberculous mycobacterial infections: A growing niche. Open Forum Infectious Diseases. 2017;4(3). doi:10.1093/ofid/ofx147

12. Eke IE, Williams JT, Haiderer ER, Albrecht VJ, Murdoch HM, Abdalla BJ, et al. Discovery and characterization of antimycobacterial nitro-containing compounds with distinct mechanisms of action and *in vivo* efficacy. Antimicrobial Agents and Chemotherapy. 2023 Sept 19;67(9). doi:10.1128/aac.00474-23

13. Oliveros, J.C. (2007-2015) Venny. An interactive tool for comparing lists with Venn's diagrams. https://bioinfogp.cnb.csic.es/tools/venny/index.html

14. Sun J, Lu F, Luo Y, Bie L, Xu L, Wang Y. Orthovenn3: An integrated platform for exploring and visualizing orthologous data across genomes. Nucleic Acids Research. 2023 Apr 28;51(W1). doi:10.1093/nar/gkad313

15. Kanehisa M, Sato Y, Kawashima M, Furumichi M, Tanabe M. Kegg as a reference resource for gene and protein annotation. Nucleic Acids Research. 2015 Oct 17;44(D1). doi:10.1093/nar/gkv1070
