# Supplementary material for: Defining the Mechanism of Action and Resistance of New Mycobacterium abscessus MmpL3 Inhibitors
Source: ACS Chem Biol. 2026 Jan 13;21(2):284–301. doi: 10.1021/acschembio.5c00709 (PMC12930390; doi:10.1021/acschembio.5c00709)
Supplement: Supplementary file 3 [file cb5c00709_si_003.pdf]

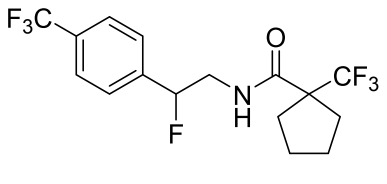

MSU-45683. ***N*-{2-fluoro-2-[4-(trifluoromethyl)phenyl]ethyl}-1-(trifluoromethyl)cyclopentane-1-carboxamide**. To a 50 mL round bottom flask containing a stir bar was added 1-trifluoromethyl-1-cyclopentyl carboxylic acid (0.180 g, 1.00 mmol). The reaction vessel was sealed, flushed with argon, and N,N-dimethylformamide (1.00 mL) was added *via* syringe, followed by triethylamine (0.202 g, 2.00 mmol) and O-(7-azabenzotriazol-1-yl)-N,N,N',N'-tetramethyluronium hexafluorophosphate **(**0.418 g, 1.10 mmol**).** In a separate vial, 2-Fluoro-2-[4-(trifluoromethyl) phenyl]ethanamine (0.249 g, 1.20 mmol) was dissolved in N,N-dimethylformamide (1.00 mL) and was added to the carboxylic acid containing solution by syringe. The reaction mixture was stirred for 20 hours at which time it was diluted with ethyl acetate, washed with water, 1.0 N aqueous hydrochloric acid, saturated aqueous sodium bicarbonate, and brine. The organic layer was dried dried over sodium sulfate, filtered and concentrated *in vacuo*. The crude material was purified by silica gel chromatography (0 – 10 % methanol in dichloromethane) to yield the final product (0.218 g, 59 % yield). ^{1}H NMR (500 MHz, $CDCl_3$) δ 7.66 (d, J = 8.1 Hz, 2H), 7.55 – 7.44 (m, 2H), 6.23 (s, 1H), 5.74 – 5.51 (m, 1H), 4.00 – 3.82 (m, 1H), 3.66 – 3.48 (m, 1H), 2.28 (dd, J = 13.2, 6.8 Hz, 1H), 2.25 – 2.17 (m, 1H), 2.07 – 1.92 (m, 2H), 1.82 – 1.60 (m, 4H). ^{19}F NMR (470 MHz, $CDCl_3$) δ -62.76, -70.35, -187.35 (ddd, J = 48.8, 29.7, 19.8 Hz). HRMS ESI (+) calc'd for [M+H] = 372.1194, found = 372.1215.

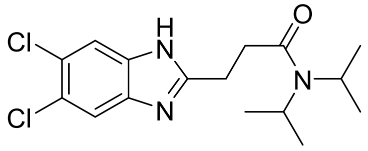

MSU-43085. 3-(5,6-dichloro-1*H*-1,3-benzimidazol-2-yl)-*N*,*N*-di(propan-2-yl)propanamide. To a 100 mL round bottom flask, containing a stir bar, was added 4,5-dichloro-1,2-diaminobenzene (0.531 g, 3.00 mmol), 4-[di(propan-2-yl)amino]-4-oxobutanoic acid (0.804 g, 4.00 mmol) and 1,4-dioxane (10.0 mL). The reaction mixture was stirred until all solids dissolved and polyphosphoric acid (2.00 g, 24.0 mmol) was added. The reaction vessel was sealed, flushed with argon, and heated to 110 ^{o} C for 19 hours, at which time it was cooled to room temperature, diluted with ethyl acetate and water and neutralized with sodium carbonate. The layers were partitioned, the organic layer was washed with water and brine, dried over sodium sulfate, filtered and concentrated *in vacuo*. The residue was dissolved in methanol and purified by reverse phase medium pressure liquid chromatography (0 – 100 % methanol in 25 mM aqueous ammonium formate). Fractions containing product were combined, concentrated *in vacuo*, dissolved in ethyl acetate, washed with saturated aqueous sodium bicarbonate, brine, dried over sodium sulfate, filtered and concentrated *in vacuo*. This material was further purified by silica gel chromatography

(0 – 10 % methanol in dichloromethane) to yield the final product (0.507 g, 50 % yield). ^{1}H NMR (500 MHz, DMSO-d_6) δ 12.49 (s, 1H), 7.72 (s, 2H), 4.04 (hept, J = 6.8 Hz, 1H), 3.50 (s, 1H), 3.00 (dd, J = 8.5, 6.4 Hz, 2H), 2.82 (dd, J = 8.5, 6.4 Hz, 2H), 1.25 (d, J = 6.7 Hz, 6H), 1.13 (d, J = 6.6 Hz, 6H). APCI [M+H] calc'd = 342.1134 observed = 342.1168.

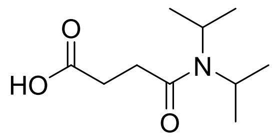

4-[di(propan-2-yl)amino]-4-oxobutanoic acid. To a 100 mL round bottom flask containing a stir bar was added ethyl acetate (50.0 mL). Succinic anhydride was added as a solid (1.00 g, 10.0 mmol) and the reaction vessel was sealed, flushed with argon, and heated to 50 º C. Diisopropylamine (1.11 g, 11.0 mmol) was added *via* syringe. The reaction mixture was stirred for 22 hours, cooled to room temperature and concentrated *in vacuo* (2.01 g, 100 % yield). This material was used without further purification.

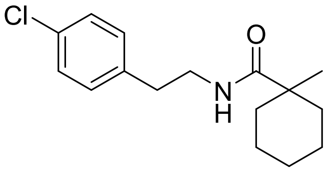

MSU-43644. ***N*-[2-(4-chlorophenyl)ethyl]-1-methylcyclohexane-1-carboxamide**. To a 50 mL round bottom flask containing a stir bar was added 1-methylcyclohexane-1- carboxylic acid (0.284 g, 2.00 mmol). The reaction vessel was sealed, flushed with argon, and N,N-dimethylformamide (3.00 mL) was added *via* syringe, followed by triethylamine (0.404 g, 4.00 mmol) and O-(7-azabenzotriazol-1-yl)-N,N,N',N'-tetramethyluronium hexafluorophosphate (0.836 g, 2.20 mmol). In a separate vial 2-(4-chlorophenyl)ethylamine (0.373 g, 2.40 mmol) was dissolved in N,N-dimethylformamide (1.00 mL) and was added to the carboxylic acid containing solution by syringe. The reaction mixture was stirred for 20 hours at which time it was diluted with ethyl acetate, washed with water, 1.0 N aqueous hydrochloric acid, saturated aqueous sodium bicarbonate, and brine. The organic layer is dried over sodium sulfate, filtered and concentrated *in vacuo*. The crude material is purified by silica gel chromatography (0 – 100 % ethyl acetate in hexanes) and further purified by trituration from dichloromethane and hexanes to yield the final product (0.145 g, 26 % yield). ^{1}H NMR (500 MHz, $CDCl_3$) δ 7.32 – 7.25 (m, 2H), 7.16 – 7.11 (m, 2H), 5.62 (s, 1H), 3.50 (td, J = 6.9, 5.8 Hz, 2H), 2.80 (t, J = 6.9 Hz, 2H), 1.86 – 1.77 (m, 2H), 1.55 – 1.42 (m, 3H), 1.31 (dtd, J = 32.1, 10.9, 9.2, 3.2 Hz, 5H), 1.08 (s, 3H). HRMS ESI (+) Calc'd for [M+H] = 280.1464, found = 280.1474.

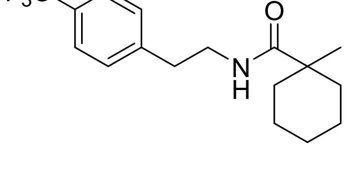

MSU-44147. 1-methyl-*N*-{2-[4-(trifluoromethyl)phenyl]ethyl}cyclohexane-1-carboxamide. To a 50 mL round bottom flask containing a stir bar was added 1-methylcyclohexane-1- carboxylic acid (0.241 g, 1.70 mmol). The reaction vessel was sealed, flushed with argon, and N,N-dimethylformamide (2.00 mL) was added *via* syringe, followed by triethylamine (0.404 g, 4.00 mmol) and O-(7-azabenzotriazol-1-yl)-N,N,N',N'-tetramethyluronium hexafluorophosphate (0.684 g, 1.80 mmol). In a separate vial 2-(4-trifluoromethylphenyl)ethylamine (0.378 g, 2.00 mmol) was dissolved in N,N-dimethylformamide (2.00 mL) and was added to the carboxylic acid containing solution by syringe. The reaction mixture was stirred for 20 hours at which time it was diluted with ethyl acetate, washed with water, 1.0 N aqueous hydrochloric acid, saturated aqueous sodium bicarbonate, and brine. The organic layer is dried over sodium sulfate, filtered and concentrated *in vacuo*. The crude material is purified by silica gel chromatography (0 – 10 % methanol in dichloromethane) and (0.035 g, 7 % yield). ^{1}H NMR (500 MHz, $CDCl_3$) δ 7.61 – 7.53 (m, 2H), 7.36 – 7.29 (m, 2H), 5.66 (s, 1H), 3.55 (td, J = 7.0, 5.9 Hz, 2H), 2.90 (t, J = 7.0 Hz, 2H), 1.88 – 1.76 (m, 2H), 1.56 – 1.45 (m, 2H), 1.45 – 1.39 (m, 1H), 1.39 – 1.23 (m, 5H), 1.09 (s, 3H). HRMS ESI (+) Calc'd for [M+H] = 314.1727, found = 314.1736.

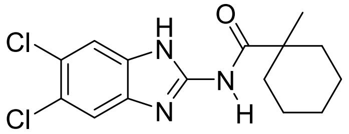

MSU-43557. *N*-(5,6-dichloro-1*H*-1,3-benzimidazol-2-yl)-1-methylcyclohexane-1-carboxamide. To a 50 mL round bottom flask containing a stir bar was added 1-methylcyclohexane-1- carboxylic acid (0.142 g, 1.00 mmol). The reaction vessel was sealed, flushed with argon, and N,N-dimethylformamide (1.00 mL) was added *via* syringe, followed by triethylamine (0.303 g, 3.00 mmol) and O-(7-azabenzotriazol-1-yl)-N,N,N',N'-tetramethyluronium hexafluorophosphate (0.418 g, 1.10 mmol). In a separate vial 5,6-dichlorobenzo[d]-imidazol-2-amine (0.242 g, 1.20 mmol) was dissolved in N,N-dimethylformamide (1.00 mL) and was added to the carboxylic acid containing solution by syringe. The reaction mixture was stirred for 20 hours, at which time it was diluted with ethyl acetate, washed with water, saturated aqueous sodium bicarbonate, and brine. The organic layer is dried over sodium sulfate, filtered and concentrated *in vacuo*. This material was purified by methanol and purified by reverse phase medium pressure liquid chromatography (0 – 100 % methanol in 25 mM aqueous ammonium formate). Fractions containing product were concentrated *in vacuo*, dissolved in ethyl acetate, washed with saturated aqueous sodium bicarbonate, brine, dried over sodium sulfate, filtered and concentrated *in vacuo*. This material was recrystallized from dichloromethane and hexanes to give the final product (0.069 g, 21 % yield). ^{1}H NMR (500 MHz, DMSO-d6) δ 12.39 (s, 1H), 11.29 (s, 1H), 7.62 (d, J = 9.1 Hz, 2H), 2.12 (d, J = 11.7 Hz, 2H), 1.56 – 1.25 (m, 8H), 1.21 (s, 3H). HRMS ESI (+) Calc'd for [M+H] = 326.0823, found = 326.0838.

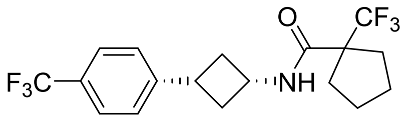

MSU-45518. 1-(trifluoromethyl)-*N*-{3-[4-(trifluoromethyl)phenyl]cyclobutyl}cyclopentane-1-carboxamide. To a 50 mL round bottom flask containing a stir bar was added 1-trifluoromethyl-1-cyclopently carboxylic acid (0.163 g, 0.900 mmol). The reaction vessel was sealed, flushed with argon, and N,N-dimethylformamide (1.00 mL) was added *via* syringe, followed by triethylamine (0.303 g, 3.00 mmol) and O-(7-azabenzotriazol-1-yl)-N,N,N',N'-tetramethyluronium hexafluorophosphate (0.361 g, 0.950 mmol). In a separate vial 3-[4-(trifluoromethyl)phenyl]cyclobutanamine (0.222 g, 1.03 mmol) was dissolved in N,N-dimethylformamide (0.200 mL) and was added to the carboxylic acid containing solution by syringe. The reaction mixture was stirred for 19 hours, then diluted with ethyl acetate, washed with water, 1.0 N aqueous hydrochloric acid, saturated aqueous sodium bicarbonate, and brine. The organic layer was dried over sodium sulfate, filtered and concentrated *in vacuo*. The crude material is purified by silica gel chromatography (0 – 10 % methanol in dichloromethane) to yield the final product (0.194 g, 57 % yield). ^{1}H NMR (500 MHz, $CDCl_3$) δ 7.56 (d, J = 8.0 Hz, 2H), 7.30 (d, J = 8.0 Hz, 2H), 5.92 (s, 1H), 4.41 (ddt, J = 16.5, 9.2, 7.3 Hz, 1H), 3.37 – 3.25 (m, 1H), 2.88 (dtd, J = 8.6, 7.5, 2.8 Hz, 2H), 2.27 (dt, J = 13.5, 5.7 Hz, 2H), 2.05 – 1.93 (m, 4H), 1.73 (td, J = 8.5, 4.6 Hz, 4H). ^{19}F NMR (470 MHz, $CDCl_3$) δ -62.37, -70.29. HRMS ESI (+) calc'd for [M+H] = 380.1445, found = 380.1474.

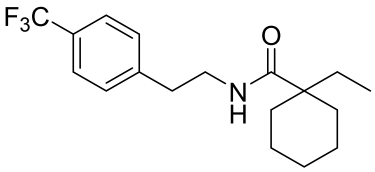

MSU-45431. 1-ethyl-*N*-{2-[4-(trifluoromethyl)phenyl]ethyl}cyclohexane-1-carboxamide. To a 50 mL round bottom flask containing a stir bar was added 1-ethyl-1-cyclohexyl carboxylic acid (0.119 g, 0.760 mmol). The reaction vessel was sealed, flushed with argon, and N,N-dimethylformamide (1.00 mL) was added *via* syringe, followed by triethylamine (0.202 g, 2.00 mmol) and O-(7-azabenzotriazol-1-yl)-N,N,N',N'-tetramethyluronium hexafluorophosphate (0.304 g, 0.800 mmol). In a separate vial 4-(2-trifluoromethyl)phenylethylamine (0.189 g, 1.00 mmol) was dissolved in N,N-dimethylformamide (1.00 mL) and was added to the carboxylic acid containing solution by syringe. The reaction mixture was stirred for 22 hours at which time it was diluted with ethyl acetate, washed with water, 1.0 N aqueous hydrochloric acid, saturated aqueous sodium bicarbonate, and brine. The organic layer is dried over sodium sulfate, filtered and concentrated *in vacuo*. The crude material is purified by silica gel chromatography (0 – 10 % methanol in dichloromethane) and (0.037 g, 15 % yield). ^{1}H NMR (500 MHz, $CDCl_3$) δ 7.59 –

7.54 (m, 2H), 7.33 (d, $J = 7.9$ Hz, 2H), 5.63 (s, 1H), 3.58 (td, $J = 7.0$, 5.9 Hz, 2H), 2.90 (t, $J = 7.0$ Hz, 2H), 1.89 – 1.79 (m, 2H), 1.44 (q, $J = 7.6$ Hz, 5H), 1.27 (q, $J = 10.9$ Hz, 5H), 0.74 (t, $J = 7.5$ Hz, 3H). ^{19}F NMR (470 MHz, $CDCl_3$) δ -62.44. HRMS ESI (+) calc'd for [M+Na] = 350.1708, found = 350.1745.

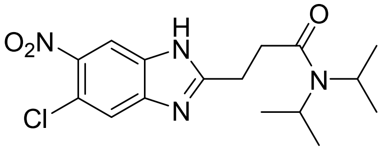

MSU-45655. 3-(5-chloro-6-nitro-1*H*-1,3-benzimidazol-2-yl)-*N*,*N*-di(propan-2-yl)propanamide. To a 100 mL round bottom flask containing a stir bar was added 4-Chloro-5-nitrobenzene-1,2-diamine (0.486 g, 2.50 mmol), 4-[di(propan-2-yl)amino]-4-oxobutanoic acid (0.593 g, 2.90 mmol), 1,4-dioxane (10.0 mL) and polyphosphoric acid (2.02 g, 24.0 mmol). The reaction vessel was sealed with a septum, flushed with argon, and heated to 110 º C for 22 hours. The reaction mixture was cooled to room temperature, diluted with ethyl acetate and water and neutralized with sodium carbonate. The organic layer was extracted with water and washed with brine, dried over sodium sulfate, filtered and concentrated *in vacuo*. This material was purified by methanol and purified by reverse phase medium pressure liquid chromatography (0 – 100 % methanol in 25 mM aqueous ammonium formate). Fractions containing product were combined, concentrated *in vacuo*, dissolved in ethyl acetate, washed with saturated aqueous sodium bicarbonate, brine, concentrated *in vacuo* and purified by silica gel chromatography (0 – 10 % methanol in dichloromethane) to yield the final product (0.564 g, 64 % yield). ^{1}H NMR (500 MHz, DMSO-d_6) δ 13.01 – 12.76 (m, 1H), 8.29 – 8.18 (m, 1H), 7.91 – 7.67 (m, 1H), 4.03 (p, $J = 6.6$ Hz, 1H), 3.45 (s, 1H), 3.04 (s, 2H), 2.84 (dd, $J = 8.1$, 6.6 Hz, 2H), 1.24 (d, $J = 6.7$ Hz, 6H), 1.13 (d, $J = 6.6$ Hz, 6H). HRMS ESI (+) calc'd for [M+H] = 353.1376, found = 353.1403.

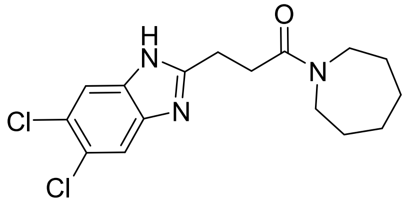

MSU-43186. 1-(azepan-1-yl)-3-(5,6-dichloro-1*H*-1,3-benzimidazol-2-yl)propan-1-one. To a 100 mL round bottom flask, containing a stir bar, was added 4,5-Dichlorobenzene-1,2-diamine (0.531 g, 3.00 mmol), 4-(azepan-1-yl)-4-oxobutanoic acid (0.797 g, 4.00 mmol), 1,4-dioxane (10.0 mL) and polyphosphoric acid (2.87 g, 30.0 mmol). The reaction vessel was sealed with a septum, flushed with argon, and heated to 110 º C for 20 hours. The reaction mixture was cooled to room temperature, diluted with ethyl acetate and water and neutralized with sodium carbonate. The organic layer was washed with water and washed with brine, dried over sodium sulfate, filtered and concentrated *in vacuo*. This material was purified by reverse phase medium pressure liquid chromatography (0 – 100 % methanol in 25 mM aqueous ammonium formate). Fractions

containing product were combined, concentrated *in vacuo*, dissolved in ethyl acetate, washed with saturated aqueous sodium bicarbonate, brine, concentrated *in vacuo* and purified by silica gel chromatography (0 – 10 % methanol in dichloromethane) to yield the final product (0.474 g, 47 % yield). ^{1}H NMR (500 MHz, DMSO-d_6) δ 12.49 (s, 1H), 7.71 (d, J = 32.6 Hz, 2H), 3.45 (t, J = 6.1 Hz, 2H), 3.41 – 3.37 (m, 2H), 3.03 (t, J = 7.2 Hz, 2H), 2.85 (t, J = 7.3 Hz, 2H), 1.67 (p, J = 5.9 Hz, 2H), 1.58 – 1.52 (m, 2H), 1.52 – 1.40 (m, 4H). HRMS ESI (+) calc'd for [M+H] = 340.0979, found = 340.0977.

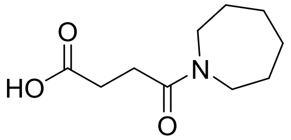

4-(azepan-1-yl)-4-oxobutanoic acid. To a 100 mL round bottom flask containing a stir bar was added ethyl acetate (50.0 mL). Maleic anhydride was added as a solid (1.00 g, 10.0 mmol) and the reaction vessel was sealed, flushed with argon, and heated to 50 º C. Cycloheptylimine (1.00 g, 11.0 mmol) was added via syringe. The reaction mixture was stirred for 22 hours, cooled to room temperature and concentrated *in vacuo* (2.00 g, 100 % yield). This material was used in the next step without further purification.

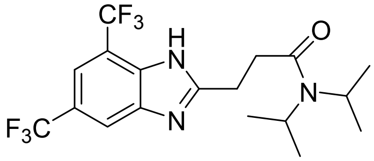

MSU-45540. 3-[5,7-bis(trifluoromethyl)-1*H*-1,3-benzimidazol-2-yl]-*N*,*N*-di(propan-2-yl)propanamide. To a 100 mL round bottom flask, containing a stir bar, was added 3,5-(Bistriufluoromethyl)benzene-1,2-diamine (0.244 g, 1.00 mmol), 4-[di(propan-2-yl)amino]-4-oxobutanoic acid (0.366 g, 1.80 mmol), 1,4-dioxane (10.0 mL) and polyphosphoric acid (1.73 g, 20.5 mmol). The reaction vessel was sealed with a septum, flushed with argon, and heated to 110 º C for 20 hours. The reaction mixture was cooled to room temperature, diluted with ethyl acetate and water and neutralized with sodium carbonate. The organic layer was extracted with water and washed with brine, dried over sodium sulfate, filtered and concentrated *in vacuo*. This material was purified by methanol and purified by reverse phase medium pressure liquid chromatography (0 – 100 % methanol in 25 mM aqueous ammonium formate). Fractions containing product were combined, concentrated *in vacuo*, dissolved in ethyl acetate, washed with saturated aqueous sodium bicarbonate, brine, concentrated *in vacuo* and purified by silica gel chromatography (0 – 10 % methanol in dichloromethane) to yield the final product (0.238 g, 58 % yield). ^{1}H NMR (500 MHz, DMSO-d_6) δ 13.11 (s, 1H), 8.29 – 7.94 (m, 1H), 7.83 – 7.61 (m, 1H), 4.04 (p, J = 6.6 Hz, 1H), 3.45 (s, 1H), 3.11 (td, J = 7.6, 3.3 Hz, 2H), 2.86 (q, J = 7.9 Hz, 2H), 1.22 (dd, J = 17.3, 6.7 Hz, 6H), 1.13 (d, J = 6.6 Hz, 6H). ^{19}F NMR (470 MHz, DMSO-d_6) δ -59.00, -59.05, -59.86, -60.02. HRMS ESI (+) calc'd for [M+H] = 410.1664, found = 410.1676.

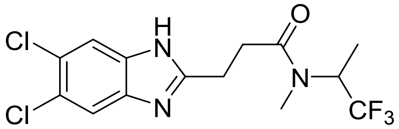

MSU-45516. 3-(5,6-dichloro-1*H*-1,3-benzimidazol-2-yl)-*N*-methyl-*N*-(1,1,1-trifluoropropan-2-yl)propanamide. To a 100 mL round bottom flask, containing a stir bar, was added 4,5-dichlorobenzene-1,2-diamine (0.244 g, 1.00 mmol), 4-[methyl(1,1,1-trifluoropropan-2-yl)amino]-4-oxobutanoic acid (0.366 g, 1.80 mmol), 1,4-dioxane (10.0 mL) and polyphosphoric acid (1.70 g, 20.1 mmol). The reaction vessel was sealed with a septum, flushed with argon, and heated to 110 º C for 22 hours. The reaction mixture was cooled to room temperature, diluted with ethyl acetate and water and neutralized with sodium carbonate. The organic layer was washed with water and washed with brine, dried over sodium sulfate, filtered and concentrated *in vacuo*. This material was and purified by reverse phase medium pressure liquid chromatography (0 – 100 % methanol in 25 mM aqueous ammonium formate). Fractions containing product were combined, concentrated *in vacuo*, dissolved in ethyl acetate, washed with saturated aqueous sodium bicarbonate, brine, concentrated *in vacuo* and purified by normal phase medium pressure liquid chromatography (0 – 10 % methanol in dichloromethane and subsequently 0 – 100 % ethyl acetate in hexanes) to yield the final product (0.113 g, 12 % yield). ^{1}H NMR (500 MHz, DMSO-d_6, amide rotamers) δ 12.82 – 12.35 (m, 1H), 7.86 – 7.61 (m, 2H), 5.36 – 4.78 (m, 1H), 3.36 (s, 0.5H), 3.04 (t, J = 6.7 Hz, 2H), 2.96 (d, J = 9.5 Hz, 4H), 2.74 (s, 0.5H), 1.40 – 1.26 (m, 3H). ^{19}F NMR (470 MHz, DMSO-d_6) δ -72.97 (d, J = 8.8 Hz). HRMS ESI (+) calc'd for [M+H] = 368.0540, found = 368.0569.

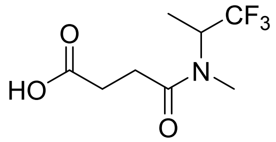

4-[methyl(1,1,1-trifluoropropan-2-yl)amino]-4-oxobutanoic acid. To a 100 mL round bottom flask, containing a stir bar, was added ethyl acetate (25.0 mL). Succinic anhydride was added as a solid (0.250 g, 2.50 mmol) and the reaction vessel was sealed, followed by N-methyl-1,1,1-trifluoro-2-propylamine hydrochloric acid salt (0.490 g, 3.00 mmol). The reaction vessel was sealed with a septum, flushed with argon, and heated to 50 º C. Triethylamine (0.909 g, 9.00 mmol) was added *via* syringe, the reaction mixture was stirred for 22 hours, cooled to room temperature and concentrated *in vacuo*, and used without purification in the next step.

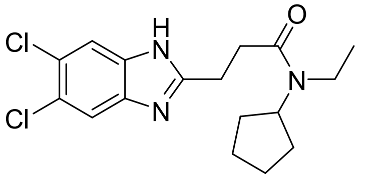

MSU-45538. *N*-cyclopentyl-3-(5,6-dichloro-1*H*-1,3-benzimidazol-2-yl)-*N*-ethylpropanamide. To a 100 mL round bottom flask, containing a stir bar, was added 4,5-dichlorobenzene-1,2-diamine (0.244 g, 1.00 mmol), 4-[cyclopentyl(ethyl)amino]-4-oxobutanoic acid (0.366 g, 1.80 mmol), 1,4-dioxane (10.0 mL) and polyphosphoric acid (1.70 g, 20.1 mmol). The reaction vessel was sealed with a septum, flushed with argon, and heated to 110 ° C for 22 hours. The reaction mixture was cooled to room temperature, diluted with ethyl acetate and water and neutralized with sodium carbonate. The organic layer was extracted with water and washed with brine, dried over sodium sulfate, filtered and concentrated *in vacuo*. This material was purified by methanol and purified by reverse phase medium pressure liquid chromatography (0 – 100 % methanol in 25 mM aqueous ammonium formate). Fractions containing product were combined, concentrated *in vacuo*, dissolved in ethyl acetate, washed with saturated aqueous sodium bicarbonate, brine, concentrated *in vacuo* and filtered through a plug of silica (eluted in 9:1 ethyl acetate:methanol) to obtain the pure product (0.329 g, 34 % yield). ^{1}H NMR (500 MHz, DMSO-d_6) δ 12.48 (s, 1H), 7.84 – 7.54 (m, 2H), 4.45 – 4.10 (m, 1H), 3.26 (q, J = 7.1 Hz, 1H), 3.13 (q, J = 6.9 Hz, 1H), 3.02 (q, J = 7.8 Hz, 2H), 2.92 – 2.80 (m, 2H), 1.78 (d, J = 9.8 Hz, 1H), 1.64 (s, 3H), 1.57 – 1.40 (m, 4H), 1.17 – 0.94 (m, 3H). HRMS calc'd for [M+H] = 354.1136, found = 354.1164.

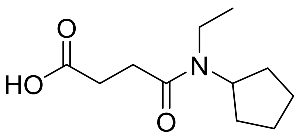

4-[cyclopentyl(ethyl)amino]-4-oxobutanoic acid. To a 100 mL round bottom flask, containing a stir bar, was added ethyl acetate (10.0 mL). Succinic anhydride was added as a solid (0.300 g, 3.00 mmol) and the reaction vessel was sealed, flushed with argon, and heated to 50 ° C. N-ethyl-N-cyclopentylamine (0.395 g, 3.50 mmol) was added *via* syringe. The reaction mixture was stirred for 22 hours, cooled to room temperature and concentrated *in vacuo*. This material was used in the next step without further purification.

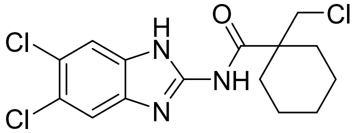

MSU-45606. 1-(chloromethyl)-*N*-(5,6-dichloro-1*H*-1,3-benzimidazol-2-yl)cyclohexane-1-carboxamide. To a 50 mL round bottom flask, containing a stir bar. was added 1-(chloromethyl)-1-cyclohexyl carboxylic acid (0.189 g, 1.12 mmol). The reaction vessel was sealed, flushed with argon, and N,N-dimethylformamide (1.00 mL) was added *via* syringe, followed by triethylamine (0.303 g, 3.00 mmol) and O-(7-azabenzotriazol-1-yl)-N,N,N',N'-tetramethyluronium hexafluorophosphate (0.456 g, 1.20 mmol). In a separate vial 5,6-dichloro-1*H*-benzo[d]imidazole-2-amine (0.262 g, 1.30 mmol) was dissolved in N,N-dimethylformamide (1.00 mL) and was added to the carboxylic acid containing solution by syringe. The reaction mixture was stirred for 18 hours,

then diluted with ethyl acetate, washed with water, 1.0 N aqueous hydrochloric acid, saturated aqueous sodium bicarbonate and brine. The organic layer is dried over sodium sulfate, filtered and concentrated *in vacuo*. The crude material is purified by silica gel chromatography (0 – 100 % ethyl acetate in hexanes followed by 0 – 10 % methanol in dichloromethane) to yield the final product (0.036 g, 9 % yield). ^{1}H NMR (500 MHz, DMSO-d_6) δ 12.43 (s, 1H), 11.55 (s, 1H), 7.70 – 7.58 (m, 2H), 3.96 (s, 2H), 2.17 (d, J = 13.5 Hz, 2H), 1.61 – 1.52 (m, 2H), 1.43 (q, J = 13.7 Hz, 5H), 1.32 – 1.21 (m, 1H). HRMS calc'd for [M+H] = 360.0433, found = 360.0469.

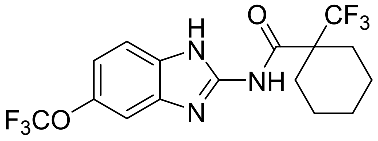

MSU-45819. 1-trifluoromethylmethyl-*N*-[5-(trifluoromethoxy)-1*H*-1,3-benzimidazol-2-yl]cyclohexane-1-carboxamide. To a 50 mL round bottom flask, containing a stir bar, was added 1-(trifluoromethyl)-1-cyclohexyl carboxylic acid (0.189 g, 1.12 mmol). The reaction vessel was sealed, flushed with argon, and N,N-dimethylformamide (1.00 mL) was added *via* syringe, followed by triethylamine (0.202 g, 2.00 mmol) and O-(7-azabenzotriazol-1-yl)-N,N,N',N'-tetramethyluronium hexafluorophosphate (0.437 g, 1.15 mmol). In a separate vial, 2-amino-5-(trifluoromethoxy)benzimidazole (0.260 g, 1.20 mmol) was dissolved in N,N-dimethylformamide (1.00 mL) and was added to the carboxylic acid containing solution by syringe. The reaction mixture was stirred for 20 hours at which time it was diluted with ethyl acetate, washed with water, 1.0 N aqueous hydrochloric acid, saturated aqueous sodium bicarbonate, and brine. The organic layer was dried over sodium sulfate, filtered and concentrated *in vacuo*. The crude material is purified by silica gel chromatography (0 – 10 % methanol in dichloromethane) to yield the product (0.149 g, 35 % yield). ^{1}H NMR (500 MHz, $CDCl_3$) δ 11.53 – 9.42 (m, 2H), 7.53 – 7.34 (m, 2H), 7.14 (dd, J = 8.7, 2.2 Hz, 1H), 2.41 – 2.32 (m, 2H), 1.79 (dt, J = 13.7, 3.7 Hz, 2H), 1.76 – 1.62 (m, 3H), 1.40 (qt, J = 13.3, 3.4 Hz, 2H), 1.25 (qt, J = 12.9, 3.6 Hz, 1H). ^{19}F NMR (470 MHz, $CDCl_3$) δ -58.23, -74.36. HRMS ESI (+) calc'd for [M+Na] = 418.0966, found = 418.0986.

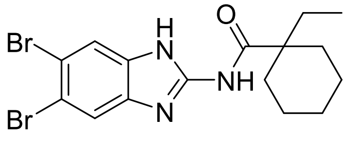

MSU-45350. *N*-(5,6-dibromo-1*H*-1,3-benzimidazol-2-yl)-1-ethylcyclohexane-1-carboxamide. To a 50 mL round bottom flask containing a stir bar was added 4,5-dibromo-2-aminobenzimidazole hydrobromic acid salt (0.706 g, 1.90 mmol), N,N-dimethyformamide (2.00 mL) and triethylamine (0.404 g, 4.00 mmol). The reaction vessel was sealed, flushed with argon, and heated to 50 º C with stirring. In a separate vial was added 1-ethyl-1-cyclohexyl carboxylic acid (0.268 g, 1.72 mmol), N,N-dimethylformamide (2.00 mL), triethylamine (0.202 g, 2.00 mmol) and O-(7-azabenzotriazol-1-yl)-N,N,N',N'-tetramethyluronium hexafluorophosphate (0.684 g, 1.80 mmol). This mixture was stirred until all solids dissolved and added to the

aminobenzimidazole solution *via* syringe. The combined mixture was stirred for 21 hours at 50 ^{o}C at which time it was cooled to room temperature, diluted with ethyl acetate and washed with water, brine, dried over sodium sulfate, filtered, concentrated *in vacuo* and purified by reverse phase medium pressure liquid chromatography (0 – 100 % methanol in 25 mM aqueous ammonium formate). Fractions containing product were combined, concentrated *in vacuo*, dissolved in ethyl acetate, washed with saturated aqueous sodium bicarbonate, brine, concentrated *in vacuo* and further purified by silica gel chromatography (0 – 100 % ethyl acetate in hexanes) to yield the final product (0.21 g, 29 % yield). ^{1}H NMR (500 MHz, DMSO-d_6) δ 2.19 (d, *J* = 13.2 Hz, 2H), 1.64 (q, *J* = 7.5 Hz, 2H), 1.59 – 1.46 (m, 3H), 1.29 (dt, *J* = 39.3, 12.8 Hz, 5H), 0.73 (t, *J* = 7.5 Hz, 3H). HRMS ESI (+) calc'd for [M+H] = 427.9969, found = 427.9987.
